# BASiCS workflow: a step-by-step analysis of expression variability using single cell RNA sequencing data

**DOI:** 10.12688/f1000research.74416.1

**Published:** 2022-01-18

**Authors:** Alan O'Callaghan, Nils Eling, John C. Marioni, Catalina A. Vallejos

**Affiliations:** 1 MRC Human Genetics Unit, Institute of Genetics & Cancer, University of Edinburgh, Edinburgh, EH4 2XU, UK; 2 Department of Quantitative Biomedicine, University of Zurich, Zürich, CH-8057, Switzerland; 3 Institute for Molecular Health Sciences, ETH Zürich, Zürich, 8093, Switzerland; 4 European Molecular Biology Laboratory, European Bioinformatics Institute, Cambridge, CB10 1SD, UK; 5 Cancer Research UK Cambridge Institute, University of Cambridge, Cambridge, CB2 0RE, UK; 6 The Alan Turing Institute, The Alan Turing Institute, London, NW1 2DB, UK

**Keywords:** single-cell RNA sequencing, expression variability, transcriptional noise, differential expression testing, scRNAseq, Bayesian, bioinformatics, heterogeneity

## Abstract

Cell-to-cell gene expression variability is an inherent feature of complex biological systems, such as immunity and development. Single-cell RNA sequencing is a powerful tool to quantify this heterogeneity, but it is prone to strong technical noise. In this article, we describe a step-by-step computational workflow that uses the BASiCS Bioconductor package to robustly quantify expression variability within and between known groups of cells (such as experimental conditions or cell types). BASiCS uses an integrated framework for data normalisation, technical noise quantification and downstream analyses, propagating statistical uncertainty across these steps. Within a single seemingly homogeneous cell population, BASiCS can identify highly variable genes that exhibit strong heterogeneity as well as lowly variable genes with stable expression. BASiCS also uses a probabilistic decision rule to identify changes in expression variability between cell populations, whilst avoiding confounding effects related to differences in technical noise or in overall abundance. Using a publicly available dataset, we guide users through a complete pipeline that includes preliminary steps for quality control, as well as data exploration using the scater and scran Bioconductor packages. The workflow is accompanied by a Docker image that ensures the reproducibility of our results.

## Introduction

Single-cell RNA-sequencing (scRNA-seq) enables the study of genome-wide cell-to-cell transcriptional heterogeneity that is not captured by bulk experiments. ^1^^–^^3^ On the broadest level, this heterogeneity can reflect the presence of distinct cell subtypes or states. Alternatively, it can be due to gradual changes along biological processes, such as development and differentiation. Several clustering and pseudotime inference tools have been developed to capture these types of heterogeneity. ^4^^,^^5^ However, there is a limited availability of computational tools tailored to study more subtle variability within seemingly homogeneous cell populations. This variability can reflect deterministic or stochastic events that regulate gene expression and, among other settings, has been seen to increase prior to cell fate decisions ^6^ and during ageing. ^7^ Transcriptional variability has also been observed to differ from gene to gene and can be conserved across cell types and species. ^8^

Stochastic variability within a seemingly homogeneous cell population — often referred to as transcriptional *noise* — can arise from intrinsic and extrinsic sources. ^9^^,^^10^ Extrinsic noise refers to stochastic fluctuations induced by different dynamic cellular states (e.g. cell cycle, metabolism, intra/inter-cellular signalling). ^11^^–^^13^ In contrast, intrinsic noise arises from stochastic effects on biochemical processes such as transcription and translation. ^9^ Intrinsic noise can be modulated by genetic and epigenetic modifications (such as mutations, histone modifications, CpG island length and nucleosome positioning) ^14^^–^^16^ and usually occurs at the gene level. ^9^ Cell-to-cell gene expression variability estimates derived from scRNA-seq data capture a combination of these effects, as well as deterministic regulatory mechanisms. ^10^ Moreover, these variability estimates can also be inflated by the technical noise that is typically observed in scRNA-seq data. ^17^

Different strategies have been incorporated into scRNA-seq protocols to control or attenuate technical noise. For example, external RNA spike-in molecules (such as the set introduced by the External RNA Controls Consortium, ERCC ^18^) can be added to each cell’s lysate in a (theoretically) known fixed quantity. Spike-ins can assist quality control steps, ^19^ data normalisation ^20^ and can be used to infer technical noise. ^17^ Another strategy is to tag individual cDNA molecules using unique molecular identifiers (UMIs) before PCR amplification. ^21^ Reads that contain the same UMI can be collapsed into a single molecule count, attenuating technical variability associated to cell-to-cell differences in amplification and sequencing depth (these technical biases are not fully removed unless sequencing to saturation ^20^). However, despite the benefits associated to the use of spike-ins and UMIs, these are not available for all scRNA-seq protocols. ^22^

The Bioconductor package *BASiCS* implements a Bayesian hierarchical framework that accounts for both technical and biological sources of noise in scRNA-seq datasets. ^23^^–^^25^ *BASiCS* jointly performs data normalisation, technical noise quantification and downstream analyses, whilst propagating statistical uncertainty across these steps. These features are implemented within a probabilistic model that builds upon a negative binomial framework, a widely used distribution in the context of bulk and scRNA-seq experiments. ^26^^–^^28^ Critically, *BASiCS* enables the quantification of transcriptional variability within a population of cells, while accounting for the overall mean-variance relationship that typically arises in scRNA-seq data. ^29^ Furthermore, when available, *BASiCS* can also leverage extrinsic spike-in molecules to aid data normalisation.

This article complements existing scRNA-seq workflows based on the Bioconductor ecosystem (e.g. Refs. 30, 31), providing a detailed framework for transcriptional variability analyses using *BASiCS.* We describe a step-by-step workflow that uses *scater* ^19^ and *scran* ^30^ to perform quality control (QC) as well as initial exploratory analyses. Our analysis pipeline includes practical guidance to assess the convergence of the Markov Chain Monte Carlo (MCMC) algorithm that is used to infer model parameters in *BASiCS*, as well as recommendations to interpret and post-process the model outputs. Finally, through a case study in the context of mouse immune cells, we illustrate how *BASiCS* can identify highly and lowly variable genes within a cell population, as well as to compare expression profiles between experimental conditions or cell types.

All source code used to generate the results presented in this article is available in Github and Zenodo. ^32^ To ensure the reproducibility of this workflow, the analysis environment and all software dependencies are provided as a Docker image. ^33^ The image can be obtained from Docker Hub.

## Methods

### Implementation

The *BASiCS* Bioconductor package uses a Bayesian hierarchical framework that borrows information across all genes and cells to robustly quantify transcriptional variability. ^34^ Similar to the approach adopted in *scran*, *BASiCS* infers cell-specific global scaling normalisation parameters. However, instead of inferring these as a pre-processing step, *BASiCS* uses an integrated approach wherein data normalisation and downstream analyses are performed simultaneously, thereby propagating statistical uncertainty. To quantify technical noise, the original implementation of *BASiCS* uses information from extrinsic spike-in molecules as control features, but the model has been extended to address situations wherein spike-ins are not available. ^29^

*BASiCS* summarises the expression pattern for each gene through gene-specific *mean* and *over-dispersion* parameters. Mean parameters $\mu_{i}$ quantify the overall expression for each gene i across the cell population under study. In contrast, $\delta_{i}$ captures the excess of variability that is observed with respect to what would be expected in a homogeneous cell population, beyond technical noise. *BASiCS* uses $\delta_{i}$ as a proxy to quantify transcriptional variability. To account for the strong relationship that is typically observed between gene-specific mean expression and over-dispersion estimates, Eling *et al.* ^29^ introduced a joint prior specification for these parameters. This joint prior has been observed to improve posterior inference when the data is less informative (e.g. small sample size, lowly expressed genes), borrowing information across all genes (and cells) to infer an overall trend that captures the relationship between mean and over-dispersion. The trend is then used to derive gene-specific *residual over-dispersion* parameters $\varepsilon_{i}$ that are not confounded by mean expression. Similar to the DM values implemented in *scran*, these are defined as deviations with respect to the overall trend.

Within a population of cells, *BASiCS* decomposes the total observed variability in expression measurements into technical and biological components. ^23^ This enables the identification of *highly variable genes* (HVGs) that capture the major sources of heterogeneity within the analysed cells. ^17^ HVG detection is often used as feature selection, to identify the input set of genes for subsequent analyses. *BASiCS* can also highlight *lowly variable genes* (LVGs) that exhibit stable expression across the population of cells. These may relate to essential cellular functions and can assist the development of new data normalisation or integration strategies. ^8^

*BASiCS* also provides a probabilistic decision rule to perform differential expression analyses between two pre-specified groups of cells. ^24^^,^^29^ While several differential expression tools have been proposed for scRNA-seq data (e.g. Refs. 35, 36), some evidence suggests that these do not generally outperform popular bulk RNA-seq tools. ^37^ Moreover, most of these methods are only designed to uncover changes in overall expression, ignoring the more complex patterns that can arise at the single cell level. ^38^ Instead, *BASiCS* embraces the high granularity of scRNA-seq data, uncovering changes in cell-to-cell expression variability that are not confounded by differences in technical noise or in overall expression.

### Operation

**Figure 1.**
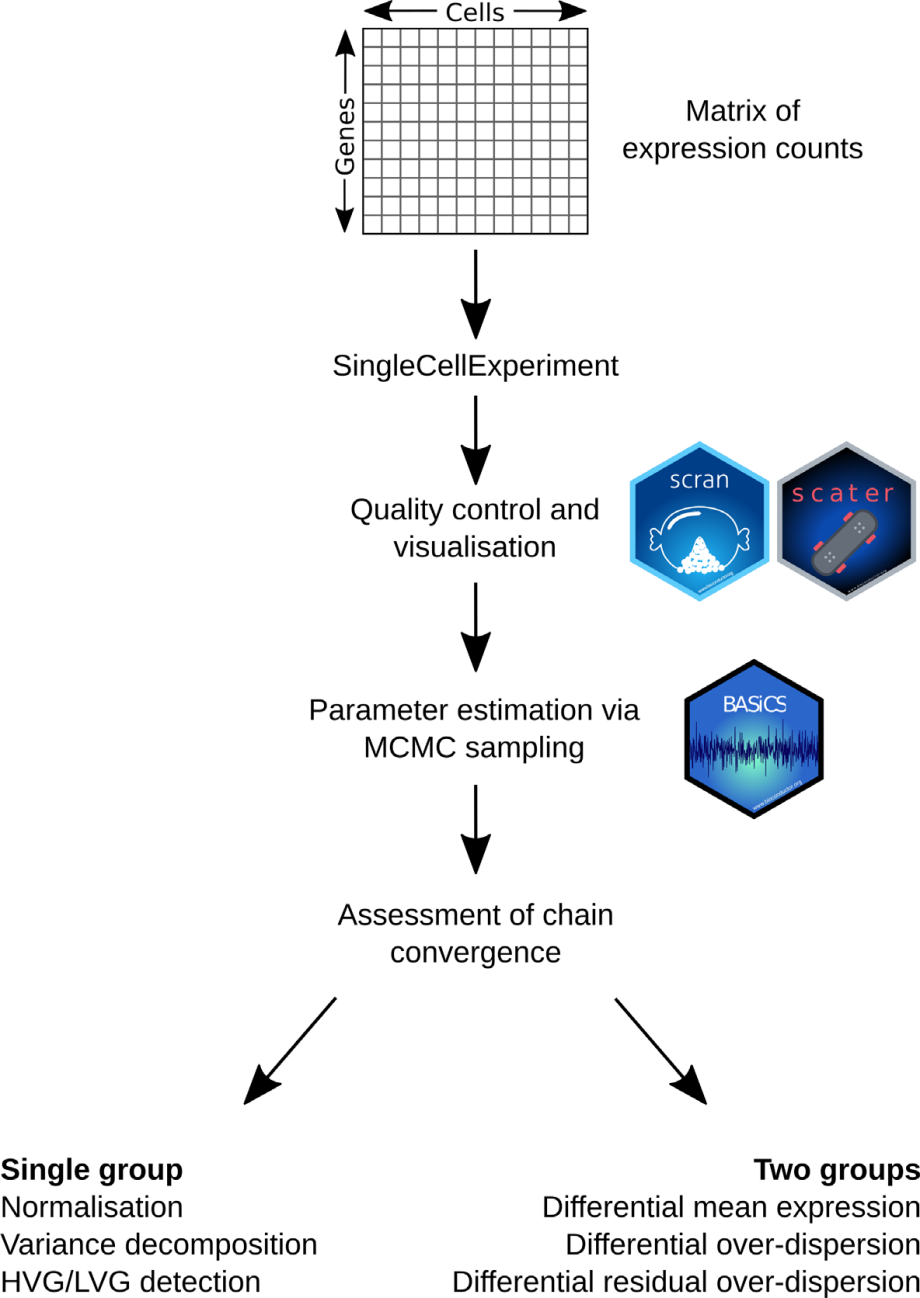
Graphical overview for the single cell RNA sequencing analysis workflow described in this manuscript. Starting from a matrix of expression counts, we use the scater and scran Bioconductor packages to perform QC and initial exploratory analyses. To robustly quantify transcriptional heterogeneity within seemingly homogeneous cell populations, we apply the BASiCS Bioconductor package and illustrate how BASiCS can be used to analyse a single or multiple pre-specified groups of cells.

This step-by-step scRNA-seq workflow is primarily based on the Bioconductor package ecosystem ^39^ for the R programming language, ^40^ and as such should run on any major operating system using R ≥ 4.0. A graphical overview is provided in Figure 1 and its main components are described below. The libraries listed below are required for this workflow, all of which can be downloaded from Bioconductor. Alternatively, we provide a Docker image containing all of the software necessary to run *BASiCS* at https://hub.docker.com/r/alanocallaghan/bocker/ .

library("SingleCellExperiment")
library("scater")
library("scran")
library("BASiCS")


We use the package *SingleCellExperiment* to convert an input matrix of raw read counts (molecule counts for UMI-based protocols) into a SingleCellExperiment object that can also store its associated metadata, such as gene- and cell-specific information. Moreover, when available, the same object can also store read counts for spike-in molecules (see help("altExp")). A major advantage of using a SingleCellExperiment object as the input for scRNA-seq analyses is the interoperability across a large number of Bioconductor packages. ^39^

A critical step in scRNA-seq analyses is QC, removing low quality samples that may distort downstream analyses. In this step, we use QC diagnostics to identify and remove samples that correspond to broken cells, that are empty, or that contain multiple cells. ^41^ We also typically remove lowly expressed genes that represent less reliable information. The *OSCA* online book provides an extensive overview on important aspects of how to perform QC of scRNA-seq data, including exploratory analyses. ^39^

Here, we use the Bioconductor package *scater* ^19^ to calculate QC metrics for each cell (e.g. total read-count) and gene (e.g. percentage of zeroes across all cells), respectively. We also use the visualisation tools implemented in the *scater* to explore the input dataset and its associated QC diagnostic metrics. For further exploratory data analysis we use the Bioconductor package *scran.* ^30^ The latter can perform *global scaling* normalisation, calculating cell-specific scaling factors that capture global differences in read-counts across cells (e.g. due to sequencing depth and PCR amplification). ^42^ To quantify transcriptional variability, *scran* can infer an overall trend between mean expression and the squared coefficent of variation (CV ^2^) for each gene. Variability estimates that are not confounded by this trend are then obtained via the DM approach. ^43^ For each gene, these are defined as the distance between CV ^2^ and a rolling median along the range of mean expression values. DM estimates enable exploratory analyses of transcriptional variability, but a measure of statistical uncertainty is not readily available. As such, gene-specific downstream inference (such as differential variability testing) is precluded.

## Use cases

### Case study: analysis of naive CD4 ^+^ T cells

As a case study, we use scRNA-seq data generated for CD4 ^+^ T cells using the C1 Single-Cell Auto Prep System (Fluidigm ^®^). Martinez-Jimenez *et al.* profiled naive (hereafter also referred to as unstimulated) and activated (three hours using *in vitro* antibody stimulation) CD4 ^+^ T cells from young and old animals across two mouse strains to study changes in expression variability during ageing and upon immune activation. ^7^ They extracted naive or effector memory CD4 ^+^ T cells from spleens of young or old animals, obtaining purified populations using either magnetic-activated cell sorting (MACS) or fluorescence activated cell sorting (FACS). External ERCC spike-in RNA ^18^ was added to aid the quantification of technical variability across all cells and all experiments were performed in replicates (hereafter also referred to as batches).

### Downloading the data

The matrix with raw read counts can be obtained from ArrayExpress under the accession number E-MTAB-4888. In the matrix, column names contain library identifiers and row names display Ensembl gene identifiers.

if (!file.exists("downloads/"))
 dir.create("downloads", showWarnings = FALSE)
if (!file.exists("downloads/raw_data.txt")) {
 website <- "
https://www.ebi.ac.uk/arrayexpress/files/E-MTAB-4888/
"
 file <- "E-MTAB-4888.processed.1.zip"
 download.file(
   paste0(website, file),
   destfile = "downloads/raw_data.txt.zip"
 )
 unzip("downloads/raw_data.txt.zip", exdir = "downloads")
 file.remove("downloads/raw_data.txt.zip")
}
cd4_raw <- read.table("downloads/raw_data.txt", header = TRUE, sep = "\t")
cd4_raw <- as.matrix(cd4_raw)


The input matrix contains data for 1,513 cells and 31,181 genes, including 92 ERCC spike-ins.

Information about experimental conditions and other metadata is available under the same accession number.

if (!file.exists("downloads/metadata_file.txt")) {
 website <- "
https://www.ebi.ac.uk/arrayexpress/files/E-MTAB-4888/
"
 file <- "E-MTAB-4888.additional.1.zip"
 download.file(
   paste0(website, file),
   destfile = "downloads/metadata.txt.zip"
 )
 unzip("downloads/metadata.txt.zip", exdir = "downloads")
 file.remove("downloads/metadata.txt.zip")
}
cd4_metadata <- read.table(
 "downloads/metadata_file.txt",
 header = TRUE,
 sep = "\t"
)
## Save sample identifiers as rownames
rownames(cd4_metadata) <- cd4_metadata$X


The columns in the metadata file contain library identifiers ( X), strain information ( Strain; *Mus musculus castaneus* or *Mus musculus domesticus*), the age of the animals ( Age; young or old), stimulation state of the cells ( Stimulus; naive or activated), batch information ( Individuals; associated to different mice), and cell type information ( Celltype; via FACS or MACS purification).

Here, we convert the data and metadata described above into a SingleCellExperiment object. For this purpose, we first separate the input matrix of expression counts into two matrices associated to intrinsic genes and external spike-ins, respectively. Within the SingleCellExperiment object, the latter is stored separately as an *alternative experiment.* For more details on the alternative experiment slot, see help("altExp").

## Separate intrinsic from ERCC counts
bio_counts <- cd4_raw[!grepl("ERCC", rownames (cd4_raw)),]
spike_counts <- cd4_raw[grepl("ERCC", rownames (cd4_raw)),]
## Generate the SingleCellExperiment object
sce_cd4_all <- SingleCellExperiment(
 assays = list(counts = bio_counts),
 colData = cd4_metadata[colnames (cd4_raw),]
)
## Add read-counts for spike-ins as an alternative experiment
altExp(sce_cd4_all, "spike-ins") <- SummarizedExperiment(
 assays = list(counts = spike_counts)
)


Hereafter, our analysis focuses on naive CD4 ^+^ T cells in the presence and absence of stimulation using plate-bound antibodies,+ obtained from young *Mus musculus domesticus* animals, and purified using MACS-based cell sorting. Thus, we subset the SingleCellExperiment object to these 146 cells.

ind_select <- sce_cd4_all$Strain == "Mus musculus domesticus" &
 sce_cd4_all$Age == "Young" &
 sce_cd4_all$Celltype == "MACS-purified Naive"
sce_naive_active <- sce_cd4_all[, ind_select]
sce_naive_active

## class: SingleCellExperiment
## dim: 31089 146
## metadata(0):
## assays(1): counts
## rownames(31089): ENSMUSG00000000001 ENSMUSG00000000003 …
##  ENSMUSG00000106668 ENSMUSG00000106670
## rowData names(0):
## colnames(146): do6113 do6118 … do6493 do6495
## colData names(6): X Strain … Individuals Celltype
## reducedDimNames(0):
## mainExpName: NULL
## altExpNames(1): spike-ins


### Annotation

Input data was annotated using Ensembl gene identifiers. To facilitate interpretation, it is often useful to obtain a mapping from Ensembl gene IDs to gene symbols using the BioMart suite ( http://www.biomart.org ) via the Bioconductor package *biomaRt.* ^44^ This can also be used to obtain gene-pathways mappings and other metadata (e.g. gene length), useful for performing functional analysis of gene sets identified in downstream analyses.

library("biomaRt")
if (!dir.exists("rds")) {
 dir.create("rds", showWarnings = FALSE)
}
if (!file.exists("rds/genenames.rds")) {
 # Initialize mart and dataset
 ensembl <- useEnsembl(
   biomart = "genes",
   version = 104,
   dataset = "mmusculus_gene_ensembl"
 )
 # Select gene ID and gene name
 genenames <- getBM(
   attributes = c("ensembl_gene_id", "external_gene_name", "gene_biotype"),
   mart = ensembl
 )
 rownames(genenames) <- genenames$ensembl_gene_id
 saveRDS(genenames, "rds/genenames.rds")
}
genenames <- readRDS("rds/genenames.rds")


We add this information as rowData within the SingleCellExperiment object created above.

## Merge biomaRt annotation
rowdata <- data.frame(ensembl_gene_id = rownames(sce_naive_active))
rowdata <- merge(rowdata, genenames, by = "ensembl_gene_id", all.x = TRUE)
rownames(rowdata) <- rowdata$ensembl_gene_id
## Check if order is correct after merge;
stopifnot(all(rownames(rowdata) == rownames(sce_naive_active)))
## add to the SingleCellExperiment object
rowData(sce_naive_active) <- rowdata


For the remaining analysis, we will only focus on the 18,682 protein coding genes that are contained in the data. These are selected below.

protein_coding <- which(
 rowData(sce_naive_active)$gene_biotype == "protein_coding"
)
sce_naive_active <- sce_naive_active[protein_coding,]


### QC and exploratory data analysis

**Figure 2.**
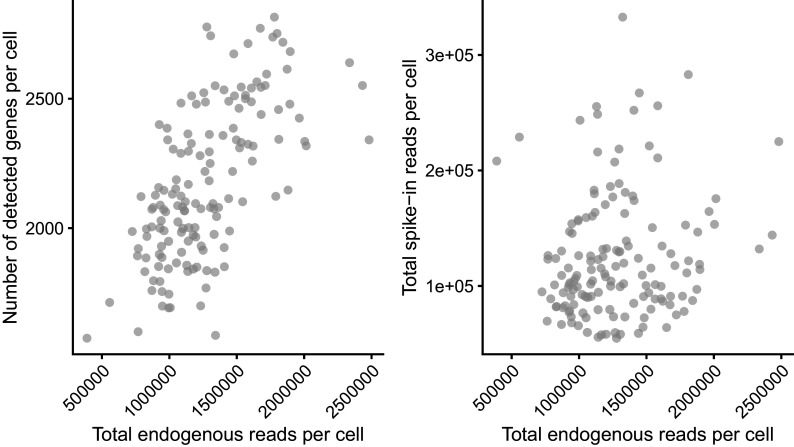
Cell-level quality control metrics. The total number of endogenous read-counts (excludes non-mapped and intronic reads) is plotted against the total number of detected genes (left) and the total number of spike-in read-counts (right).

The data available at E-MTAB-4888 have been filtered already to remove poor quality samples. The QC applied in Ref. 7 removed cells with: (i) fewer than 1,000,000 total reads, (ii) less than 20% of reads mapped to endogenous genes, (iii) less than 1,250 or more than 3,000 detected genes and (iv) more than 10% or fewer than 0.5% of reads mapped to mitochondrial genes. We include visualisations of these measures here; we also include another widely used QC diagnostic plot that compares the total number (or fraction) of spike-in counts versus the total number (or fraction) of endogeneous counts. In such a plot, low quality samples are characterised by a high fraction of spike-in counts and a low fraction of endogeneous counts (see Figure 2).

sce_naive_active <- addPerCellQC (sce_naive_active, use_altexps = TRUE)
p_cell_qc1 <- plotColData(
 sce_naive_active,
 x = "sum",
 y = "detected") +
 xlab("Total endogenous reads per cell") +
 ylab("Number of detected genes per cell") +
 theme(axis.text.x = element_text(hjust = 1, angle = 45))
p_cell_qc2 <- plotColData(
 sce_naive_active,
 x = "sum",
 y = "altexps_spike-ins_sum") +
 xlab("Total endogenous reads per cell") +
 ylab("Total spike-in reads per cell") +
 theme(axis.text.x = element_text(hjust = 1, angle = 45))
library("patchwork")
p_cell_qc1 + p_cell_qc2


**Figure 3.**
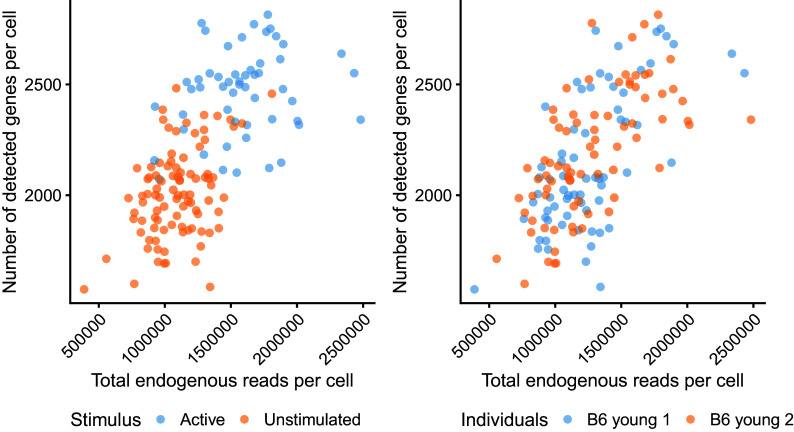
Cell-level quality control metrics according to cell-level metadata. The total number of endogenous reads (excludes non-mapped and intronic reads) is plotted against the total number of detected genes. Colour indicates the experimental condition (left) and animal of origin (right) for each cell.

We can also visualise these metrics with respect to cell-level metadata, such as the experimental conditions (active vs unstimulated) and the different mice from which cells were collected (see Figure 3).

p_stimulus <- plotColData(
   sce_naive_active,
   x = "sum",
   y = "detected",
   colour_by = "Stimulus"
 ) +
 xlab("Total endogenous reads per cell") +
 ylab("Number of detected genes per cell") +
 theme(
   legend.position = "bottom",
   axis.text.x = element_text(angle = 45, hjust = 1)
 )
p_batch <- plotColData(
   sce_naive_active,
   x = "sum",
   y = "detected",
   colour_by = "Individuals"
 ) +
 xlab("Total endogenous reads per cell") +
 ylab("Number of detected genes per cell") +
 theme(
   legend.position = "bottom",
   axis.text.x = element_text(angle = 45, hjust = 1)
 )
p_stimulus + p_batch


**Figure 4.**
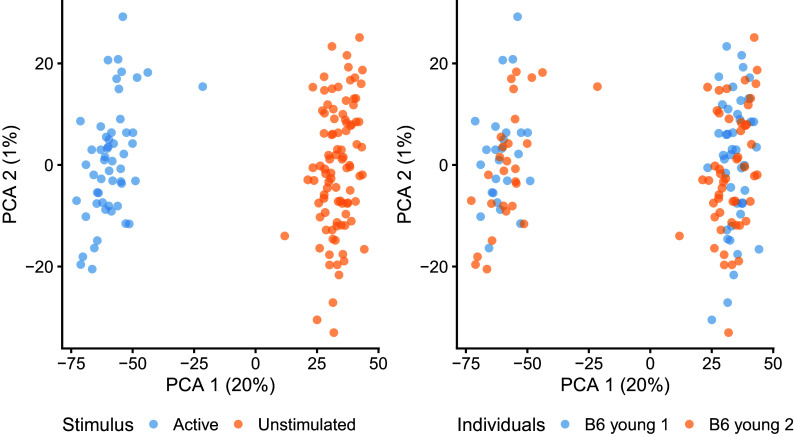
First two principal components of log-transformed expression counts after scran normalisation. Colour indicates the experimental condition (left) and animal of origin (right) for each cell.

To further explore the underlying structure of the data, we perform global scaling normalisation using *scran* and principal component analysis (PCA) of log-transformed normalised expression counts using *scater.* As seen in Figure 4, this analysis suggests the absence of strong batch effects. It should be noted that the estimation of global scaling normalisation factors using *scran* is not strictly necessary in the *BASiCS* workflow. Here, we only use it as part of the exploratory data analysis. Moreover, count-based models for dimensionality reduction (e.g. Refs. 28, 45) could be used as an alternative to PCA, removing the need for log normalisation.

## Global scaling normalisation + log tranformation + PCA
sce_naive_active <- computeSumFactors(sce_naive_active)
sce_naive_active <- logNormCounts(sce_naive_active)
sce_naive_active <- runPCA(sce_naive_active)
p_stimulus <- plotPCA(sce_naive_active, colour_by = "Stimulus") +
 theme(legend.position = "bottom")
p_batch <- plotPCA(sce_naive_active, colour_by = "Individuals") +
 theme(legend.position = "bottom")
p_stimulus + p_batch


**Figure 5.**
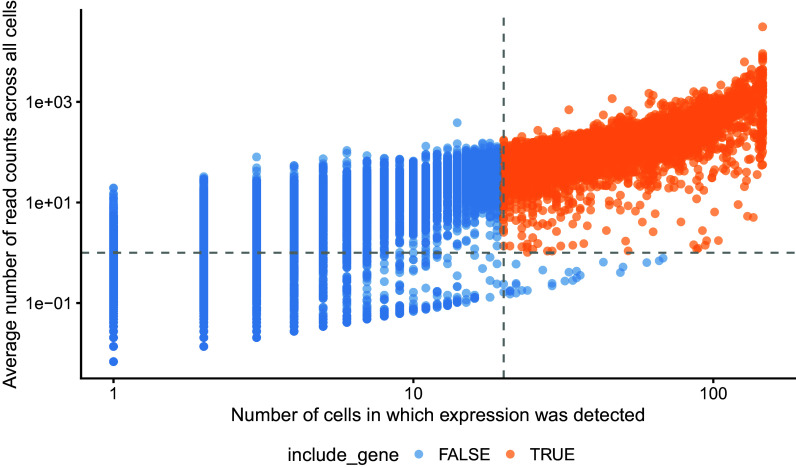
Average read-count for each gene is plotted against the number of cells in which that gene was detected. Dashed grey lines are shown at the thresholds below which genes are removed.

In addition to cell-specific QC, we also recommend a gene filtering step prior to using *BASiCS.* The purpose of this filter is to remove lowly expressed genes that were largely undetected through sequencing, making reliable variability estimates difficult to obtain. Here, we remove genes that are not detected in at least 20 cells across both conditions, or that have an average read count below 1. These thresholds can vary across datasets and should be informed by gene-specific QC metrics such as those shown in Figure 5 as well as prior knowledge about the cell types and conditions being studied, where available.

sce_naive_active <- addPerFeatureQC(sce_naive_active, exprs_values = "counts")
## Remove genes with zero total counts across all cells
sce_naive_active <- sce_naive_active[rowData(sce_naive_active)$detected != 0,]
## Transform "detected" into number of cells and define inclusion criteria
rowData(sce_naive_active)$detected_cells <-
 rowData(sce_naive_active)$detected * ncol(sce_naive_active) / 100
detected_threshold <- 20
mean_threshold <- 1
include_gene <- rowData(sce_naive_active)$mean >= mean_threshold &
 rowData(sce_naive_active)$detected_cells >= detected_threshold
rowData(sce_naive_active)$include_gene <- include_gene
plotRowData(
   sce_naive_active,
   x = "detected_cells",
   y = "mean",
   colour_by = "include_gene"
 ) +
 xlab("Number of cells in which expression was detected") +
 ylab("Average number of read counts across all cells") +
 scale_x_log10() +
 scale_y_log10() +
 theme(legend.position = "bottom") +
 geom_vline(
   xintercept = detected_threshold,
   linetype = "dashed",
   col = "grey60"
 ) +
 geom_hline(
   yintercept = mean_threshold,
   linetype = "dashed",
   col = "grey60"
 )

## Apply gene filter
sce_naive_active <- sce_naive_active[rowData(sce_naive_active)$include_gene,]


Subsequently, we also require users to remove spike-in molecules that were not captured through sequencing. We do this separately for naive and active cells.

ind_active <- sce_naive_active$Stimulus == "Active"
ind_naive <- sce_naive_active$Stimulus == "Unstimulated"
spikes <- assay(altExp(sce_naive_active))
detected_spikes_active <- rowSums(spikes[, ind_active] > 0) > 0
detected_spikes_naive <- rowSums(spikes[, ind_naive] > 0) > 0
detected_spikes <- detected_spikes_naive & detected_spikes_active
altExp(sce_naive_active) <- altExp(sce_naive_active)[detected_spikes,]


The final dataset used in subsequent analyses contains 146 cells, 5171 genes and 49 spike-ins.

### Input data for BASiCS

Here, we apply *BASiCS* separately to cells from each experimental condition (93 naive and 53 activated cells). We create separate SingleCellExperiment objects for each group of cells.

sce_naive <- sce_naive_active[, ind_naive]
sce_active <- sce_naive_active[, ind_active]


*BASiCS* requires these objects to be augmented with extra information in a specific format. If multiple batches of sequenced cells are available (e.g. multiple donors from which cells were extracted, sequencing batches from the same experimental condition), this must be indicated under the BatchInfo label as cell-level metadata.

colData(sce_naive)$BatchInfo <- colData(sce_naive)$Individuals
colData(sce_active)$BatchInfo <- colData(sce_active)$Individuals


If spike-ins will be used to aid data normalisation and technical noise quantification, *BASiCS* also requires the number of spike-in molecules that were added to each well. For each spike-in i , this corresponds to:

$$\mu_{i}=C_{i}\times 10^{-18}\times \left(6.022\times 10^{23}\right)\times V\times D\;\text{where},$$

•
$C_{i}$ is the concentration for the spike-in i (measured in $\mathrm{aM\mu}l^{-1}$ ),
•
V is the volume added into each well (measure in nl ) and
•
D is a dilution factor.

The remaining factors in the equation above are unit conversion constants (e.g. from moles to molecules). For the CD4+ T cell data, the authors added a 1:50,000 dilution of the ERCC spike-in mix 1 and a volume of 9nl was added into each well. Finally, input concentrations $C_{i}$ can be downloaded from https://assets.thermofisher.com/TFS-Assets/LSG/manuals.

if (!file.exists("downloads/spike_info.txt")) {
 website <- "
https://assets.thermofisher.com/TFS-Assets/LSG/manuals/
"
 file <- "cms_095046.txt"
 download.file(
   paste0(website, file),
   destfile = "downloads/spike_info.txt"
 )
}
ERCC_conc <- read.table("downloads/spike_info.txt", sep = "\t", header = TRUE)


Based on this information, the calculation above proceeds as follows

## Moles per micro litre
ERCC_mmul <- ERCC_conc$concentration.in. Mix.1.attomoles.ul. * (10ˆ(-18))
## Molecule count per microL (1 mole comprises 6.02214076 x 10ˆ{23} molecules)
ERCC_countmul <- ERCC_mmul * (6.02214076 * (10ˆ23))
## Application of the dilution factor (1:50,000)
ERCC_count <- ERCC_countmul / 50000
## Multiplying by the volume added into each well
ERCC_count_final <- ERCC_count * 0.009


To update the sce_naive and sce_active objects, the user must create a data.frame whose first column contains the spike-in labels (e.g. ERCC-00130) and whose second column contains the number of molecules calculated above. We add this as row metadata for altExp (sce_naive) and altExp (sce_active).

SpikeInput <- data.frame(
 Names = ERCC_conc$ERCC.ID,
 count = ERCC_count_final
)
## Exclude spike-ins not included in the input SingleCellExperiment objects
SpikeInput <- SpikeInput[match(rownames(altExp(sce_naive)), SpikeInput$Names),]
## Add as metadata
rowData(altExp(sce_naive)) <- SpikeInput
rowData(altExp(sce_active)) <- SpikeInput


### Parameter estimation using BASiCS

Parameter estimation is implemented in the BASiCS_MCMC function using an adaptive Metropolis within Gibbs algorithm (see section 3 in Ref. 46). The primary inputs for BASiCS_MCMC correspond to:

• Data: a SingleCellExperiment object created as described in the previous sections.
• N: the total number of MCMC iterations.
• Thin: thining period for output storage (only the Thin-th MCMC draw is stored).
• Burn: the initial number of MCMC iterations to be discarded.
• Regression: if TRUE a joint prior is assigned to $\mu_{i}$ and $\delta_{i}$ _,_^29^ and residual over-dispersion values $\varepsilon_{i}$ are inferred. Alternatively, independent log-normal priors are assigned to $\mu_{i}$ and $\delta_{i}$ _._^24^
• WithSpikes: if TRUE information from spike-in molecules is used to aid data normalisation and to quantify technical noise.
• PriorParam: Defines the prior hyper-parameters to be used by *BASiCS.* We recommend to use the BASiCS_PriorParam function for this purpose. If PriorMu = "EmpiricalBayes", $\mu_{i}$ ’s are assigned a log-normal prior with gene-specific location hyper-parameters defined via an empirical Bayes framework. Alternatively, if PriorMu = "default", location hyper-parameters are set to be equal 0 for all genes.

As a default, we recommend to use Regression = TRUE, as the joint prior introduced by Ref. 29 leads to more stable estimation, particularly for small sample sizes and lowly expressed genes. This approach also enables users to obtain a measure of transcriptional variability that is not confounded by mean expression. We also recommend to use PriorMu = "EmpiricalBayes" as we have observed that an empirical Bayes framework ^47^ improves estimation performance for sparser datasets. Extra parameters can be used to store the output ( StoreChains, StoreDir, RunName) and to monitor the progress of the algorithm ( PrintProgress).

Here, we run the MCMC sampler separately for naive and activated cells. We use 40,000 iterations ( N), discarding the initial 20,000 iterations ( Burn), and saving parameter values only once in each 20 iterations ( Thin). We recommend this setting as a default choice, as we have observed it to ensure good convergence across multiple datasets. However, fewer iterations may be sufficient for larger and less sparse datasets, and may be more feasible computationally for larger datasets. Practical guidance about MCMC convergence diagnostics is provided in the next section.

## MCMC results may vary slightly due to pseudorandom number generation.
## We fix a random seed for exact reproducibility,
## but this is not strictly required
set.seed(42)
chain_naive <- BASiCS_MCMC(
 Data = sce_naive,
 N = 40000,
 Thin = 20,
 Burn = 20000,
 Regression = TRUE,
 WithSpikes = TRUE,
 PriorParam = BASiCS_PriorParam(sce_naive, PriorMu = "EmpiricalBayes"),
 Threads = 4,
 StoreChains = TRUE,
 StoreDir = "rds/",
 RunName = "naive"
)
set.seed(43)
chain_active <- BASiCS_MCMC(
 Data = sce_active,
 N = 40000,
 Thin = 20,
 Burn = 20000,
 Regression = TRUE,
 WithSpikes = TRUE,
 PriorParam = BASiCS_PriorParam(sce_active, PriorMu = "EmpiricalBayes"),
 Threads = 4,
 StoreChains = TRUE,
 StoreDir = "rds/",
 RunName = "active"
)


This first of these samplers takes 84 minutes to complete on a 3.4 GHz Intel Core i7 4770k procesor with 32GB RAM, while the second takes 69 minutes. For convenience, these can be obtained online at https://doi.org/10.5281/zenodo.5243265 .

chains_website <- "
https://zenodo.org/record/5243265/files/
"
options(timeout = 1000)
if (!file.exists("rds/chain_naive.Rds")) {
 download.file(
   paste0(chains_website, "chain_naive.Rds"),
   destfile = "rds/chain_naive.Rds"
 )
}
if (!file.exists("rds/chain_active.Rds")) {
 download.file(
   paste0(chains_website, "chain_active.Rds"),
   destfile = "rds/chain_active.Rds"
 )
}
chain_naive <- readRDS("rds/chain_naive.Rds")
chain_active <- readRDS("rds/chain_active.Rds")


The output from BASiCS_MCMC is a BASiCS_Chain object that contains the draws associated to all model parameters. Given that N = 40,000, Thin = 20 and Burn = 20,000, the object contains 1,000 draws for each parameter. These can be accessed using the displayChainBASiCS function. For example, the following code displays the first 6 draws for mean expression parameters $\mu_{i}$ associated to the first 3 genes.

displayChainBASiCS (chain_naive, Param = "mu")[1:2, 1:3]

##    ENSMUSG00000000001 ENSMUSG00000000088 ENSMUSG00000000131
## [1,]       26.19096       1.759170       35.60811
## [2,]       12.05079       2.564187       35.58258


### MCMC diagnostics

Before interpreting the estimates generated by *BASiCS*, it is critical to assess the convergence of the MCMC algorithm, i.e. whether the MCMC reached its stationary distribution. If convergence has been achieved, the trace for each parameter should not evolve significantly across iterations, as MCMC draws are expected to be stochastic fluctuations around a horizontal trend once the sampler has converged to its stationary distribution. It is not possible to prove convergence, but multiple graphical and quantitative convergence diagnostics have been proposed to assess the lack of convergence (e.g. Refs. 48, 49). Some advocate the use of multiple MCMC chains using different starting values in order to ensure that the algorithm consistently converges to the same distribution and to avoid convergence to local modes. For *BASiCS*, we have observed that using informed starting values (e.g. based on *scran* normalisation factors) and a sufficiently large value for N and Burn generally leads to largely consistent estimates across multiple MCMC runs. Hence, the focus of this section is to evaluate quantitative measures of convergence (e.g. Ref. 50) based on a single MCMC chain.

**Figure 6.**
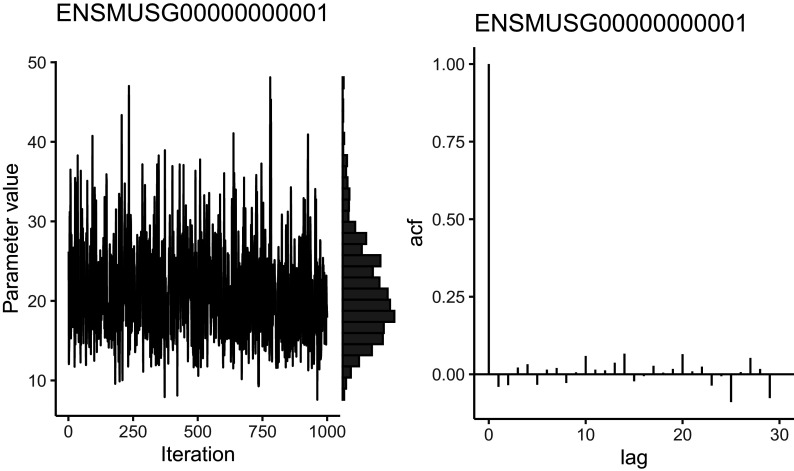
Trace plot, marginal histogram, and autocorrelation function of the posterior distribution of the mean expression parameter for a gene in naive cells following Markov chain Monte Carlo sampling. Trace plots should explore the posterior without getting stuck in one location or drifting over time towards a region of higher density. High autocorrelation indicates that the number of effective independent samples is low. It is good practice to perform this visualisation for many different parameters; here we only show one.

Traceplots can be used to visually assess the history of MCMC iterations for a specific parameter (e.g. Figure 6, left panel). As mentioned above, significant departures from a horizontal trend suggest a lack of convergence. As illustrated in Figure 6, histograms can also be used to display the marginal distribution for each parameter. For *BASiCS*, users should expect these to follow a unimodal distribution. Failure to satisfy these graphical diagnostics suggest that N and Burn must be increased. Alternatively, more stringent quality control could be applied to the input data, as we have observed that genes with very low counts often suffer from slow convergence.

plot (chain_naive, Param = "mu", Gene = 1)


**Figure 7.**
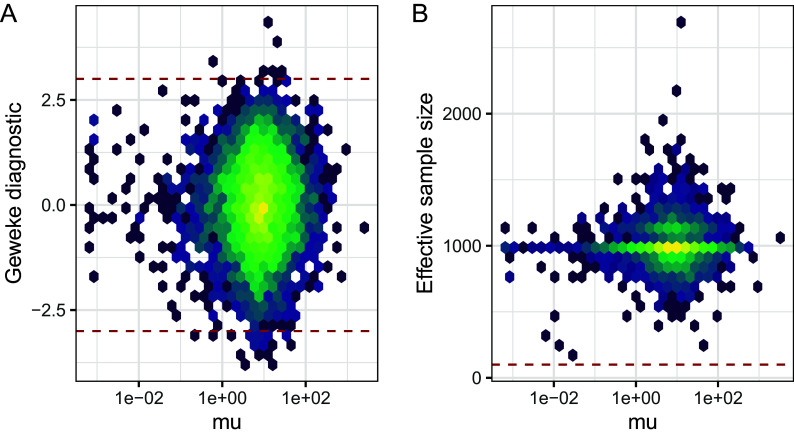
Markov chain Monte Carlo diagnostics for gene-specific mean expression parameters; naive CD4 ^+^ T cells. A: Geweke Z-score for mean expression parameters is plotted against mean expression estimates. Dashed lines represent absolute Z-scores of 3, outside of which we advise caution when interpreting results. B: Effective sample size (ESS) is plotted against mean expression estimates. A dashed line shows a threshold of 100, below which we advise caution when interpreting results.

As *BASiCS* infers thousands of parameters, it is impractical to assess these diagnostics separately for each parameter. Thus, it is helpful to use numerical diagnostics that can be applied to multiple parameters simultaneously. Here, we illustrate usage for two such metrics focusing on the MCMC chain that was obtained for the naive CD4 ^+^T cells (similar results were obtained for activated cells). First, we focus on the diagnostic criterion proposed by Geweke. ^50^ The latter compares the average of draws obtained during the initial (10% after burn in, by default) and the final part of the chain (50% by default) by calculating Z -scores of the relative difference between the two sets of draws. Large absolute Z -scores suggest that the algorithm has not converged (as a rule of thumb, a threshold at |Z|<3 is often applied). For the naive and activatived CD4 ^+^ T datasets most Z-scores associated to mean expression parameters $\mu_{i}$ were small in absolute value (see Figure 7A), suggesting that the algorithm has largely converged.

As well as assessing MCMC convergence, it is important to ensure that the MCMC algorithm has efficiently explored the parameter space. For example, the autocorrelation function (e.g. Figure 6, right panel) quantifies the correlation between the chain and its lagged versions. Strong autocorrelation indicates that neighbouring MCMC samples are highly dependent and suggest poor sampling efficiency. The latter may indicate that the MCMC draws do not contain sufficient information to produce accurate posterior estimates. In other words, highly correlated MCMC samplers require more samples to produce the same level of Monte Carlo error for an estimate (defined as the variance of a Monte Carlo estimate across repetitions ^51^).

The effective sample size (ESS) is a related measure which represents a proxy for the number of independent draws generated by the MCMC sampler. ^52^ The latter is defined as:

$$\;\mathrm{ESS}=\frac{N_{\mathrm{tot}}}{1+2\sum_{k=1}^{\infty }\rho \left(k\right)},$$

where $N_{\mathrm{tot}}$ represents the total number of MCMC draws (after burn-in and thining) and $\rho \left(k\right)$ is the autocorrelation at lag k . ESS estimates associated to mean expression parameters for the naive CD4 ^+^ T cells are displayed in Figure 7B. Whilst ESS is around 1,000 ( $N_{\mathrm{tot}}$ in this case) for most genes, we observe low ESS values for a small proportion of genes (primarily lowly expressed genes whose expression was only captured in a small number of cells). As described later in this manuscript, BASiCS_TestDE automatically excludes genes with low ESS during differential expression testing (by default a threshold at ESS <100 is applied). However, if a large number of genes have large Geweke diagnostic values or low effective sample sizes in a certain dataset, then caution should be applied when interpreting the results of the model. These issues can often be addressed by more stringent filtering of genes and cells before performing inference or by increasing the number of iterations.

library("coda")
library("ggplot2")
library("viridis")
diag_p1 <- BASiCS_DiagPlot(chain_naive, Measure = "geweke")
diag_p1 <- diag_p1 +
 geom_hline(yintercept = c(-3, 3), col = "firebrick", linetype = "dashed") +
 theme(legend.position = "none")
diag_p2 <- BASiCS_DiagPlot(chain_naive, Measure = "ess")
diag_p2 <- diag_p2 +
 geom_hline(yintercept = 100, col = "firebrick", linetype = "dashed") +
 theme(legend.position = "none")
diag_p1 + diag_p2 + plot_annotation(tag_levels = "A")


### Quantifying transcriptional variability using BASiCS

Studying gene-level transcriptional variability can provide insights about the regulation of gene expression, and how it relates to the properties of genomic features (e.g. CpG island composition ^16^), transcriptional dynamics ^53^ and aging, ^7^ among others. The squared coefficient of variation (CV ^2^) is widely used as a proxy for transcriptional variability. For example, we can obtain CV ^2^ estimates for each gene using *scran* normalised counts as input. In contrast, *BASiCS* infers transcriptional variability using gene-specific over-dispersion parameters $\delta_{i}$ (see *Methods*). Here, we compare these approaches, focusing on naive CD4 ^+^ T cells (repeating this analysis for active cells led to similar results).

**Figure 8.**
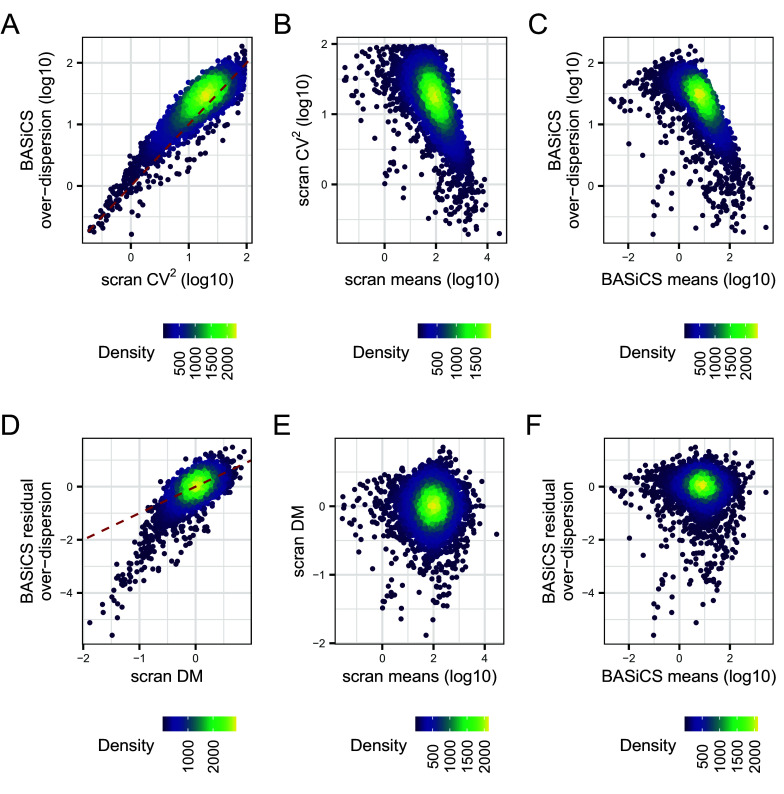
Comparison of gene-specific transcriptional variability estimates and mean expression estimates obtained for each gene using BASiCS and scran. For this analysis, we exclude genes that are not expressed in at least two cells. BASiCS estimates for each gene are defined by the posterior median of the associated parameter. scran estimates for each gene are derived after applying the pooling normalisation strategy proposed by Lun *et al*. Points are coloured according to the local density of genes along the x- and y-axis. A: scran squared CV estimates versus BASiCS estimates for over-dispersion parameters. B: scran estimates for mean expression and the squared CV. C: BASiCS estimates for mean expression and over-dispersion parameters. D: BASiCS estimates for residual over-dispersion parameters versus distance-to-median (DM) values estimated by scran. E: scran estimates for mean expression and DM values. F: BASiCS estimates for mean expression and residual over-dispersion parameters. Dashed red lines in panels A and D represent the line given by x = y.

As seen in Figure 8A, CV ^2^ and posterior estimates for $\delta_{i}$ are highly correlated. Moreover, both variability metrics are confounded by differences in mean expression, i.e. highly expressed genes tend to exhibit lower variability ( Figure 8B–C). To remove this confounding, *scran* and *BASiCS* derive *residual variability* estimates as deviations with respect to an global mean-variability trend (see *Methods*). These are derived using the DM approach ^43^ and the residual over-dispersion parameters $\varepsilon_{i}$ defined by Ref. 29, respectively. For the naive CD4 ^+^ T cell data, both approaches led to strongly correlated estimates ( Figure 8D) and, as expected, neither DM values nor posterior estimates for $\varepsilon_{i}$ are seen to be associated with mean expression ( Figure 8E–F). However, unlike the DM method, the integrated approach implemented in *BASiCS* provides a direct measure of statistical uncertainty for these estimates via posterior variance. Note that the BASiCS_ShowFit function can be used to generate Figure 8C, but we generated the plot manually to demonstrate how users can extract this information from a BASiCS_MCMC object, and for visual consistency with the other panels. For each of the panels in Figure 8, we use the R package *ggpointdensity* to visualise the local density of genes along the axes of mean and variability.

library("ggpointdensity")
library("viridis")
## Get BASiCS posterior estimates for mean and variability - naive cells
summary_naive <- Summary(chain_naive)
parameter_df <- data.frame(
 mu = displaySummaryBASiCS(summary_naive, Param = "mu")[, 1],
 delta = displaySummaryBASiCS(summary_naive, Param = "delta")[, 1],
 epsilon = displaySummaryBASiCS(summary_naive, Param = "epsilon")[, 1]
)
## Get scran estimates for mean and variability - naive cells
sce_naive <- logNormCounts(sce_naive, log = FALSE)
parameter_df$mean_scran <- rowMeans(assay(sce_naive, "normcounts"))
parameter_df$cv2_scran <- rowVars(assay(sce_naive, "normcounts")) /
 parameter_df$mean_scranˆ2
parameter_df$DM <- DM(
 mean = parameter_df$mean_scran,
 cv2 = parameter_df$cv2_scran
)
## Remove genes without counts in > 2 cells - BASiCS estimates not provided
ind_not_na <- !(is.na(parameter_df$epsilon))
plot_params <- list(
 geom_pointdensity(size = 0.6),
 scale_colour_viridis(name = "Density"),
 theme(
   text = element_text(size = rel(3)),
   legend.position = "bottom",
   legend.text = element_text(angle = 90, size = 8, hjust = 0.5, vjust = 0.5),
   legend.key.size = unit(0.018, "npc")
 )
)
g1 <- ggplot(parameter_df[ind_not_na,], aes (log10(cv2_scran), log10(delta))) +
 plot_params +
 xlab(bquote("scran"~CVˆ2~"(log10)")) +
 ylab("BASiCS\nover-dispersion (log10)") +
 geom_abline(
   slope = 1,
   intercept = 0,
   colour = "firebrick",
   linetype = "dashed"
 )
g2 <- ggplot(parameter_df[ind_not_na,],
   aes(log10(mean_scran), log10(cv2_scran))
 ) +
 plot_params +
 xlab("scran means (log10)") +
 ylab(bquote("scran"~CVˆ2~"(log10)"))
g3 <- ggplot (parameter_df[ind_not_na,], aes (log10(mu), log10(delta))) +
 plot_params +
 xlab("BASiCS means (log10)") +
 ylab("BASiCS\nover-dispersion (log10)")
g4 <- ggplot (parameter_df[ind_not_na,], aes (DM, epsilon)) +
 plot_params +
 xlab("scran DM") +
 ylab("BASiCS residual\nover-dispersion") +
 geom_abline(
   slope = 1,
   intercept = 0,
   colour = "firebrick",
   linetype = "dashed"
 )
g5 <- ggplot(parameter_df[ind_not_na,], aes (log10(mean_scran), DM)) +
 plot_params +
 xlab("scran means (log10)") +
 ylab("scran DM")
g6 <- ggplot(parameter_df[ind_not_na,], aes (log10(mu), epsilon)) +
 plot_params +
 xlab("BASiCS means (log10)") +
 ylab("BASiCS residual\nover-dispersion")
(g1 + g2 + g3) / (g4 + g5 + g6) +
 plot_annotation(tag_levels = "A") & theme(plot.tag = element_text(size = 15))


### HVG/LVG detection using BASiCS

In *BASiCS*, the functions BASiCS_DetectHVG and BASiCS_DetectLVG can be used to identify genes with substantially high (HVG) or low (LVG) transcriptional variability within a population of cells. If the input BASiCS_Chain object was generated by BASiCS_MCMC with Regression = TRUE (recommended setting), this analysis is based on the posterior distribution obtained for gene-specific residual over-dispersion parameters $\varepsilon_{i}$ (alternatively, the approach introduced by ^23^ can be used). HVGs are marked as those for which $\varepsilon_{i}$ exceeds a pre-defined threshold with high probability, where the probability cut-off is chosen to match a given expected false discovery rate (EFDR; by default the target EFDR is set to 10%). ^54^ A similar approach is implemented for LVG detection, but based on whether $\varepsilon_{i}$ is below a pre-specified threshold EpsilonThreshold. For example, if the threshold for $\varepsilon_{i}$ is equal to $\log\left(2\right)$ (the default), HVGs would be those genes for which the over-dispersion is estimated to be at least two times higher than would be expected given the inferred mean expression level, while LVGs would be those genes for which the residual over-dispersion is at least two times lower than would be expected on the same basis. In some circumstances, it may be of interest to rank genes and to select those with the highest or the lowest residual over-dispersion, which can be performed using the PercentileThreshold parameter; see help("BASiCS_DetectVG") for more details.

## Highly variable genes
hvg <- BASiCS_DetectHVG(chain_naive, EpsilonThreshold = log(2))
## Lowly variable genes
lvg <- BASiCS_DetectLVG(chain_naive, EpsilonThreshold = -log(2))
vg_table <- merge(
 as.data.frame(hvg, Filter = FALSE),
 as.data.frame(lvg, Filter = FALSE),
 all = TRUE
)
## mark genes as highly variable, lowly variable, or not either.
vg_table$VG <- "Not HVG or LVG"
vg_table$VG [vg_table$HVG] <- "HVG"
vg_table$VG [vg_table$LVG] <- "LVG"
ggplot (vg_table) +
 aes(log10(Mu), Epsilon, colour = VG) +
 geom_point() +
 geom_hline(yintercept = 0, lty = 2) +
 labs(
   x = "BASiCS means (log10)",
   y = "BASiCS residual\nover-dispersion"
 ) +
 scale_colour_manual(name = NULL,
   values = c(
     "HVG" = "firebrick",
     "LVG" = "dodgerblue",
     "Not HVG or LVG" = "grey80"
   ),
   na.value = "grey80"
 )


**Figure 9.**
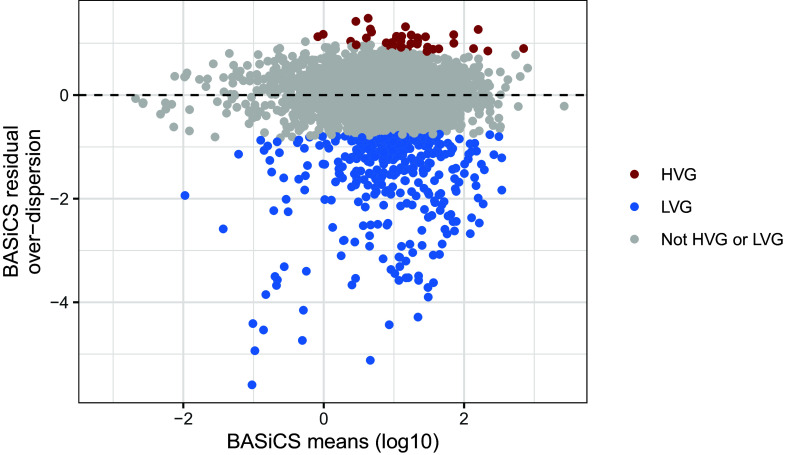
Highly-variable gene (HVG) and lowly-variable gene (LVG) detection using BASiCS. For each gene, BASiCS posterior estimates (posterior medians) associated to mean expression and residual over-dispersion parameters are plotted. Genes are coloured according to HVG/LVG status. Genes that are not expressed in at least two cells are excluded.

**Figure 10.**
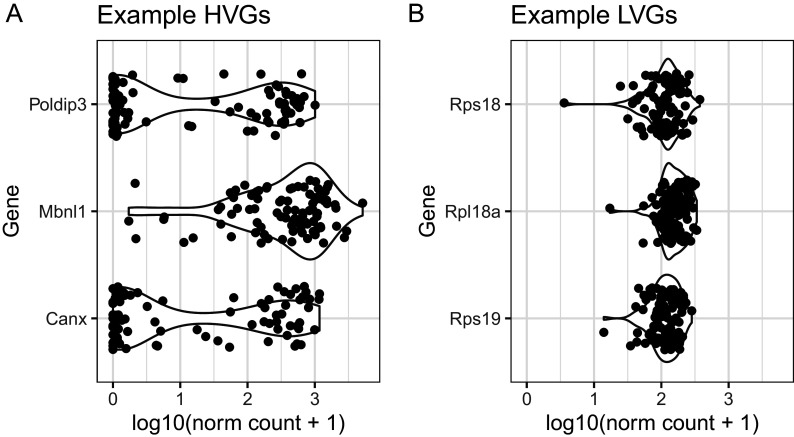
BASiCS denoised counts for example highly-variable gene (HVG) and lowly-variable gene (LVG) with similar overall levels of expression.

For the naive CD4 ^+^ T cell data, we obtained *41* HVG and *380* LVG. As shown in Figure 9, these genes are distributed across a wide range of mean expression values. As an illustration, Figure 10 shows the distribution of normalised expression values for selected HVG and LVG, focusing on examples with similar mean expression levels. As expected, HVG tend to exhibit a wider distribution and potentially bimodal distribution ( Figure 10A). Instead, LVG tend to have more narrow and unimodal distributions ( Figure 10B).

library("reshape2")
## Obtain normalised expression values
dc_naive <- BASiCS_DenoisedCounts(sce_naive, chain_naive)
vg_table <- merge(
 as.data.frame(lvg, Filter = FALSE),
 as.data.frame(hvg, Filter = FALSE),
 by = c("GeneName", "GeneIndex", "Mu", "Delta", "Epsilon"),
 suffixes = c("LVG", "HVG")
)
vg_table <- merge(
 vg_table,
 genenames,
 by.x = "GeneName", by.y = "ensembl_gene_id",
 sort = FALSE
)
## Select HVG/LVG genes with similar mean expression values
low_exp <- 2
up_exp <- 3
is_mid_exp <- log10(vg_table$Mu) > low_exp & log10(vg_table$Mu) < up_exp
hvg_table <- vg_table[which(is_mid_exp & vg_table$HVG),]
lvg_table <- vg_table[which(is_mid_exp & vg_table$LVG),]
## Order by epsilon and select top 3 HVG and LVG within the genes selected above
top_hvg <- order(hvg_table$Epsilon, decreasing = TRUE)[1:3]
top_lvg <- order(lvg_table$Epsilon, decreasing = FALSE)[1:3]
hvg_counts <- log10(t(dc_naive[hvg_table$GeneName[top_hvg],]) + 1)
lvg_counts <- log10(t(dc_naive[lvg_table$GeneName[top_lvg],]) + 1)
## Add genenames
colnames(hvg_counts) <- hvg_table$external_gene_name[top_hvg]
colnames(lvg_counts) <- lvg_table$external_gene_name[top_lvg]
plot_params <- list(
 geom_violin(na.rm = TRUE),
 coord_flip(),
 ylim(-0.05, max(log10(dc_naive + 1))),
 geom_jitter(position = position_jitter(0.3)),
 ylab("log10(norm count + 1)"),
 xlab("Gene")
)
plot_hvg <- ggplot(melt(hvg_counts), aes(x = Var2, y = value)) +
 plot_params + ggtitle("Example HVGs")
plot_lvg <- ggplot(melt(lvg_counts), aes(x = Var2, y = value)) +
 plot_params + ggtitle("Example LVGs")
plot_hvg + plot_lvg + plot_annotation(tag_levels = "A")


### Differential mean and variability testing using BASiCS

This section highlights the use of *BASiCS* to perform differential expression tests for mean and variability between different pre-specified populations of cells and experimental conditions. Here, we compare the naive CD4 ^+^ T cells, analysed in the previous section, to activated CD4 ^+^ T cells analysed in the same study. ^7^ Naive CD4 ^+^ T cells were activated for three hours using plate-bound CD3e and CD28 antibodies. T cell activation is linked to strong transcriptional shifts and the up-regulation of lineage specific marker genes, such as Tbx21 and Gata1. ^55^^,^^56^ To generate this data, the authors did not add cytokines, which are needed for T cell differentiation. ^57^ Therefore, any heterogeneity in the activated cell population does not arise from cells residing in different lineage-specific differentiation states.

Differential expression testing is performed via the BASiCS_TestDE function. The main input parameters are

• Chain1 and Chain2: two BASiCS_Chain objects created via the BASiCS_MCMC function. Each object corresponds to a different pre-specified group of cells.
• EpsilonM and EpsilonR: introduce a minimum effect size (in a $\log_{2}$ fold change scale) for the detection of changes in mean or residual over-dispersion, respectively. This enables us to discard small expression changes that are less biologically meaningful. By default, we set these thresholds to be equivalent to a 50% change between the groups. However, different thresholds may be required depending on the context. For example, if most genes show strong differences in mean expression, it can be beneficial to increase the value of EpsilonM to focus on strong changes in mean expression.
• EFDR_M and EFDR_R: define the target EFDR to calibrate the decision rule associated to changes in mean or residual over-dispersion, respectively. Default: 10%.
• MinESS: ESS threshold, below which genes will be excluded from the differential expression tests. This is used to increase the robustness of the results, excludes genes for which the sampler explored the parameter space less efficiently (see *MCMC diagnostics* Section). Default: MinESS = 100.


## Perform differential testing
test_de <- BASiCS_TestDE(
 Chain1 = chain_naive,
 Chain2 = chain_active,
 GroupLabel1 = "Naive",
 GroupLabel2 = "Active",
 EFDR_M = 0.1,
 EFDR_R = 0.1,
 MinESS = 100,
 Plot = FALSE,
 PlotOffset = FALSE
)
table_de_mean <- as.data.frame(
 test_de,
 Parameter = "Mean",
 Filter = FALSE
)
table_de_resdisp <- as.data.frame(
 test_de,
 Parameter = "ResDisp",
 Filter = FALSE
)


After running the test, it is important to visualise the results to facilitate interpretation and to identify systematic patterns among differentially expressed genes. It may also be useful to perform functional enrichment analysis to identify biologically meaningful patterns among these genes. For example, this could be performed using the Bioconductor package *goseq.* ^58^ We do not perform this here, but a relevant workflow is described by Maksimovic *et al*. ^59^

**Figure 11.**
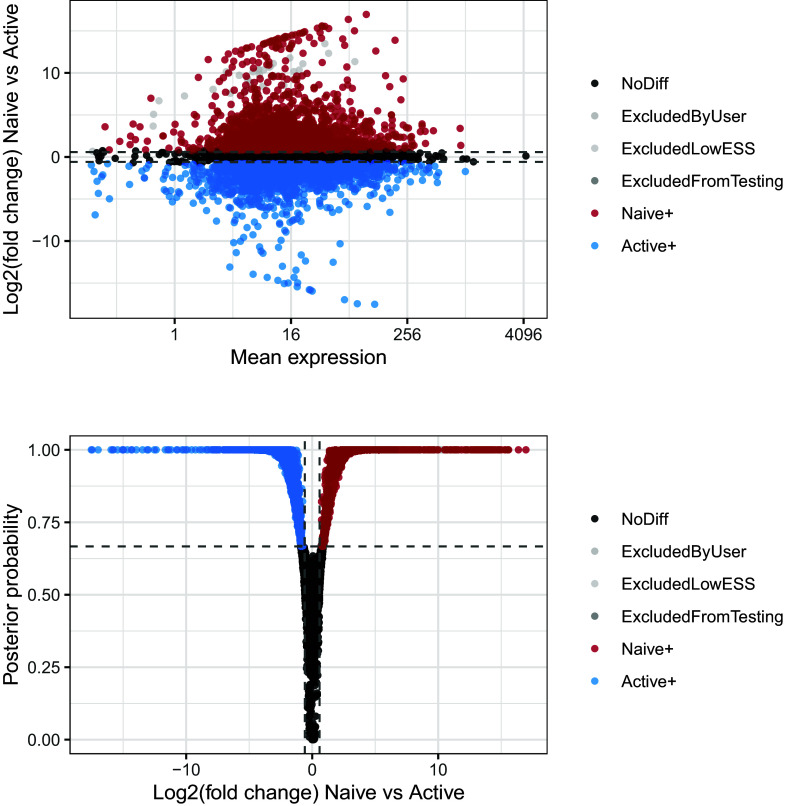
Upper panel presents the mean-difference plot associated to the differential mean expression test between naive and active cells. Log-fold changes of average expression in naive cells relative to active cells are plotted against average expression estimates combined across both groups of cells. Bottom panel presents the volcano plot associated to the same test. Log-fold changes of average expression in naive cells relative to active cells are plotted against their associated tail posterior probabilities. Colour indicates the differential expression status for each gene, including a label to identify genes that were excluded from differential expression test due to low effective sample size.

We first focus on the differential mean expression test. MA-plots (log fold change M versus mean average A) and volcano plots (posterior probability versus log fold change) are popular graphical summaries in this context, and are presented in Figure 11. These can be useful in ensuring that suitable magnitude and confidence thresholds have been chosen. In this instance, it is clear that a large number of genes are differentially expressed between the two conditions, and the selected probability threshold is suitable.

p1 <- BASiCS_PlotDE(test_de, Parameters = "Mean", Plots = "MA")
p2 <- BASiCS_PlotDE(test_de, Parameters = "Mean", Plots = "Volcano")
p1 / p2


When interpreting the results of differential expression tests, it is useful to visualise expression patterns for differentially expressed genes in order to appraise the significance of the results and guide interpretation. For this purpose, we obtain normalised expression values for each group of cells after correcting for global changes in overall expression between the groups, i.e. a global offset that leads to uniformly higher expression across genes within one group of cells (such step is also internally done by BASiCS_TestDE). Among other causes, the latter can be due to overall differences in mRNA content between the groups.

## Calculate a global offset between the groups
offset <- BASiCS_CorrectOffset(chain_naive, chain_active)
offset$Offset

## [1] 0.5987445

## Get offset corrected chain
chain_naive <- offset$Chain
## Obtain normalised counts within each group of cells
dc_naive <- BASiCS_DenoisedCounts(sce_naive, chain_naive, WithSpikes = FALSE)
dc_active <- BASiCS_DenoisedCounts(sce_active, chain_active, WithSpikes = FALSE)


**Figure 12.**
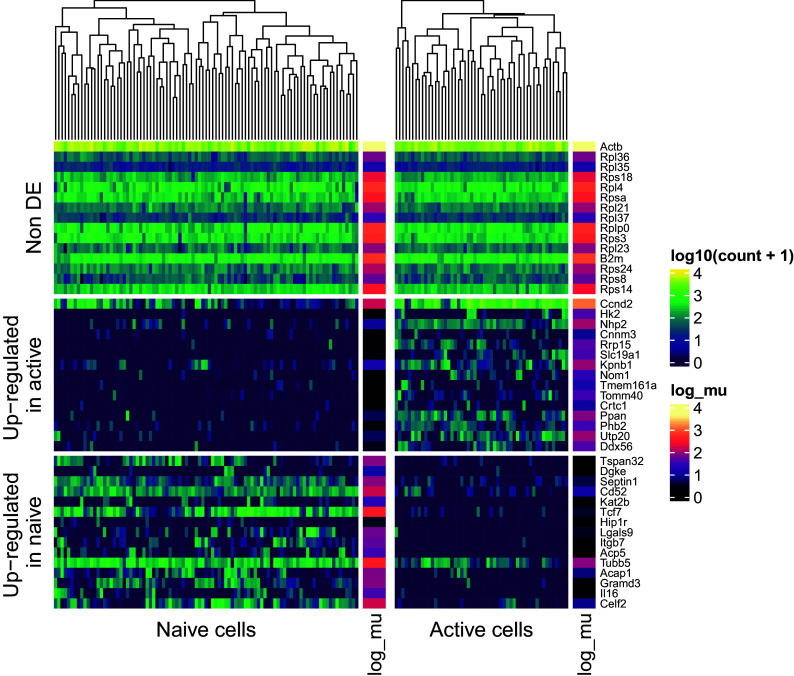
Heatmap displays normalised expression values (log _1_0( *x* + 1) scale) for naive and active cells. Genes are stratified according to their differential expression status (non differentially expressed; upregulated in naive or active cells). For each group, 15 example genes are shown. These were selected according to the ranking of their associated tail posterior probabilities associated to the differential mean expression test. Colour indicates expression level; colour bars on the right of heatmap segments indicate the inferred mean expression level (in log scale) for each gene in each population.

To visualise expression patterns for multiple genes at once, we use the Bioconductor package *ComplexHeatmap* ^60^ package, grouping genes according to the result of the differential mean expression test (i.e. up-regulated in naive/active cells or non differentially expressed; see Figure 12). For example, among the non DE group, we observe several genes encoding ribosomal proteins (e.g. *Rps14*). Genes in this family have been previously observed to have stable expression across a wide range of scRNAseq datasets in mouse and human. ^8^ Such visualisations may aid in the interpretation of such stable or “housekeeping” genes, as well as genes which are up- or down-regulated in each population.

library("ComplexHeatmap")
library("circlize")
library("RColorBrewer")
## Add gene symbol to table
table_de_mean$Symbol <- genenames[table_de_mean$GeneName, 2]
## utility function for selecting genes
select_top_n <- function(table, counts, condition, n=15, decreasing=TRUE) {
 ind_condition <- table$ResultDiffMean == condition
 table <- table[ind_condition,]
 ind_diff <- order(table$ProbDiffMean, decreasing = decreasing)[1:n]
 genes <- table$GeneName[ind_diff]
 counts[genes,]
}
## Active & naive count matrices for genes up-regulated in active cells
act_counts_act <- select_top_n(table_de_mean, dc_active, "Active+")
nai_counts_act <- select_top_n(table_de_mean, dc_naive, "Active+")
## Active & naive count matrices for genes up-regulated in naive cells
act_counts_nai <- select_top_n(table_de_mean, dc_active, "Naive+")
nai_counts_nai <- select_top_n(table_de_mean, dc_naive, "Naive+")
## Active & naive count matrices for genes not differentially expressed
act_counts_nde <- select_top_n(table_de_mean, dc_active, "NoDiff",
 decreasing = FALSE)
nai_counts_nde <- select_top_n(table_de_mean, dc_naive, "NoDiff",
 decreasing = FALSE)
## Combine count matrices by cell type
counts_active <- rbind(act_counts_act, act_counts_nai, act_counts_nde)
counts_naive <- rbind(nai_counts_act, nai_counts_nai, nai_counts_nde)
## split heatmaps by gene category
split <- data.frame(
 Upregulated = c(
   rep("Up-regulated \nin active", nrow(act_counts_act)),
   rep("Up-regulated \nin naive", nrow(act_counts_nai)),
   rep("Non DE", nrow(act_counts_nde))
 )
)
syms <- genenames[rownames(counts_active), 2]
fontsize <- 7
## Color palettes using circlize
col <- colorRamp2(
 breaks = seq(0,
   log10(max(c(counts_naive, counts_active) + 1)),
   length.out = 20
 ),
 colors = viridis(20)
)
## Subset table of DE results to extract mean estimates
match_order <- match(rownames(counts_naive), table_de_mean$GeneName)
table_de_selected <- table_de_mean[match_order,]
## Color palette for mu annotation
log_mu_naive <- log10(table_de_selected$Mean1)
log_mu_active <- log10(table_de_selected$Mean2)
mu_col <- colorRamp2(
 breaks = seq(0, max(c(log_mu_naive, log_mu_active)), length.out = 20),
 colors = viridis(20, option = "A", direction = 1)
)
Heatmap(
 log10(counts_naive + 1),
 row_labels = syms,
 row_names_gp = gpar(fontsize = fontsize),
 name = "log10(count + 1)",
 column_dend_height = unit(0.2, "npc"),
 column_title_side = "bottom",
 column_title = "Naive cells",
 show_column_names = FALSE,
 cluster_rows = FALSE,
 split = split,
 right_annotation = rowAnnotation(
   log_mu = log_mu_naive,
   col = list(log_mu = mu_col)
 ),
 col = col) +
 Heatmap(
   log10(counts_active + 1),
   row_labels = syms,
   column_dend_height = unit(0.2, "npc"),
   row_names_gp = gpar(fontsize = fontsize),
   column_title = "Active cells",
   column_title_side = "bottom",
   show_column_names = FALSE,
   split = split,
   show_heatmap_legend = FALSE,
   right_annotation = rowAnnotation(
       log_mu = log_mu_active,
       col = list(log_mu = mu_col)
   ),
   cluster_rows = FALSE,
   col = col)


While several computational tools exist to perform differential mean expression analysis using scRNAseq data, ^37^ the key focus of *BASiCS* is to perform differential variability testing: identifying changes in transcriptional variability between the groups of cells. To avoid the confounding between mean and over-dispersion, we recommend to use residual over-dispersion parameters $\varepsilon_{i}$ as input to this analysis.

**Figure 13.**
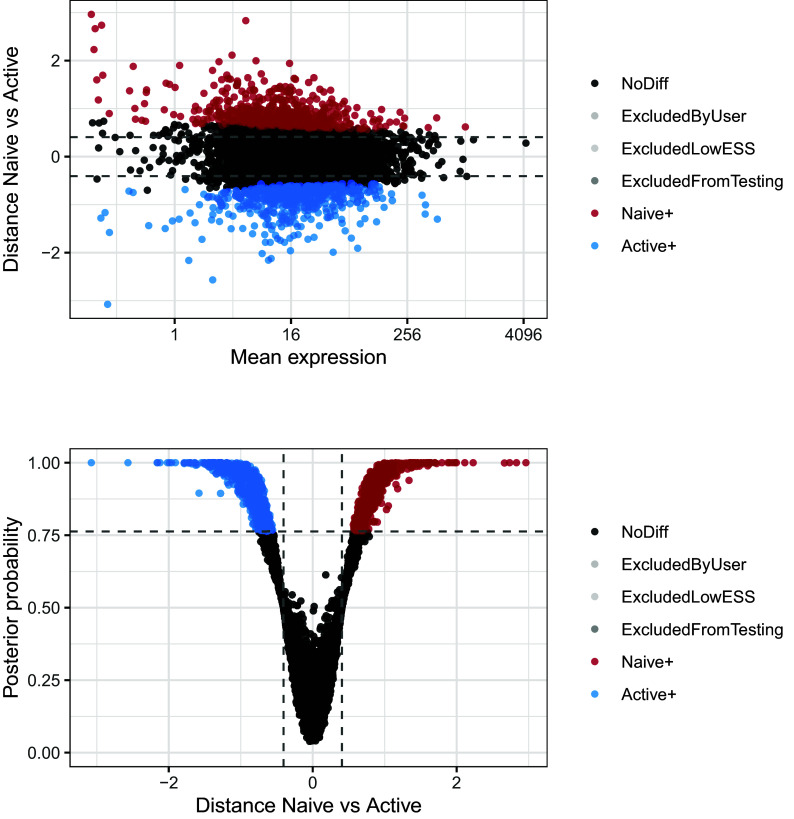
Upper panel presents the mean-difference plot associated to the differential residual over-dispersion test between naive and active cells. Differences of residual over-dispersion in naive cells relative to active cells are plotted against average expression estimates combined across both groups of cells. Bottom panel presents the volcano plot associated to the same test. Differences of residual over-dispersion in naive cells relative to active cells are plotted against their associated tail posterior probabilities. Colour indicates the differential expression status for each gene, including a label to identify genes that were excluded from differential expression test due to low effective sample size.

We can now visualise the changes in residual over-dispersion between naive and activated CD4 ^+^ T cells in the form of a MA-plot ( Figure 13). In this visualisation, the difference between the posterior medians of the residual over-dispersion parameters $\varepsilon_{i}$ are shown on the y-axis. Epsilon values for genes that are not expressed in at least two cells per condition are marked as NA and are therefore not displayed.

p1 <- BASiCS_PlotDE(test_de, Parameters = "ResDisp", Plots = "MA")
p2 <- BASiCS_PlotDE(test_de, Parameters = "ResDisp", Plots = "Volcano")
p1 / p2


**Figure 14.**
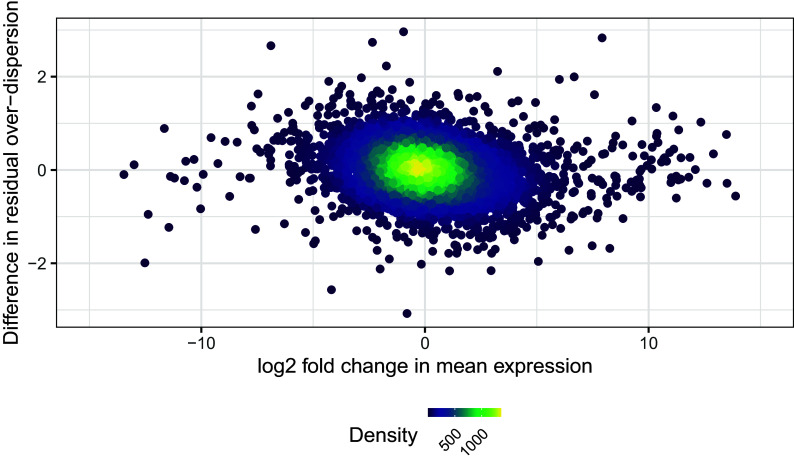
Difference in residual over-dispersion against log2 fold change in mean expression.

While one could focus on the sets of gene that show significant changes in residual over-dispersion, here we want to highlight how to analyse changes in mean expression in parallel to changes in variability. For this, we will first combine the results of the differential mean expression and the differential residual over-dispersion test. These are independent analyses, given that changes in residual over-dispersion are not confounded by changes in mean expression, as shown in Figure 14.

## combine results of differential mean and residual over-dispersion tests
table_de_combined <- merge(table_de_mean, table_de_resdisp)
## merge with some summary statistics about genes
gene_tests <- data.frame(
 "GeneName" = rownames(dc_naive),
 "ExpPropNaive" = rowMeans(dc_naive > 0),
 "ExpPropActive" = rowMeans(dc_active > 0),
 "nExpPropNaive" = rowSums(dc_naive > 0),
 "nExpPropActive" = rowSums(dc_active > 0)
)
table_combined_genes <- merge(table_de_combined, gene_tests)
## create list of generic plot parameters to use across a few plots
plot_params <- list(
 geom_pointdensity(),
 scale_colour_viridis(name = "Density"),
 theme(
   # text = element_text(size = rel(3)),
   legend.position = "bottom",
   legend.text = element_text(angle = 45, size = 8, hjust = 1, vjust = 1),
   legend.key.size = unit(0.018, "npc")
 )
)
## plot log2FC against difference of residual over-dispersion
ggplot(table_de_combined) +
 aes(MeanLog2FC, ResDispDistance) +
 plot_params +
 xlim(-15, 15) +
 labs(
   x = "log2 fold change in mean expression",
   y = "Difference in residual over-dispersion"
 )


**Figure 15.**
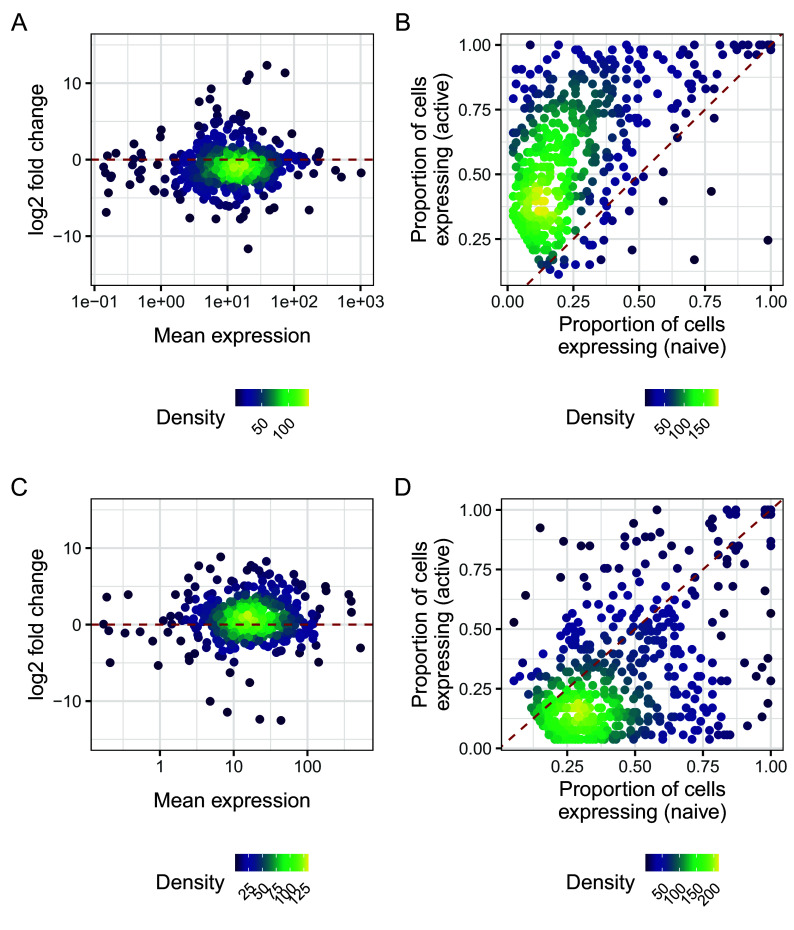
A, C: log2 change in expression against against log mean expression for genes with higher residual over-dispersion in naive (A) cells and active (D) cells. B, D: Proportion of expressed cells for genes, with higher residual over-dispersion in naive cells (B) and active (D) cells. Dashed red lines in panels A and C represent a log fold change of zero, meaning no change in average expression. Dashed red lines in panels B and D represent the line described by y=x, representing equal detection levels in both populations.

While genes with significant changes in residual over-dispersion often have similar levels of mean expression, as seen in Figure 15A and C, they may have a different proportion of zero counts in the two cell populations. Figure 15B and D show that many genes with higher residual over-dispersion in naive cells have a lower proportion of zeros in active cells, and vice versa.

g1 <- ggplot(
   table_combined_genes[table_combined_genes$ResultDiffResDisp == "Naive+",]
 ) +
 aes(MeanOverall, MeanLog2FC) +
 plot_params +
 ylim(-15, 15) +
 scale_x_log10() +
 labs(x = "Mean expression", y = "log2 fold change") +
 geom_hline(
   yintercept = 0, colour = "firebrick", linetype = "dashed"
 )
g2 <- ggplot(
   table_combined_genes[table_combined_genes$ResultDiffResDisp == "Active+",]
 ) +
 aes(MeanOverall, MeanLog2FC) +
 plot_params +
 ylim(-15, 15) +
 scale_x_log10() +
 labs(x = "Mean expression", y = "log2 fold change") +
 geom_hline(
   yintercept = 0, colour = "firebrick", linetype = "dashed"
 )
g3 <- ggplot(
   table_combined_genes[table_combined_genes$ResultDiffResDisp == "Naive+",]
 ) +
 aes(x = ExpPropNaive, y = ExpPropActive) +
 plot_params +
 labs(
   x = "Proportion of cells\nexpressing (naive)",
   y = "Proportion of cells\nexpressing (active)"
 ) +
 geom_abline(
   slope = 1, intercept = 0, colour = "firebrick", linetype = "dashed"
 )
g4 <- ggplot(
   table_combined_genes[table_combined_genes$ResultDiffResDisp == "Active+",]
 ) +
 aes(x = ExpPropNaive, y = ExpPropActive) +
 plot_params +
 labs(
   x = "Proportion of cells\nexpressing (naive)",
   y = "Proportion of cells\nexpressing (active)"
 ) +
 geom_abline(
   slope = 1, intercept = 0, colour = "firebrick", linetype = "dashed"
 )
(g1 + g3) / (g2 + g4) + plot_annotation(tag_levels = "A")


Similarly to analysis of differential expression, it is useful to visualise the results of differential variability tests in order to appraise the quality of the results, and to identify systematic patterns among the genes identified. One useful way to do this is by examining the normalised counts on a gene-by-gene basis. Figure 16 shows denoised counts for genes with significant differences in residual over-dispersion, with each panel showing a different type of expression pattern. Exploration of such patterns is important component of any analysis of differential variability, and should be undertaken with care. Figure 16B and 16D show genes with differing levels of detection in both populations, as well as higher levels of residual over-dispersion in naive or active cells (B and D, respectively). Thus, these genes may represent those with a more bursty expression pattern in one of the cell population. They may also represent genes that are markers of extrinsic variability, for example cell sub-populations that differ in abundance between the cell populations in question. In contrast, Figure 16A and 16C show genes with similar levels of detection in both populations, as well as higher levels of residual over-dispersion in naive or active cells (A and C, respectively). These cases are likely driven by more tight regulation, rather than transcriptional burst or sub-population structure.

## Use the tidyr package to reshape data into a "long" format for ggplot2
library("tidyr")
## Utility function that plots logcounts of a set of genes defined by "ind_vg"
plot_vg <- function(ind_vg) {
 table_combined_genes_vg <- table_combined_genes[ind_vg,]
 ## If more than 4 genes,
 ## pick top 4 ranked by differences in residual over-dispersion
 if (nrow(table_combined_genes_vg) > 4) {
   table_combined_genes_vg <- table_combined_genes_vg[
     order (abs (table_combined_genes_vg$ResDispDistance), decreasing = TRUE),]
   table_combined_genes_vg <- table_combined_genes_vg[1:4,]
 }
 var_genes <- table_combined_genes_vg$GeneName
 var_naive <- dc_naive[var_genes, , drop = FALSE]
 var_active <- dc_active[var_genes, , drop = FALSE]
 var_df <- rbind(
   data.frame(t(var_naive), Group = "Naive"),
   data.frame(t(var_active), Group = "Active")
 )
 colnames (var_df)[-ncol(var_df)] <- genenames[var_genes, "external_gene_name"]
 var_long_df <- pivot_longer(var_df, cols = colnames(var_df)[-ncol(var_df)])
ggplot (var_long_df, aes(x = name, y = value + 1, colour = Group)) +
 geom_violin(width = 0.8, position = position_dodge(width = 0.8)) +
 geom_point(
   position = position_jitterdodge(jitter.width = 0.2, dodge.width = 0.8),
   alpha = 0.5
 ) +
 scale_x_discrete(guide = guide_axis(angle = 45)) +
 scale_y_log10() +
 labs(x = "Gene", y = "Denoised count + 1") +
 scale_colour_brewer(palette = "Set1")
}
## genes more variable in naive &
## not differentially expressed &
## with similar levels of non-zero expression &
## expressed in at least 50% of cells within each group
## ie, genes that are probably unimodal but with higher variance in naive cells
ind_vg <- table_combined_genes$ResultDiffResDisp %in% c("Naive+") &
 table_combined_genes$ResultDiffMean %in% c("NoDiff") &
 abs(
   table_combined_genes$ExpPropNaive - table_combined_genes$ExpPropActive
 ) < 0.05 &
 pmin(
   table_combined_genes$ExpPropNaive, table_combined_genes$ExpPropActive
 ) > 0.75
g1 <- plot_vg(ind_vg) +
   theme(legend.position = "none")
## genes more variable in naive &
## not differentially expressed &
## with different levels of non-zero expression &
## expressed in at least 50% of cells within one or more of the groups
## i.e., genes with more bursting pattern of expression
ind_vg <- table_combined_genes$ResultDiffResDisp %in% c("Naive+") &
 table_combined_genes$ResultDiffMean %in% c("NoDiff") &
 abs(
   table_combined_genes$ExpPropNaive - table_combined_genes$ExpPropActive
 ) > 0.40 &
 pmax(
   table_combined_genes$ExpPropNaive, table_combined_genes$ExpPropActive
 ) > 0.75
g2 <- plot_vg(ind_vg)
## genes more variable in Active &
## not differentially expressed &
## with similar levels of non-zero expression &
## expressed in at least 50% of cells within each group
## ie, genes that are probably unimodal but with higher variance
ind_vg <- table_combined_genes$ResultDiffResDisp %in% c("Active+") &
 table_combined_genes$ResultDiffMean %in% c("NoDiff") &
 abs(
   table_combined_genes$ExpPropNaive - table_combined_genes$ExpPropActive
 ) < 0.05 &
 pmin(
   table_combined_genes$ExpPropNaive, table_combined_genes$ExpPropActive
 ) > 0.75
g3 <- plot_vg(ind_vg) +
   theme(legend.position = "none")
## genes more variable in Active &
## not differentially expressed &
## with different levels of dropout &
## expressed in at least 50% of cells within one or more of the groups
## i.e., genes with more bursting pattern of expression
ind_vg <- table_combined_genes$ResultDiffResDisp %in% c("Active+") &
 table_combined_genes$ResultDiffMean %in% c("NoDiff") &
 abs(
   table_combined_genes$ExpPropNaive - table_combined_genes$ExpPropActive
 ) > 0.20 &
 pmax(
   table_combined_genes$ExpPropNaive, table_combined_genes$ExpPropActive
 ) > 0.50
g4 <- plot_vg(ind_vg)
(g1 + g2) / (g3 + g4) + plot_annotation(tag_levels = "A")


**Figure 16.**
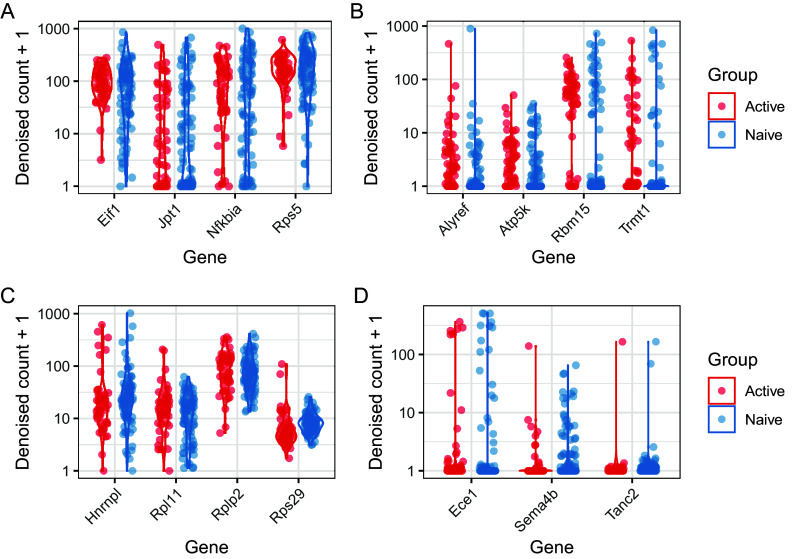
Violin plots of denoised counts. A: Four genes with higher residual over-dispersion in naive cells, and similar levels of detection in active and naive populations. B: Four genes with higher residual over-dispersion in naive cells and different levels of detection in naive and active cells. C: Four genes with higher residual over-dispersion in active cells and similar levels of detection in naive and active cells. D: Three genes with higher residual over-dispersion in active cells and different levels of detection in naive and active cells.

### Using BASiCS without spike-ins

*BASiCS*, when using spike-in molecules, uses spike-ins as a reference in order to aid normalisation, based on the assumption that the original quantity of spike-in molecules was approximately equal in each well. Eling *et al*. ^29^ introduced a novel method of inferring gene expression profiles using *BASiCS* without relying on spike-ins to quantify technical noise. This is useful for droplet-based scRNAseq protocols, given that it is not possible to ensure that each droplet contains a specified quantity of spike-in molecules. In this horizontal integration framework, technical variation is quantified using replication. ^61^ In the absence of true technical replicates, we assume that population-level characteristics of the cells are replicated using appropriate experimental design. This requires that cells from the same population have been randomly allocated to different batches. Given appropriate experimental design, *BASiCS* assumes that biological effects are shared across batches, while technical variation leads to spurious differences between cells in different batches.

Using *BASiCS* without spike-ins is very similar to using it with spike-ins. We will demonstrate using the naive cells. However, first, we must ensure that a BatchInfo field is present in the SingleCellExperiment used as input. In this case we use individual of origin as the batch vector.

set.seed(42)
chain_naive_nospikes <- BASiCS_MCMC(
 Data = sce_naive,
 PrintProgress = TRUE,
 N = 40000,
 Thin = 20,
 Burn = 20000,
 Regression = TRUE,
 PriorParam = prior_param_naive,
 Threads = 4,
 StoreChains = TRUE,
 StoreDir = "rds/",
 RunName = "naive_nospikes",
 WithSpikes = FALSE
)


As before, for convenience we provide a completed version of this chain at https://doi.org/10.5281/zenodo.5243265 .

if (!file.exists("rds/chain_naive_nospikes.Rds")) {
 download.file(
   paste0(chains_website, "/chain_naive_nospikes.Rds"),
   destfile = "rds/chain_naive_nospikes.Rds"
 )
}
chain_naive_nospikes <- readRDS("rds/chain_naive_nospikes.Rds")


The resulting BASiCS_Chain object produced using this horizontal integration framework is functionally similar to one produced using the vertical integration framework. It can be used in place of the BASiCS_Chain objects produced using the vertical integration approach, as described above.

### Comparison of parameter estimates with and without spike-ins

**Figure 17.**
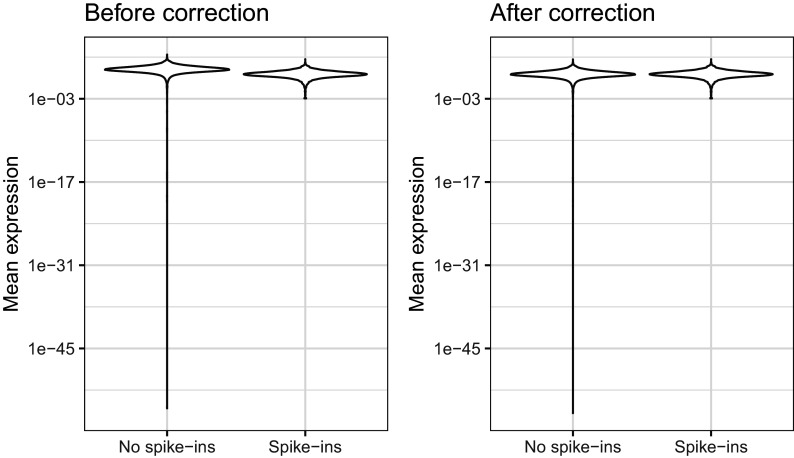
Distribution of mean expression values before and after correcting the global difference in scale.

Under the horizontal integration approach described above, the scale of mean expression parameters and global scaling factors is not jointly identifiable, in that a global shift in mean expression parameters could be exactly offset by an equivalent shift in cell-specific normalisation parameters. Therefore, the geometric mean of the mean expression parameters is fixed to a constant value. Relative expression level estimates are broadly consistent between the horizontal and vertical integration approaches; however there may be a global difference in mean expression estimates, as shown in Figure 17. It is important to remove this global scale offset before performing comparative analyses. This is performed by default in BASiCS_TestDE, but can be performed manually using BASiCS_CorrectOffset.

BASiCS_PlotOffset(chain_naive_nospikes, chain_naive,
 GroupLabel1 = "No spike-ins", GroupLabel2 = "Spike-ins",
 Type = "before-after")

offset <- BASiCS_CorrectOffset(chain_naive_nospikes, chain_naive)
chain_naive_nospikes_offset <- offset$Chain
chain_naive_nospikes_offset

## An object of class BASiCS_Chain
## 1000 MCMC samples.
## Dataset contains 5171 biological genes and 93 cells (2 batches).
## Object stored using BASiCS version: 2.2.1
## Parameters: mu delta s nu theta beta sigma2 epsilon RefFreq RBFLocations


**Figure 18.**
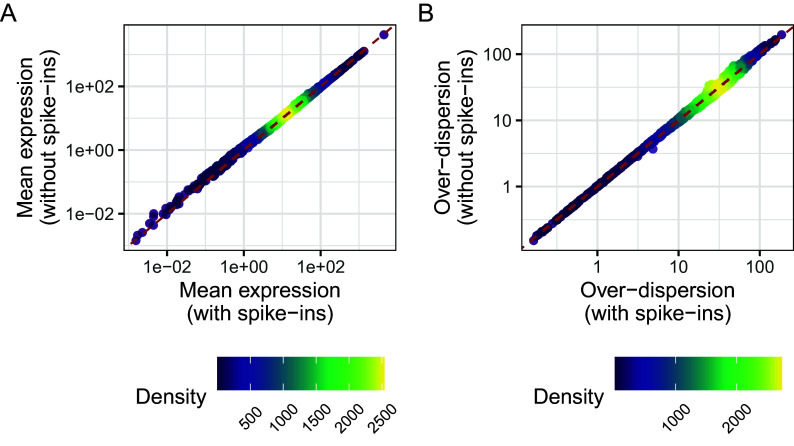
Comparison of point estimates using spike-ins, and the same parameters estimated without using spike-ins for mean expression (A) and over-dispersion (B). A dashed red line indicates the identity line, y=x . Genes with zero counts across all cells were excluded from the plot of mean expression parameters.

A number of genes have very low expression estimates in the naive population, due to the fact that they each have zero read counts across the entire naive population; we therefore remove these genes before making a comparison. Following removal of the global offset, the mean expression and over-dispersion estimates obtained from each method are directly comparable. As seen in Figure 18A and 18B, parameter point estimates from the two methods are highly correlated. There is a tail of non-expressed genes with very low mean expression level as inferred without spike-ins, comprising those genes with no measured expression across the entire population.

mu_spikes <- displayChainBASiCS(chain_naive)
mu_nospikes <- displayChainBASiCS(chain_naive_nospikes_offset)
# Remove genes with zero counts across all cells and calculate medians
ind_nonzero <- rowSums(counts(sce_naive)) != 0
mu_spikes <- colMedians(mu_spikes[, ind_nonzero])
mu_nospikes <- colMedians(mu_nospikes[, ind_nonzero])
g1 <- ggplot() +
 aes(mu_spikes, mu_nospikes) +
 geom_pointdensity(alpha = 0.7) +
 scale_colour_viridis(name = "Density") +
 scale_x_log10() +
 scale_y_log10() +
 geom_abline(
   colour = "firebrick",
   linetype = "dashed",
   slope = 1,
   intercept = 0
 ) +
 labs(
   x = "Mean expression\n(with spike-ins)",
   y = "Mean expression\n(without spike-ins)"
 ) +
 theme(
   legend.position = "bottom",
   legend.text = element_text(angle = 45, size = 8, hjust = 0.5, vjust = 0.5)
 )
delta_spikes <- displayChainBASiCS(chain_naive, Param = "delta")
delta_nospikes <- displayChainBASiCS(chain_naive_nospikes_offset, Param = "delta")
g2 <- ggplot() +
 aes(colMedians(delta_spikes), colMedians(delta_nospikes)) +
 geom_pointdensity(alpha = 0.7) +
 scale_colour_viridis(name = "Density") +
 scale_x_log10() +
 scale_y_log10() +
 geom_abline(
   colour = "firebrick",
   linetype = "dashed",
   slope = 1,
   intercept = 0
 ) +
 labs(
   x = "Over-dispersion\n(with spike-ins)",
   y = "Over-dispersion\n(without spike-ins)"
 ) +
 theme(
   legend.position = "bottom",
   legend.text = element_text(angle = 45, size = 8, hjust = 0.5, vjust = 0.5)
 )
g1 + g2 + plot_annotation(tag_levels = "A")


## Discussion

In this article, we have explored the research questions that *BASiCS* seeks to resolve — chiefly, robustly quantifying average and variability in expression in cell populations. We have outlined the appropriate quality control and data visualisation steps to apply when undertaking an analysis using *BASiCS* in order to ensure high quality input data. We have also outlined the steps needed to use *BASiCS* to quantify biological variability, identify highly variable genes, and normalise scRNAseq data from a single population. We have also provided a limited comparison of the results of these analyses using *BASiCS* and the result of similar analyses using *scran.* Furthermore, we have demonstrated functions within *BASiCS* that allow users to ensure the MCMC used in *BASiCS* has converged and produced adequate sample sizes. Finally, we have demonstrated the use of *BASiCS* to robustly identify differentially expressed genes, in terms of mean expression and in terms of biological variability.

Further challenges exist in analysing scRNAseq data. ^10^^,^^38^ For *BASiCS*, the primary challenge currently is computational efficiency. The number of cells profiled in scRNAseq experiments has scaled exponentially since the development of the technology. ^62^ Given that *BASiCS* requires computationally intensive MCMC sampling to estimate the posterior distribution, it becomes computationally intractable to analyse data from very large numbers of cells. We intend to update this workflow as the field evolves, and as we address the issues and challenges outlined here.

## Data availability

ArrayExpress: RNA-seq of coding RNA from single cells [Mus musculus (house mouse)]. Accession number E-MTAB-4888; https://identifiers.org/arrayexpress:E-MTAB-4888.

All data underlying the results are available as part of the article and no additional source data are required.

## Software availability

All software used in this workflow is available as part of Bioconductor 3.13 at: https://bioconductor.org/packages/3.13 .

The source code for BASiCS, along with facilities contributing and reporting bugs is available at: https://github.com/catavallejos/BASiCS/ .

The source code used for this manuscript is available at: https://github.com/VallejosGroup/BASiCSWorkflow/ , and as archived source code at time of publication: https://doi.org/10.5281/zenodo.5243265. ^32^

License: GPL-2.0

## Reproducibility

The following software versions were used throughout this workflow:

• **R version**: R version 4.1.1 (2021-08-10)
• **Bioconductor version**: 3.13
• **R packages**:
– BASiCS 2.4.0
– scran 1.20.1
– scater 1.20.1
  Version numbers for all remaining packages are available in the Session Info section.

A Docker image containing all software requirements is available at Docker hub. This image can be downloaded using the command docker pull alanocallaghan/bocker:0.2.0.

## Session Info



sessionInfo()
## R version 4.1.1 (2021-08-10)
## Platform: x86_64-pc-linux-gnu (64-bit)
## Running under: Ubuntu 20.04.3 LTS
##
## Matrix products: default
## BLAS/LAPACK: /usr/lib/x86_64-linux-gnu/openblas-pthread/libopenblasp-r0.3.8.so
##
## locale:
## [1] LC_CTYPE=en_US.UTF-8       LC_NUMERIC=C
## [3] LC_TIME=en_US.UTF-8       LC_COLLATE=en_US.UTF-8
## [5] LC_MONETARY=en_US.UTF-8     LC_MESSAGES=C
## [7] LC_PAPER=en_US.UTF-8       LC_NAME=C
## [9] LC_ADDRESS=C            LC_TELEPHONE=C
## [11] LC_MEASUREMENT=en_US.UTF-8   LC_IDENTIFICATION=C
##
## attached base packages:
## [1] grid     parallel stats4 stats graphics grDevices utils
## [8] datasets methods base
##
## other attached packages:
## [1] tidyr_1.1.4            RColorBrewer_1.1-2
## [3] circlize_0.4.13          ComplexHeatmap_2.8.0
## [5] reshape2_1.4.4          ggpointdensity_0.1.0
## [7] viridis_0.6.1           viridisLite_0.4.0
## [9] coda_0.19-4             patchwork_1.1.1
## [11] biomaRt_2.48.3          BASiCS_2.4.0
## [13] scran_1.20.1           scater_1.20.1
## [15] scuttle_1.2.1           SingleCellExperiment_1.14.1
## [17] SummarizedExperiment_1.22.0  Biobase_2.52.0
## [19] GenomicRanges_1.44.0      GenomeInfoDb_1.28.4
## [21] IRanges_2.26.0          S4Vectors_0.30.2
## [23] BiocGenerics_0.38.0        MatrixGenerics_1.4.3
## [25] matrixStats_0.61.0        ggplot2_3.3.5
## [27] knitr_1.36             BiocStyle_2.20.2
##
## loaded via a namespace (and not attached):
## [1] BiocFileCache_2.0.0       plyr_1.8.6
## [3] igraph_1.2.6           BiocParallel_1.26.2
## [5] usethis_2.0.1          digest_0.6.28
## [7] foreach_1.5.1          htmltools_0.5.2
## [9] magick_2.7.3           fansi_0.5.0
## [11] magrittr_2.0.1          memoise_2.0.0
## [13] ScaledMatrix_1.0.0         cluster_2.1.2
## [15] doParallel_1.0.16          limma_3.48.3
## [17] Biostrings_2.60.2          prettyunits_1.1.1
## [19] colorspace_2.0-2           blob_1.2.2
## [21] rappdirs_0.3.3           BiocWorkflowTools_1.18.0
## [23] xfun_0.26              dplyr_1.0.7
## [25] crayon_1.4.1            RCurl_1.98-1.5
## [27] hexbin_1.28.2            iterators_1.0.13
## [29] glue_1.4.2              gtable_0.3.0
## [31] zlibbioc_1.38.0           XVector_0.32.0
## [33] GetoptLong_1.0.5          DelayedArray_0.18.0
## [35] BiocSingular_1.8.1         shape_1.4.6
## [37] scales_1.1.1             DBI_1.1.1
## [39] edgeR_3.34.1            miniUI_0.1.1.1
## [41] Rcpp_1.0.7             xtable_1.8-4
## [43] progress_1.2.2           clue_0.3-59
## [45] dqrng_0.3.0            bit_4.0.4
## [47] rsvd_1.0.5              metapod_1.0.0
## [49] httr_1.4.2              ellipsis_0.3.2
## [51] pkgconfig_2.0.3           XML_3.99-0.8
## [53] farver_2.1.0            dbplyr_2.1.1
## [55] locfit_1.5-9.4           utf8_1.2.2
## [57] tidyselect_1.1.1          labeling_0.4.2
## [59] rlang_0.4.11            later_1.3.0
## [61] AnnotationDbi_1.54.1       munsell_0.5.0
## [63] tools_4.1.1             cachem_1.0.6
## [65] generics_0.1.0           RSQLite_2.2.8
## [67] evaluate_0.14           stringr_1.4.0
## [69] fastmap_1.1.0           yaml_2.2.1
## [71] bit64_4.0.5            fs_1.5.0
## [73] purrr_0.3.4            KEGGREST_1.32.0
## [75] sparseMatrixStats_1.4.2      mime_0.11
## [77] ggExtra_0.9              xml2_1.3.2
## [79] compiler_4.1.1           rstudioapi_0.13
## [81] beeswarm_0.4.0           filelock_1.0.2
## [83] curl_4.3.2              png_0.1-7
## [85] tibble_3.1.4             statmod_1.4.36
## [87] stringi_1.7.4             lattice_0.20-45
## [89] bluster_1.2.1             Matrix_1.3-4
## [91] vctrs_0.3.8              pillar_1.6.3
## [93] lifecycle_1.0.1            BiocManager_1.30.16
## [95] GlobalOptions_0.1.2         BiocNeighbors_1.10.0
## [97] cowplot_1.1.1            bitops_1.0-7
## [99] irlba_2.3.3             httpuv_1.6.3
## [101] R6_2.5.1               bookdown_0.24
## [103] promises_1.2.0.1           gridExtra_2.3
## [105] vipor_0.4.5             codetools_0.2-18
## [107] MASS_7.3-54             assertthat_0.2.1
## [109] rjson_0.2.20             withr_2.4.2
## [111] GenomeInfoDbData_1.2.6        hms_1.1.1
## [113] beachmat_2.8.1          rmarkdown_2.11
## [115] DelayedMatrixStats_1.14.3       Cairo_1.5-12.2
## [117] git2r_0.28.0            shiny_1.7.1
## [119] ggbeeswarm_0.6.0

