# BASiCS workflow: a step-by-step analysis of expression variability using single cell RNA sequencing data

**DOI:** 10.12688/f1000research.74416.2

**Published:** 2024-05-07

**Authors:** Alan O'Callaghan, Nils Eling, John C. Marioni, Catalina A. Vallejos

**Affiliations:** 1 MRC Human Genetics Unit, Institute of Genetics & Cancer, University of Edinburgh, Edinburgh, EH4 2XU, UK; 2 Institute for Molecular Health Sciences, ETH Zürich, Zürich, 8093, Switzerland; 3 Department of Quantitative Biomedicine, University of Zurich, Zürich, CH-8057, Switzerland; 4 Cancer Research UK Cambridge Institute, University of Cambridge, Cambridge, CB2 0RE, UK; 5 European Molecular Biology Laboratory, European Bioinformatics Institute, Cambridge, CB10 1SD, UK; 6 The Alan Turing Institute, The Alan Turing Institute, London, NW1 2DB, UK

**Keywords:** single-cell RNA sequencing, expression variability, transcriptional noise, differential expression testing, scRNAseq, Bayesian, bioinformatics, heterogeneity

## Abstract

Cell-to-cell gene expression variability is an inherent feature of complex biological systems, such as immunity and development. Single-cell RNA sequencing is a powerful tool to quantify this heterogeneity, but it is prone to strong technical noise. In this article, we describe a step-by-step computational workflow that uses the BASiCS Bioconductor package to robustly quantify expression variability within and between known groups of cells (such as experimental conditions or cell types). BASiCS uses an integrated framework for data normalisation, technical noise quantification and downstream analyses, propagating statistical uncertainty across these steps. Within a single seemingly homogeneous cell population, BASiCS can identify highly variable genes that exhibit strong heterogeneity as well as lowly variable genes with stable expression. BASiCS also uses a probabilistic decision rule to identify changes in expression variability between cell populations, whilst avoiding confounding effects related to differences in technical noise or in overall abundance. Using a publicly available dataset, we guide users through a complete pipeline that includes preliminary steps for quality control, as well as data exploration using the scater and scran Bioconductor packages. The workflow is accompanied by a Docker image that ensures the reproducibility of our results.

## Introduction

Single-cell RNA-sequencing (scRNA-seq) enables the study of genome-wide cell-to-cell transcriptional heterogeneity that is not captured by bulk experiments. ^1^^–^^3^ On the broadest level, this heterogeneity can reflect the presence of distinct cell subtypes or states. Alternatively, it can be due to gradual changes along biological processes, such as development and differentiation. Several clustering and pseudotime inference tools have been developed to capture these types of heterogeneity. ^4^^,^^5^ However, there is a limited availability of tools tailored to study more subtle variability within seemingly homogeneous cell populations. This variability can reflect deterministic or stochastic events that regulate gene expression and, among other settings, has been seen to increase prior to cell fate decisions ^6^ and during ageing. ^7^ Transcriptional variability has also been observed to differ from gene to gene and can be conserved across cell types and species. ^8^

Stochastic variability within a seemingly homogeneous cell population — often referred to as transcriptional *noise* — can arise from intrinsic and extrinsic sources. ^9^^,^^10^ Extrinsic noise refers to stochastic fluctuations induced by different dynamic cellular states (e.g. cell cycle, metabolism, intra/inter-cellular signalling). ^11^^–^^13^ In contrast, intrinsic noise arises from stochastic effects on biochemical processes such as transcription and translation. ^9^ Intrinsic noise can be modulated by genetic and epigenetic modifications (such as mutations, histone modifications, CpG island length and nucleosome positioning) ^14^^–^^16^ and usually occurs at the gene level. ^9^ Cell-to-cell gene expression variability estimates derived from scRNA-seq data capture a combination of these effects, as well as deterministic regulatory mechanisms. ^10^ Moreover, these variability estimates can also be inflated by the technical noise that is typically observed in scRNA-seq data. ^17^ This technical noise relates to systematic differences between cells that may be introduced by the data generating process (e.g., due to differences in dilution or sequencing depth). ^18^

Different strategies have been incorporated into scRNA-seq protocols to control or attenuate technical noise. For example, external RNA spike-in molecules (such as the set introduced by the External RNA Controls Consortium, ERCC ^19^) can be added to each cell’s lysate in a (theoretically) known fixed quantity. Spike-ins can assist quality control steps, ^20^ data normalisation ^18^ and can be used to infer technical noise. ^17^ Another strategy is to tag individual cDNA molecules using unique molecular identifiers (UMIs) before PCR amplification. ^21^ Reads that contain the same UMI can be collapsed into a single molecule count, attenuating technical variability associated to cell-to-cell differences in amplification and sequencing depth (these technical biases are not fully removed unless sequencing to saturation). ^18^ However, despite the benefits associated to the use of spike-ins and UMIs, these are not available for all scRNA-seq protocols. ^22^ In particular, spike-ins are of limited use in droplet-based protocols, as spike-ins can only be added to the reagent mixture in a known concentration, and the exact quantity in each droplet necessarily remains unknown. ^23^

The Bioconductor package *BASiCS* implements a Bayesian hierarchical framework that accounts for both technical and biological sources of noise in scRNA-seq datasets. ^24^^–^^26^ The model was initially motivated by supervised experimental designs in which experimental conditions correspond to groups of cells defined *a priori* (e.g. selected cell types obtained through FACS sorting). However, the approach can also be used in cases where the groups of cells of interest are computationally identified through clustering. In such cases, the model does not currently address issues associated to *post-selection inference*, where the same data is analysed twice: first to perform clustering and then to compare expression profiles between the clusters ^27^; this is an inherent limitation of most differential expression tools.

*BASiCS* jointly performs data normalisation, technical noise quantification and downstream analyses, whilst propagating statistical uncertainty across these steps. These features are implemented within a probabilistic model that builds upon a negative binomial framework, a widely used distribution in the context of bulk and scRNA-seq experiments. ^28^^–^^31^

The negative binomial distribution is commonly used to model count data when the observed variability differs from what can be captured by a simpler Poisson model — this is typically referred to as over-dispersion. Critically, *BASiCS* enables the quantification of transcriptional variability within a population of cells, while accounting for the overall mean-variance relationship that typically arises in scRNA-seq data. ^26^ The latter enables the quantification of a gene-level *residual over-dispersion* as a measure of transcriptional noise that is not confounded by differences in gene expression. Furthermore, when available, *BASiCS* can also leverage extrinsic spike-in molecules to aid data normalisation. A previous study has shown that *BASiCS* , when used as a generative model, is well-tuned to capture and recapitulate the main properties of typical scRNAseq data using posterior predictive checks. ^32^

This article complements existing scRNA-seq workflows based on the Bioconductor ecosystem (e.g. Refs. 33, 34), providing a detailed framework for transcriptional variability analyses using *BASiCS* . We describe a step-by-step workflow that uses *scater* ^20^ and *scran* ^33^ to perform quality control (QC) as well as initial exploratory analyses. Our analysis pipeline includes practical guidance to assess the convergence of the Markov Chain Monte Carlo (MCMC) algorithm that is used to infer model parameters in *BASiCS* , as well as recommendations to interpret and post-process the model outputs. Finally, through a case study in the context of mouse immune cells, we illustrate how *BASiCS* can identify highly and lowly variable genes within a cell population, as well as to compare expression profiles between experimental conditions or cell types.

All source code used to generate the results presented in this article is available in Github and Zenodo. ^26^ To ensure the reproducibility of this workflow, the analysis environment and all software dependencies are provided as a Docker image. ^35^ The image can be obtained from Docker Hub, at https://hub.docker.com/r/alanocallaghan/basicsworkflow2020-docker.

## Methods

### Implementation

The *BASiCS* Bioconductor package uses a Bayesian hierarchical model to simultaneously perform data normalisation, technical noise quantification and downstream analyses ^24^^–^^26^ within a cell population or populations under study. In this context, cell populations could correspond to groups set a priori by the experimental design (e.g. naive or stimulated CD4+ T cells in Ref. 7), or to groups of cells that were computationally identified through clustering. Moreover, instead of modelling expression patterns separately for each gene, *BASiCS* shares information between all genes to robustly quantify transcriptional variability. For example, as described (see Software availability section), pooling information across genes with similar mean expression levels helps to obtain more reliable transcriptional variability estimates. The latter is particularly helpful for lowly expressed genes and sparse datasets, where less information is available.

**Figure 1.**
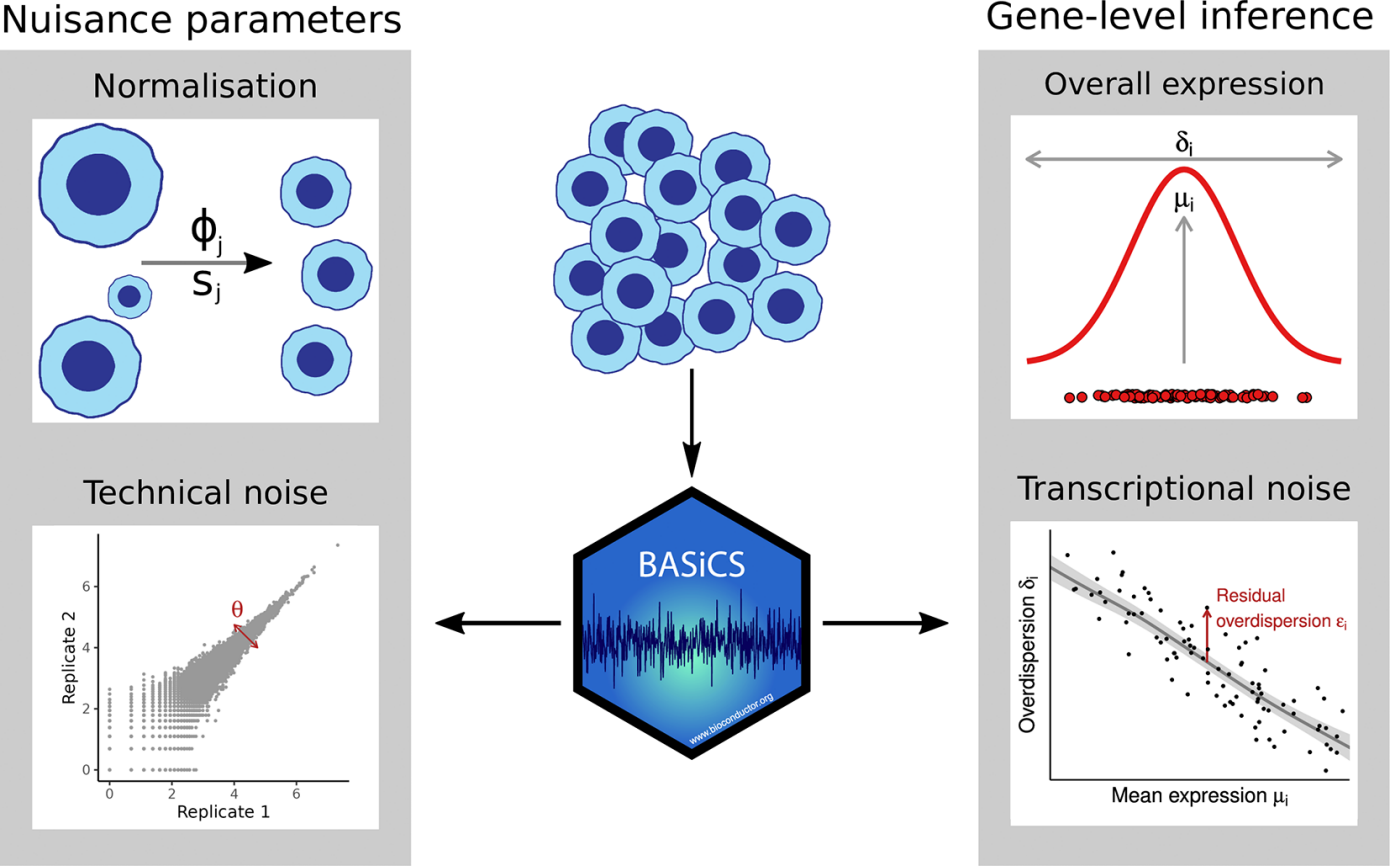
A schematic representation of the features of the BASiCS model. BASiCS accounts for cell-to-cell and batch-to-batch variability using nuisance parameters. Accounting for cell-to-cell and batch-to-batch technical variability allows BASiCS to robustly perform gene-level inference of mean and variability. Furthermore, by accounting for the association between mean and variability in scRNAseq, BASiCS can also infer transcriptional noise using the residual variability parameter *ε*_*i*_.

A high-level overview for the model implemented in *BASiCS* is shown in Figure 1, and a more detailed description is provided in the ExtendedMethods document in the Zenodo repository for this manuscript (see Data availability). Model parameters are designed to capture different aspects of scRNAseq data. First, *nuisance* parameters are used to capture technical artifacts. This includes cell-specific parameters (indexed by a *j* subscript) used to perform global scaling normalisation (similar to the size factors in *scran* ) and global parameters designed to capture technical over-dispersion (this can also be interpreted as measurement error) that is shared by all genes. Note that, whilst *scran* infers cell-specific size factors as a pre-processing step, *BASiCS* uses an integrated approach wherein data normalisation and downstream analyses are performed simultaneously, thereby propagating statistical uncertainty. Secondly, *BASiCS* summarises expression patterns within the population of cells under study (e.g. a experimental condition or cell type) using two sets of gene-specific parameters (indexed by a *i* subscript). Mean parameters *μ _i_* quantify overall expression for each gene *i.* In contrast, *δ _i_* captures the excess of variability that is observed with respect to what would be expected in a homogeneous cell population, beyond technical noise. The latter is used as a proxy to quantify transcriptional variability.

To account for the strong relationship that is typically observed between gene-specific mean expression and over-dispersion estimates, Eling *et al.* ^26^ introduced a *joint prior* specification for these parameters. This joint prior assumes that genes with similar mean expression ( *μ _i_*) have similar over-dispersion parameters *δ _i_.* Effectively, this shrinks over-dispersion estimates towards a global trend that captures the relationship between mean and over-dispersion ( Figure 1). This improves posterior inference for over-dispersion parameters when the data is less informative (e.g. small sample size, lowly expressed genes). ^26^ This information-sharing approach is conceptually similar to that performed by Ref. 28 and others, where sparse data is pooled to obtain more reliable estimates. The global trend is then used to derive gene-specific *residual over-dispersion* parameters *ε _i_* that are not confounded by mean expression. Similar to the DM values implemented in *scran* , these are defined as deviations with respect to the overall trend. These residual over-dispersion values can be used to quantify transcriptional heterogeneity without any confounding with mean expression, though it should be noted that residual over-dispersion may also arise due to structural heterogeneity (e.g. distinct cell sub-types or states). For example, ^26^ computationally explored the impact of unobserved structural heterogeneity by generating artificially contaminated datasets. In contrast, ^7^ showed that changes in transcriptional heterogeneity can relate to aging, where older mice were shown to exhibit a more “bursting” or stochastic transcriptional pattern relative to younger mice.

Additionally, technical variation is quantified using replication. ^36^ In the absence of true technical replicates, we assume that population-level characteristics of the cells are replicated using appropriate experimental design. This requires that cells from the same population have been randomly allocated to different batches. Given appropriate experimental design, *BASiCS* assumes that biological effects are shared across batches, while technical variation leads to spurious differences between cells in different batches. Thus, *BASiCS* models cell-specific size factors *y*_*i*_, and batch-specific technical dispersion *θ*. It is this version of the model that we focus on here, and that we recommend for most users. Previous versions of the model are available within the package, but are primarily useful for reproducibility purposes or for analysing datasets that contain spike-in genes.

While several differential expression tools have been proposed for scRNA-seq data (e.g. Refs. 37, 38), some evidence suggests that these do not generally outperform popular bulk RNA-seq tools. ^39^ Moreover, most of these methods are only designed to uncover changes in overall expression, ignoring the more complex patterns that can arise at the single cell level. ^27^ Instead, *BASiCS* embraces the high granularity of scRNA-seq data, uncovering changes in cell-to-cell expression variability that are not confounded by differences in technical noise or in overall expression.

### Operation

This step-by-step scRNA-seq workflow is primarily based on the Bioconductor package ecosystem ^40^ for the R programming language, ^41^ and as such should run on any major operating system using R ≥ 4.0, although results may differ slightly using different Bioconductor and R versions. A graphical overview is provided in and its main components are described below. The libraries listed below are required for this workflow, all of which can be downloaded from Bioconductor. Alternatively, we provide a Docker image containing all of the software necessary to run *BASiCS* at https://hub.docker.com/r/alanocallaghan/basicsworkflow2020-docker.

**Figure 2.**
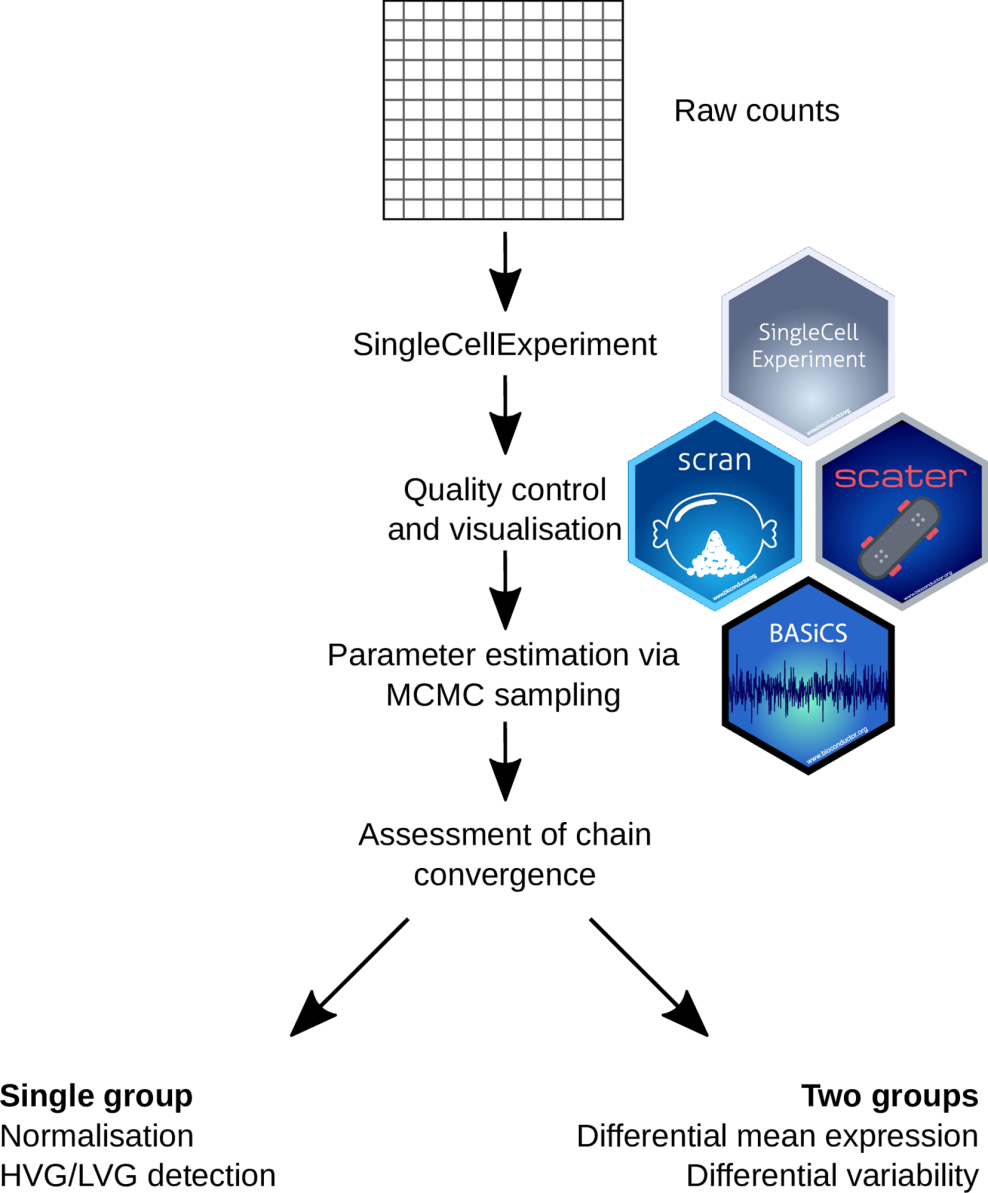
Graphical overview for the single cell RNA sequencing analysis workflow described in this manuscript. Starting from a matrix of expression counts, we use the scater and scran Bioconductor packages to perform QC and initial exploratory analyses. To robustly quantify transcriptional heterogeneity within seemingly homogeneous cell populations, we apply the BASiCS Bioconductor package and illustrate how BASiCS can be used to analyse a single or multiple pre-specified groups of cells.

Here, we load all the libraries that will be used throughout this workflow. First, we load Bioconductor libraries included in Figure 2. We provide further explanation about their function in the following sections.

library("SingleCellExperiment")
library("scater")
library("scran")
library("BASiCS")


Next, we load other general purpose libraries used for data visualisation — e.g., *ggplot2* ), colour scales (e.g., *viridis* , and data manipulation ( *tidyr* ).

# Data visualisation
library("ggplot2")
library("ggpointdensity")
library("patchwork")
library("ComplexHeatmap")
# colour scales
library("viridis")
library("circlize")
library("RColorBrewer")
# data manipulation
library("tidyr")


## Input data

We use the package *SingleCellExperiment* to convert an input matrix of raw read counts (molecule counts for UMI-based protocols) into a SingleCellExperiment object that can also store its associated metadata, such as gene- and cell-specific information. Moreover, when available, the same object can also store read counts for spike-in molecules (see help("altExp")). A major advantage of using a SingleCellExperiment object as the input for scRNA-seq analyses is the interoperability across a large number of Bioconductor packages. ^40^

A critical step in scRNA-seq analyses is QC, removing low quality samples that may distort downstream analyses. The *OSCA* online book provides an extensive overview on important aspects of how to perform QC of scRNA-seq data, including exploratory analyses. ^40^ Here, we use QC diagnostics to identify and remove samples that correspond to broken cells, that are empty, or that contain multiple cells. ^42^ We also remove lowly expressed genes that represent less reliable information.

We recommend the Bioconductor package *scater* ^20^ to calculate QC metrics for each cell (e.g. total read-count) and gene (e.g. percentage of zeroes across all cells), respectively. We also recommend the visualisation tools implemented in the *scater* to explore the input dataset and its associated QC diagnostic metrics. To perform further exploratory data analysis we recommend the Bioconductor package *scran* . ^33^ The latter is used to perform *global scaling* normalisation, calculating cell-specific scaling factors that capture global differences in read-counts across cells (e.g. due to sequencing depth and PCR amplification). ^43^

Note that the use of *scran* as a normalisation tool is not a requirement for *BASiCS* (which uses raw read-counts as input). However, we recommend the use of *scran* normalisation as part of the exploratory data analysis. For example, normalised read-counts can be used to visualise the data in a low-dimensional space (e.g. using principal component analysis). This can help to explore the structure of the data, e.g. to examine potential batch effects.

## Use cases

### Case study: analysis of naive CD4 ^+^ T cells

The development of droplet-based scRNA-seq ^44^^,^^45^ lead to a large increase in the number of cells that can be profiled per experiment at a fixed cost. With this, large-scale scRNA-seq datasets have been generated to study development across multiple time-points and capturing multiple tissues. ^46^^,^^47^

To illustrate the use of *BASiCS* in this context, we use droplet-based scRNAseq generated using the 10X microfluidics platform. An example using a dataset generated using a plate-based protocol including spike-ins is provided in the AnalysisCD4T document of the Zenodo repository for this manuscript (see Data availability). In particular, we focus on the data generated by Ref. 46 which contains expression profiles for 20,819 cells collected from E8.25 mouse embryos. These data comprise a highly heterogeneous population of cells with over 20 major cell types. The data is stored under the accession number E-MTAB-6153 on ArrayExpress. The authors also provide additional information summarising the experimental design (animal of source for each cell) and the cluster labels generated by in their analysis. The code required to download these data is provided in the DataPreparationBASiCSWorkflow document of the Zenodo repository for this manuscript (see Data availability).

As explained above, when analysing a heterogeneous population of cells, a clustering analysis is required to identify the cell types that drive this heterogeneity prior to running *BASiCS* . *BASiCS* can then be used to quantify transcriptional variability within each cell type, as well as to characterise differences between cell types. Here, we use the cluster labels generated in the original publication. For illustration purposes, we focus on analysing presomitic and somitic mesoderm cells. This is an arbitrary choice and the steps described in this workflow could be applied to any other pair of cell types.

### Preparing the input data for BASiCS

While quality control and pre-processing is an important topic in scRNAseq analysis, we feel that it has been covered in detail in other articles. For this analysis, we have performed quality control, pre-processing and exploratory data analysis in the DataPreparationBASiCSWorkflow document of the Zenodo repository for this manuscript (see Data availability). The code presented unprocessed raw data and prepares it for use in this workflow. Here, we load the pre-processed data. This consists of two separate SingleCellExperiment data objects: one for presomitic and one for somitic mesoderm cells.

# Website where the files are located
files_website <- "https://zenodo.org/record/10251224/files/"
# To avoid timeout issues as we are downloading large files
options(timeout = 1000)
# File download
## The code below uses ‘file.exists’ to check if files were previously downloaded
## After download, files are then stored in an ‘rds’ sub-folder
if(!file.exists("rds/sce_sm.Rds")) {
  download.file(
    paste0(files_website, "sce_sm.Rds"),
    destfile = "rds/sce_sm.Rds"
  )
}
if(!file.exists("rds/sce_psm.Rds")) {
  download.file(
    paste0(files_website, "sce_psm.Rds"),
    destfile = "rds/sce_psm.Rds"
  )
}
# Pre-somitic mesoderm cells
sce_psm <- readRDS("rds/sce_psm.Rds")
# Somitic mesoderm cells
sce_sm <- readRDS("rds/sce_sm.Rds")


In brief, we have filtered the original data to select presomitic mesoderm (PSM) and somitic mesoderm (SM) cells, excluding a small cluster of cells which the authors identified a potential doublets (where multiple cells are captured in the same droplet). We also exclude cells with more 25,000 UMI-counts. The processed dataset contains 871 pre-somitic and 724 somitic mesoderm cells.

Subsequently, we applied a gene filtering step to remove lowly expressed genes that were largely undetected through sequencing. In particular, we removed genes that are not detected in at least 20 cells across all cells. This is to ensure a reliable estimate of variability, and is roughly in line with the sample size requirements for the negative binomial distribution. ^48^ We also applied a filter to consider protein-coding genes only. After applying these filters 10862 will be included in our analysis.

Since droplet-based scRNA-seq data are generated without including technical spike-in genes, BASiCS uses measurement error models to quantify technical variation through replication. ^37^ The different experimental batches used by Ref. 46 will be used for this purpose. In the case of the somitic and pre-somoitic mesoderm cells, two mouse embryos have been used to generate the data. Cells isolated from the first embryo were split into two batches and processed independently. Cells from the second embryo were processed together as a single batch. These three different groups of cells are used as batches. Numbers of cells per batch and cell type are displayed below.

# Pre-somitic mesoderm cells
table(sce_psm$sample)
##
## 1.1 1.2   2
## 346 339 186
# Somitic mesoderm cells
table(sce_sm$sample)
##
## 1.1 1.2   2
## 285 284 155


To enable BASiCS to use this information, this should be stored as BatchInfo within the cell-level metadata.

# Pre-somitic mesoderm cells
colData(sce_psm)$BatchInfo <- colData(sce_psm)$sample
# Somitic mesoderm cells
colData(sce_sm)$BatchInfo <- colData(sce_sm)$sample


### Parameter estimation using BASiCS

Parameter estimation is implemented in the BASiCS_MCMC function using an adaptive Metropolis within Gibbs algorithm (see section 3 in Ref. 49). The primary inputs for BASiCS_MCMC correspond to:

• Data: a SingleCellExperiment object created as described in the previous sections.
• N: the total number of MCMC iterations.
• Thin: thining period for output storage (only the Thin-th MCMC draw is stored).
• Burn: the initial number of MCMC iterations to be discarded.
• Regression: if TRUE a joint prior is assigned to *μ*_*i*_ and *δ*_*i*_, ^26^ and residual over-dispersion values εi are inferred. Alternatively, independent log-normal priors are assigned to *μ _i_* and *δ*_*i.*_^25^
• WithSpikes: if TRUE information from spike-in molecules is used to aid data normalisation and to quantify technical noise.
• PriorParam: Defines the prior hyper-parameters to be used by *BASiCS* .

As a default, we recommend to use Regression = TRUE, as the joint prior introduced by Ref. 26 leads to more stable estimation, particularly for small sample sizes and lowly expressed genes. This approach also enables users to obtain a measure of transcriptional variability that is not confounded by mean expression. Advanced users may use the BASiCS_PriorParam function to adjust prior hyperparameters. If PriorMu = "EmpiricalBayes", *μ*_*i*_s are assigned a log-normal prior with gene-specific location hyper-parameters defined via an empirical Bayes framework. Alternatively, if PriorMu = "default", location hyper-parameters are set to be equal 0 for all genes. We recommend that users use the defaults for PriorParam for most applications. We also recommend to use PriorMu = "EmpiricalBayes" as we have observed that an empirical Bayes framework ^50^ improves estimation performance for sparser datasets. Extra parameters can be used to store the output ( StoreChains, StoreDir, RunName) and to monitor the progress of the algorithm ( PrintProgress).

Here, we run the MCMC sampler separately for somitic and pre-somitic mesoderm cells. We use 30,000 iterations ( N), discarding the initial 15,000 iterations as burn-in, ( Burn), and saving parameter values only once in each 15 iterations ( Thin). We recommend this setting for large datasets, as it generally produces reliable and reproducible results. However, specific datasets may require a larger number of iterations to achieve convergence. Practical guidance about MCMC convergence diagnostics is provided in the next section.

## MCMC results may vary slightly due to pseudorandom number generation.
## We fix a random seed for exact reproducibility,
## but this is not strictly required
set.seed(42)
chain_sm <- BASiCS_MCMC(
  Data = sce_sm,
  N = 30000,
  Thin = 15,
  Burn = 15000,
  Regression = TRUE,
  WithSpikes = FALSE,
  PriorParam = BASiCS_PriorParam(sce_sm, PriorMu = "EmpiricalBayes"),
  Threads = 4,
  StoreChains = TRUE,
  StoreDir = "rds/",
  RunName = "SM"
)
set.seed(43)
chain_psm <- BASiCS_MCMC(
  Data = sce_psm,
  N = 30000,
  Thin = 15,
  Burn = 15000,
  Regression = TRUE,
  WithSpikes = FALSE,
  PriorParam = BASiCS_PriorParam(sce_psm, PriorMu = "EmpiricalBayes"),
  Threads = 4,
  StoreChains = TRUE,
  StoreDir = "rds/",
  RunName = "PSM"
)


This first of these chains takes 140 minutes to complete on a 3.4 GHz Intel Core i7 4770k procesor with 32GB RAM, while the second takes 159 minutes. For convenience, these can be obtained online at https://doi.org/10.5281/zenodo.10251224.

# Website were the files are located
chains_website <- "https://zenodo.org/record/10251224/files/"
# To avoid timeout issues as we are downloading large files
options(timeout = 1000)
# File download
## The code below uses ‘file.exists’ to check if files were previously downloaded
## After download, files are then stored in an ‘rds’ sub-folder
if(!file.exists("rds/chain_sm.Rds")) {
  download.file(
    paste0(chains_website, "chain_sm.Rds"),
    destfile = "rds/chain_sm.Rds"
  )
}

if(!file.exists("rds/chain_psm.Rds")) {
  download.file(    paste0(chains_website, "chain_psm.Rds"),
    destfile = "rds/chain_psm.Rds"
  )
}
## Loads files after download
chain_sm <- readRDS("rds/chain_sm.Rds")
chain_psm <- readRDS("rds/chain_psm.Rds")


The output from BASiCS_MCMC is a BASiCS_Chain object that contains the draws associated to all model parameters. Given that ( N - Burn) / Thin = (30,000 - 15,000) /15 = 1,000 the object contains 1,000 draws for each parameter. The matrices of MCMC draws can be accessed using the displayChainBASiCS function — this may be useful for estimating and visualising credible intervals using packages like *bayesplot* or *tidybayes* . For example, the following code displays the first 2 MCMC draws for mean expression parameters associated to the first 3 genes.

displayChainBASiCS(chain_sm, Param = "mu")[1:2, 1:3]
##      ENSMUSG00000025902 ENSMUSG00000033845 ENSMUSG00000025903
## [1,]         0.03320425           3.634436          0.9257323
## [2,]         0.03785246           3.645978          0.9858456


### MCMC convergence diagnostics

Before interpreting the estimates generated by *BASiCS* , it is important to check the performance of the MCMC algorithm used. This algorithm used to characterise the posterior distribution is stochastic by nature, and as such its performance may vary even when used on the same dataset using the same settings. We perform quality control of the MCMC algorithm in order to ensure that the results we observe are the result of properties of the underlying data, rather than being an artefact of the stochastic algorithm used to characterise the data.

As part of performing quality control of MCMC performance, it is critical to assess the convergence of the MCMC algorithm, i.e. whether the MCMC reached its stationary distribution. If convergence has been achieved, the trace for each parameter should not evolve significantly across iterations, as MCMC draws are expected to be stochastic fluctuations around a horizontal trend once the sampler has converged to its stationary distribution. It is not possible to prove convergence, but multiple graphical and quantitative convergence diagnostics have been proposed to assess the lack of convergence (e.g. Refs. 51, 52). Some advocate the use of multiple MCMC chains using different starting values in order to ensure that the algorithm consistently converges to the same distribution and to avoid convergence to local modes. For *BASiCS* , we have observed that using informed starting values (e.g. based on *scran* normalisation factors) and a sufficiently large value for N and Burn generally leads to largely consistent estimates across multiple MCMC runs. Hence, the focus of this section is to evaluate quantitative measures of convergence (e.g. Ref. 53) based on a single MCMC chain.

For illustration purposes, here we focus on the chains generated for the somatic mesoderm cells. However, it is important to analyse convergence for all MCMC chains; we have omitted this analysis for pre-somitic mesoderm cells in the present manuscript for brevity, but they can be viewed in the data preparation document (DataPreparationBASiCSWorkflow) in the Zenodo repository for this manuscript (see Data availability).

**Figure 3.**
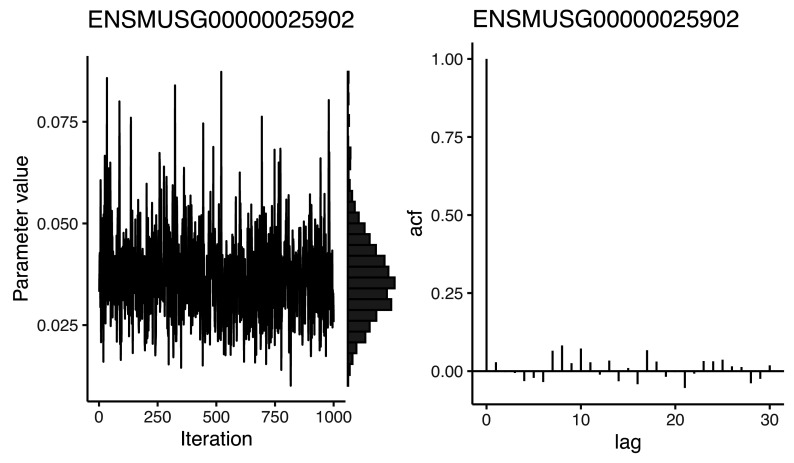
Trace plot, marginal histogram, and autocorrelation function of the posterior distribution of the mean expression parameter for a gene in somitic mesoderm cells following MCMC sampling. Trace plots should explore the posterior without getting stuck in one location or drifting over time towards a region of higher density. High autocorrelation indicates that the number of effective independent samples is low. It is good practice to perform these visualisation for many different parameters; here we only show one.

Traceplots can be used to visually assess the history of MCMC iterations for a specific parameter (e.g., Figure 3 left panel). As mentioned above, significant departures from a horizontal trend suggest a lack of convergence. As illustrated in Figure 3 (right panel), histograms can also be used to display the marginal distribution for each parameter. For *BASiCS* , users should expect these to follow a unimodal distribution. Failure to satisfy these graphical diagnostics suggest that N and Burn must be increased. Alternatively, more stringent quality control could be applied to the input data, as we have observed that genes with very low counts often suffer from slow convergence.

plot(chain_sm, Param = "mu", Gene = 1)


Trace plots should explore the posterior without getting stuck in one location or drifting over time towards a region of higher density. High autocorrelation indicates that the number of effective independent samples is low. It is good practice to perform this visualisation for many different parameters; here we only show one.

**Figure 4.**
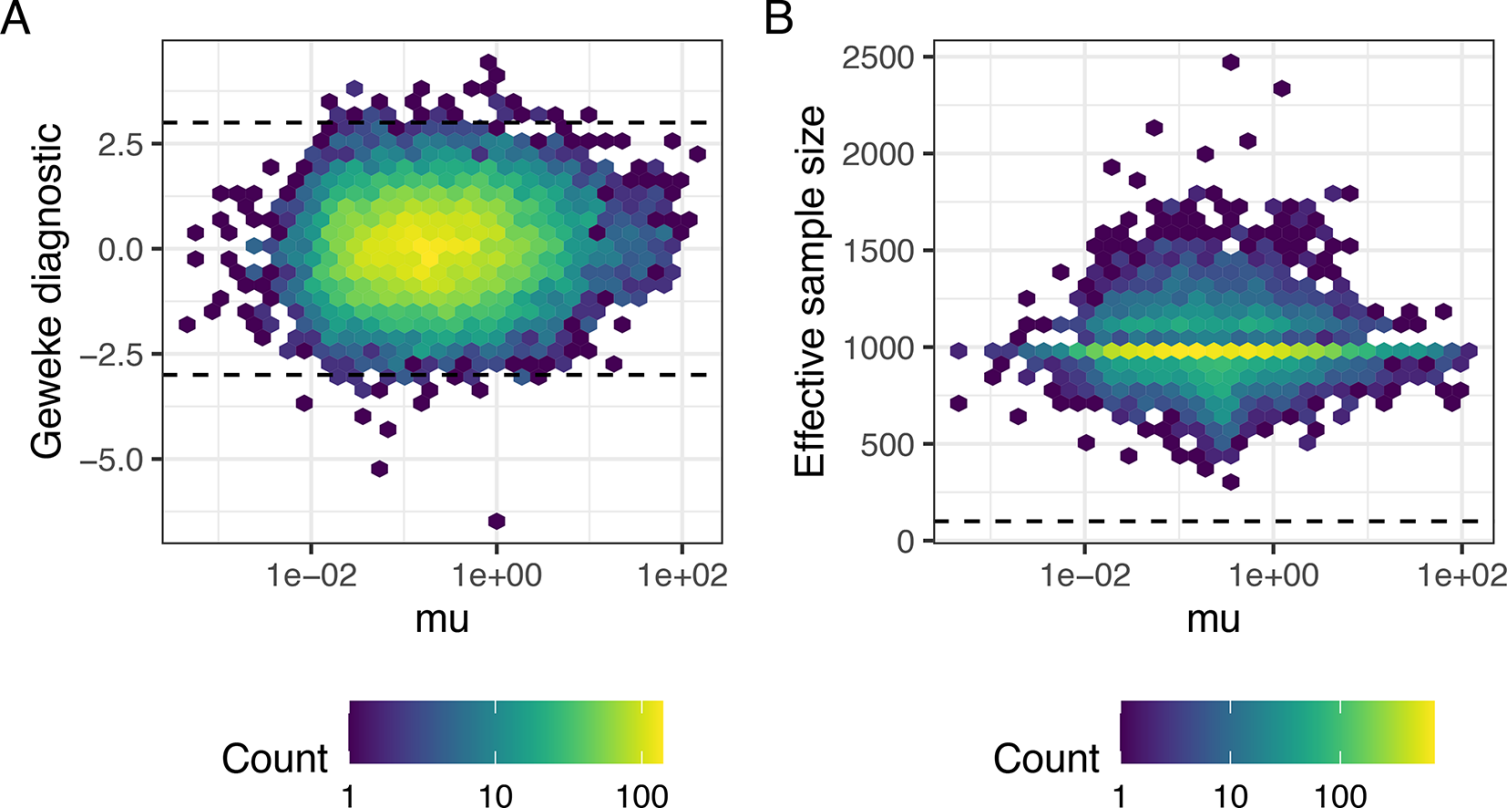
Markov chain Monte Carlo diagnostics for gene-specific mean expression parameters; somitic mesoderm cells. A: Geweke Z-score for mean expression parameters is plotted against mean expression estimates. Dashed lines represent absolute Z-scores of 3, outside of which we advise caution when interpreting results. B: Effective sample size (ESS) is plotted against mean expression estimates. A dashed line shows a threshold of 100, below which we advise caution when interpreting results.

As *BASiCS* infers thousands of parameters, it is impractical to assess these diagnostics separately for each parameter. Thus, it is helpful to use numerical diagnostics that can be applied to multiple parameters simultaneously. The function BASiCS_DiagPlot can be used to visualise such diagnostic metrics. Here, we illustrate usage for two such metrics focusing on the MCMC chain that was obtained for the somitic mesoderm cells (similar results were obtained for pre-somitic mesoderm cells in the DataPreparationBASiCSWorkflow document of the Zenodo repository (see Data availability)). First, we focus on the diagnostic criterion proposed by Geweke. ^53^ The latter compares the average of draws obtained during the initial (10% after burn in, by default) and the final part of the chain (50% by default) by calculating Z-scores of the relative difference between the two sets of draws. Large absolute Z-scores suggest that the algorithm has not converged (as a rule of thumb, a threshold at |Z| < 3 is often applied). For the somitic and pre-somitic mesoderm datasets, most Z-scores associated to mean expression parameters *μ*_*i*_ were small in absolute value (see Figure 4A), suggesting that the algorithm has largely converged.

As well as assessing MCMC convergence, it is important to ensure that the MCMC algorithm has efficiently explored the parameter space. For example, the autocorrelation function (e.g. Figure 3, right panel) quantifies the correlation between the chain and its lagged versions. Strong autocorrelation indicates that neighbouring MCMC samples are highly dependent and suggest poor sampling efficiency. The latter may indicate that the MCMC draws do not contain sufficient information to produce accurate posterior estimates. In other words, highly correlated MCMC samplers require more samples to produce the same level of Monte Carlo error for an estimate (defined as the variance of a Monte Carlo estimate across repetitions). ^54^

The effective sample size (ESS) is a related measure which represents a proxy for the number of independent draws generated by the MCMC sampler. ^55^ The latter is defined as:

$$\;\mathrm{ESS}=\frac{N_{\mathrm{tot}}}{1+2\sum_{k=1}^{\infty }\rho \left(k\right)},$$

where *N*_*tot*_ represents the total number of MCMC draws (after burn-in and thining) and *ρ*( *k*) is the autocorrelation at lag *k*. ESS estimates associated to mean expression parameters for the somitic mesoderm cells are displayed in Figure 4B. Whilst ESS is around 1,000 ( *N*_*tot*_ in this case) for most genes, we observe low ESS values for a small proportion of genes. As described later in this manuscript, BASiCS_TestDE automatically excludes genes with low ESS during differential expression testing (by default a threshold at ESS <100 is applied). However, if a large number of genes have large Geweke diagnostic values or low effective sample sizes in a certain dataset, then caution should be applied when interpreting the results of the model. These issues can often be addressed by more stringent filtering of genes and cells before performing inference or by increasing the number of iterations.

diag_p1 <- BASiCS_DiagPlot(chain_sm, Param = "mu", Measure = "geweke") +
  theme(legend.position = "bottom")
diag_p2 <- BASiCS_DiagPlot(chain_sm, Param = "mu", Measure = "ess") +
  theme(legend.position = "bottom")
diag_p1 + diag_p2 + plot_annotation(tag_levels = "A")


### Quantifying transcriptional variability using BASiCS

**Figure 5.**
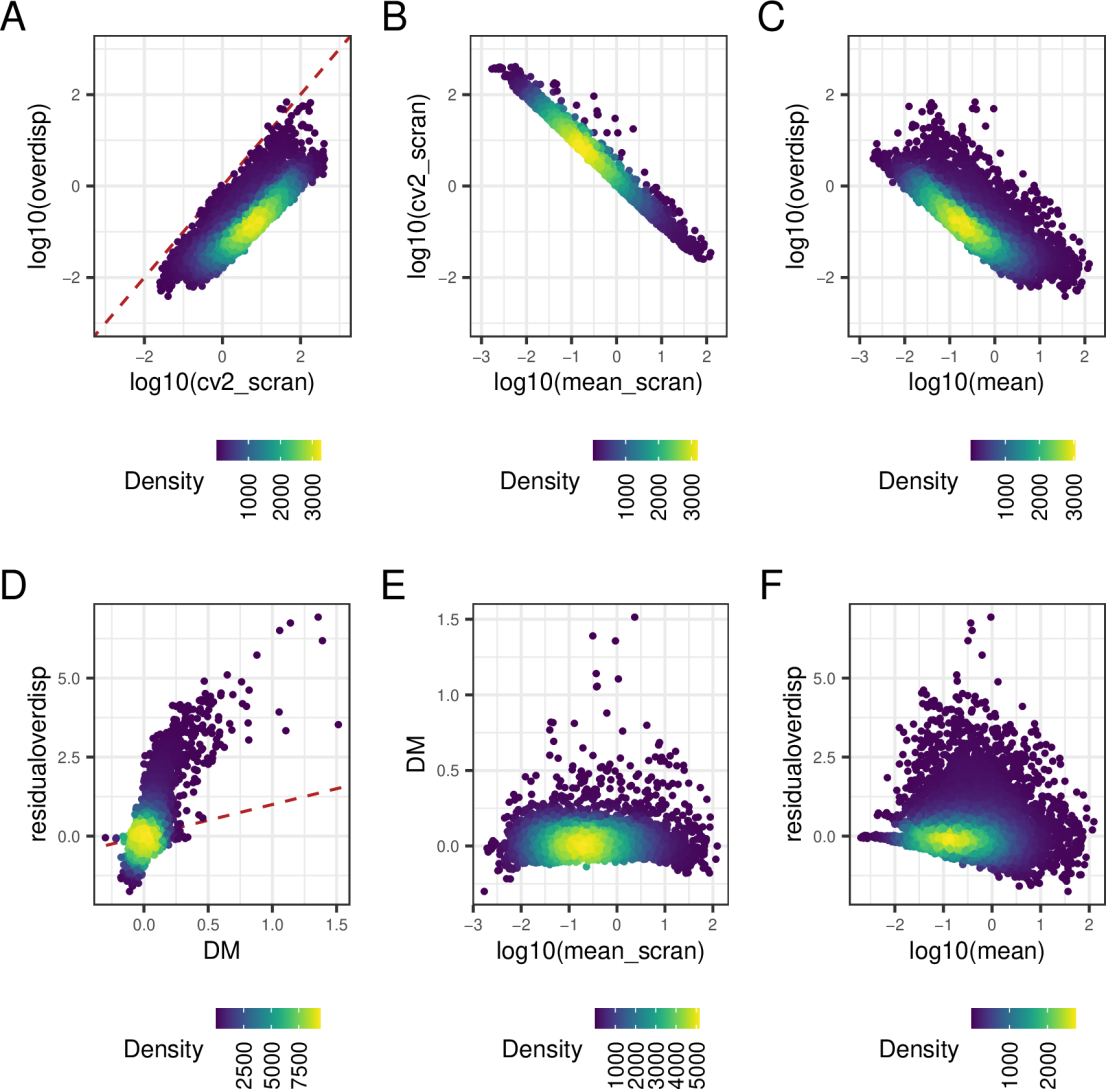
Comparison of gene-specific transcriptional variability estimates and mean expression estimates obtained for each gene using BASiCS and scran. For this analysis, we exclude genes that are not expressed in at least 2 cells. BASiCS estimates for each gene are defined by the posterior median of the associated parameter. scran estimates for each gene are derived after applying the pooling normalisation strategy proposed by Lun et al. Points are coloured according to the local density of genes along the x- and y- axis. A: scran squared CV estimates versus BASiCS estimates for over-dispersion parameters. B: scran estimates for mean expression and the squared CV. C: BASiCS estimates for mean expression and over-dispersion parameters. D: BASiCS estimates for residual over-dispersion parameters versus DM values estimated by scran. E: scran estimates for mean expression and DM values. F: BASiCS estimates for mean expression and residual over-dispersion parameters. Dashed red lines in panels A and D represent the line given by x=y.

Studying gene-level transcriptional variability can provide insights about the regulation of gene expression, and how it relates to the properties of genomic features (e.g. CpG island composition), ^16^ transcriptional dynamics ^56^ and aging, ^7^ among others. The squared coefficient of variation (CV ^2^) is widely used as a proxy for transcriptional variability. For example, we can obtain CV ^2^ estimates for each gene using *scran* normalised counts as input. In contrast, *BASiCS* infers transcriptional variability using gene-specific over-dispersion parameters *δ*_*i*_ (see *Methods*). Figure 5 compares these approaches, focusing on somitic mesoderm cells (repeating this analysis for pre-somitic mesoderm cells led to similar results). For each of the panels in Figure 5, we use the R package *ggpointdensity* to visualise the local density of genes along the axes. Note that the BASiCS_ShowFit function can be used to generate Figure 5C, but we generated the plot manually to demonstrate how users can extract this information from a BASiCS_Chain object, and for visual consistency with the other panels.

As seen in Figure 5A, CV ^2^ and posterior estimates for are highly correlated. Moreover, both variability metrics are confounded by differences in mean expression, i.e. highly expressed genes tend to exhibit lower variability ( Figures 5B-C). We note, however, that *scran* infers a more narrow distribution of transcriptional variability estimates for any given mean expression level. To remove the confounding observed in Figures 5B-C, *scran* and *BASiCS* derive *residual variability* estimates as deviations with respect to an global mean-variability trend (see *Methods*). These are derived using the DM approach ^57^ and the residual over-dispersion parameters defined by Ref. 26, respectively. For the somitic mesoderm cell data, both approaches led to correlated estimates ( Figure 5D, Pearson’s correlation equal to 0.75) but the residual variability estimates generated for these data are less consistent than what we have observed in the context of less sparse data (see the AnalysisCD4T document in the Zenodo repository for this manuscript (see Data availability). For the majority of genes (7,697 out of 10,850 captured in at least 3 cells) both approaches led some broadly similar results, inferring the same sign for their residual variability estimates.

## Get BASiCS point estimates for mean and variability - SM cells
summary_sm <- Summary(chain_sm)
parameter_df <- data.frame(
  mean = displaySummaryBASiCS(summary_sm, Param = "mu")[, 1],
  overdisp = displaySummaryBASiCS(summary_sm, Param = "delta")[, 1],
  residualoverdisp = displaySummaryBASiCS(summary_sm, Param = "epsilon")[, 1]
)

## Calculate scran size factors - SM cells
sce_sm <- computeSumFactors(sce_sm)
## Get scran estimates for mean and variability - SM cells
sce_sm <- logNormCounts(sce_sm, log = FALSE)
parameter_df$mean_scran <- rowMeans(assay(sce_sm, "normcounts"))
parameter_df$cv2_scran <- rowVars(assay(sce_sm, "normcounts")) /
  parameter_df$mean_scran^2
parameter_df$DM <- DM(
  mean = parameter_df$mean_scran,
  cv2 = parameter_df$cv2_scran
)

## Remove genes without counts in > 2 cells - BASiCS estimates not provided
sel_genes <- !is.na(parameter_df$residualoverdisp)

## The plots below are generated using ggplot2
## We use ‘plot_params’ to specify the format of
## such plots whilst avoiding code duplication
## The plots can be further customized using ggplot2 principles
## For example, we modify x- and y-axis limits in some of the plots below
plot_params <- list(
  geom_pointdensity(size = 0.6, na.rm = TRUE),
  scale_colour_viridis(name = "Density"),
  theme(
    text = element_text(size = rel(3)),
    legend.position = "bottom",
    legend.text = element_text(angle = 90, size = 8, hjust = 0.5, vjust = 0.5),
    legend.key.size = unit(0.018, "npc")
  )
)

g1 <- ggplot(parameter_df[sel_genes,], aes(log10(cv2_scran), log10(overdisp))) +
  xlim(-3, 3) + ylim(-3, 3) +
  geom_abline(
    slope = 1,
    intercept = 0,
    colour = "firebrick",
    linetype = "dashed"
  )

g2 <- ggplot(parameter_df[sel_genes,]) +
  aes(log10(mean_scran), log10(cv2_scran)) +
  xlim(-3, 2.2) + ylim(-3, 3)

g3 <- ggplot(parameter_df[sel_genes,], aes(log10(mean), log10(overdisp))) +
  xlim(-3, 2.2) + ylim(-3, 3)

g4 <- ggplot(parameter_df[sel_genes,], aes(DM, residualoverdisp)) +
  geom_abline(
    slope = 1,
    intercept = 0,
    colour = "firebrick",
    linetype = "dashed"
  )

g5 <- ggplot(parameter_df[sel_genes,], aes(log10(mean_scran), DM))

g6 <- ggplot(parameter_df[sel_genes,], aes(log10(mean), residualoverdisp))

((g1 + g2 + g3) * plot_params) / ((g4 + g5 + g6) * plot_params) +
   plot_annotation(tag_levels = "A") & theme(plot.tag = element_text(size = 15))


### HVG/LVG detection using BASiCS

In *BASiCS* , the functions BASiCS_DetectHVG and BASiCS_DetectLVG can be used to identify genes with substantially high (HVG) or low (LVG) transcriptional variability within a population of cells. If the input BASiCS_Chain object was generated by BASiCS_MCMC with Regression = TRUE (recommended setting), this analysis is based on the posterior distribution obtained for gene-specific residual over-dispersion parameters *ε*_*i*_ (alternatively, the approach introduced by Ref. 24 can be used). HVGs are marked as those for which *ε*_*i*_ exceeds a pre-defined threshold with high probability, where the probability cut-off is chosen to match a given expected false discovery rate (EFDR; by default the target EFDR is set to 10%). ^58^ The expected false discovery rate we use is conceptually similar to false discovery rate control procedures in frequentist statistics, such as the approach of Ref. 59, aiming to control the proportion of false discoveries produced by the procedure. A similar approach is implemented for LVG detection, but based on whether *ε*_*i*_ is below a pre-specified threshold EpsilonThreshold. For example, if the threshold for *ε*_*i*_ is equal to log2 (the default), HVGs would be those genes for which the over-dispersion is estimated to be at least two times higher than would be expected given the inferred mean expression level, while LVGs would be those genes for which the residual over-dispersion is at least two times lower than would be expected on the same basis. In some circumstances, it may be of interest to rank genes and to select those with the highest or the lowest residual over-dispersion, which can be performed using the PercentileThreshold parameter; see help("BASiCS_DetectVG") for more details.

## Highly variable genes
hvg <- BASiCS_DetectHVG(chain_sm, EpsilonThreshold = log(2))
## For HVG detection task:
## the posterior probability threshold chosen via EFDR calibration is too low.
## Probability threshold automatically set equal to 'ProbThreshold'.
## Lowly variable genes
lvg <- BASiCS_DetectLVG(chain_sm, EpsilonThreshold = -log(2))


For subsequent visualisation and data exploration, here we merge the output tables generated above and store these as part of the gene-level metadata in the sce_sm object.

## Merge HVG and LVG tables in a single data frame
vg_table <- merge(
  as.data.frame(lvg, Filter = FALSE),
  as.data.frame(hvg, Filter = FALSE),
  by = c("GeneName", "GeneIndex", "Mu", "Delta", "Epsilon"),
  suffixes = c("LVG", "HVG")
)
## Mark genes as highly variable, lowly variable, or not either.
vg_table$VG <- "Not HVG or LVG"
vg_table$VG[vg_table$HVG] <- "HVG"
vg_table$VG[vg_table$LVG] <- "LVG"
## Store as gene-level metadata
rowData(sce_sm) <- merge(
  rowData(sce_sm), vg_table,
  by.x = "ensembl_gene_id", by.y = "GeneName",
  sort = FALSE
)


**Figure 6.**
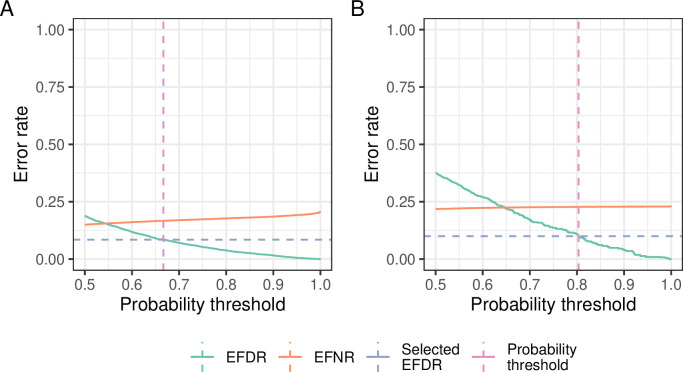
Lines representing the EFDR and EFNR for a range of posterior probability thresholds for the HVG (A) and LVG (B) detection tasks. The target EFDR is indicated by dashed horizontal lines, while the chosen posterior probability threshold is indicated by a vertical dashed line.

Performing HVG and LVG detection involves identifying a posterior probability threshold that matches a target EFDR. We perform this choice using a grid search in which EFDR is calculated for a range of different probability thresholds between 0.5 and 1. As seen in Figure 6, for the somitic mesoderm cells, this leads to a threshold of 0.6666667 and 0.8035 for HVG and LVG detection, respectively (the value of these thresholds can be extracted from the ProbThreshold slot in the hvg and lvg objects, e.g. using hvg@ProbThreshold). For some datasets, the grid search may fail to identify a probability threshold that matches the target EFDR. In such cases, the default minimum probability threshold of 2/3 is used. This default threshold value can be changed using the 'ProbThreshold' argument when calling the 'BASiCS_DetectLVG' and 'BASiCS_DetectHVG' functions.

p1 <- BASiCS_PlotVG(hvg, Plot = "Grid")
p2 <- BASiCS_PlotVG(lvg, Plot = "Grid")

p1 + p2 +
  plot_annotation(tag_levels = "A") +
  plot_layout(guides = "collect") &
  theme(legend.position = "bottom")

plotRowData(sce_sm, x = "Mu", y = "Epsilon", colour_by = "VG") +
  scale_x_log10() +
  geom_hline(yintercept = c(-log(2), 0, log(2)), lty = 2) +
  labs(
    x = "BASiCS means (log10)",
    y = "BASiCS residual\nover-dispersion"
  )


**Figure 7.**
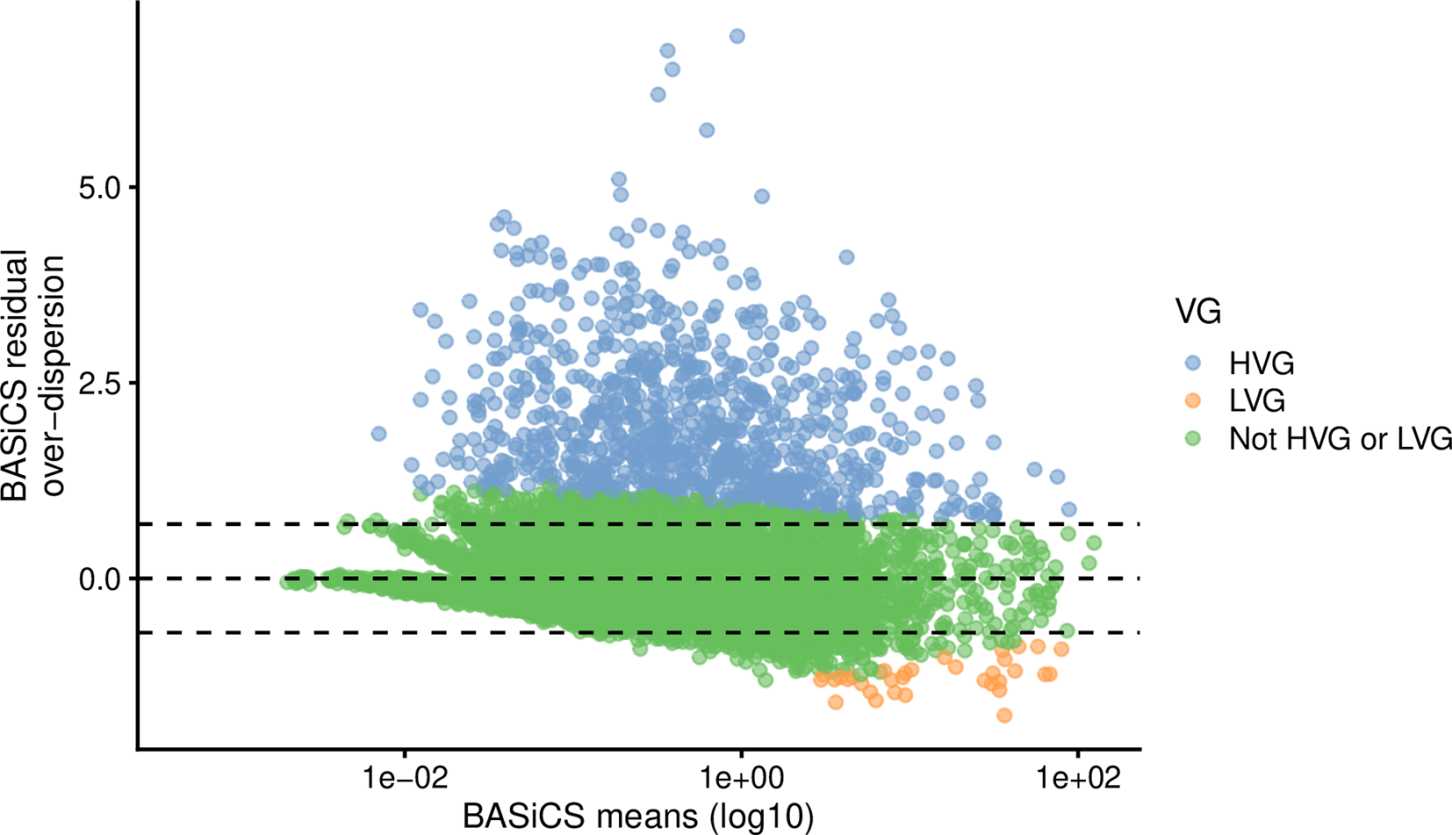
HVG and LVG detection using BASiCS. For each gene, BASiCS posterior estimates (posterior medians) associated to mean expression and residual over-dispersion parameters are plotted. Genes are coloured according to HVG/LVG status. Genes that are not expressed in at least 2 cells are excluded.

**Figure 8.**
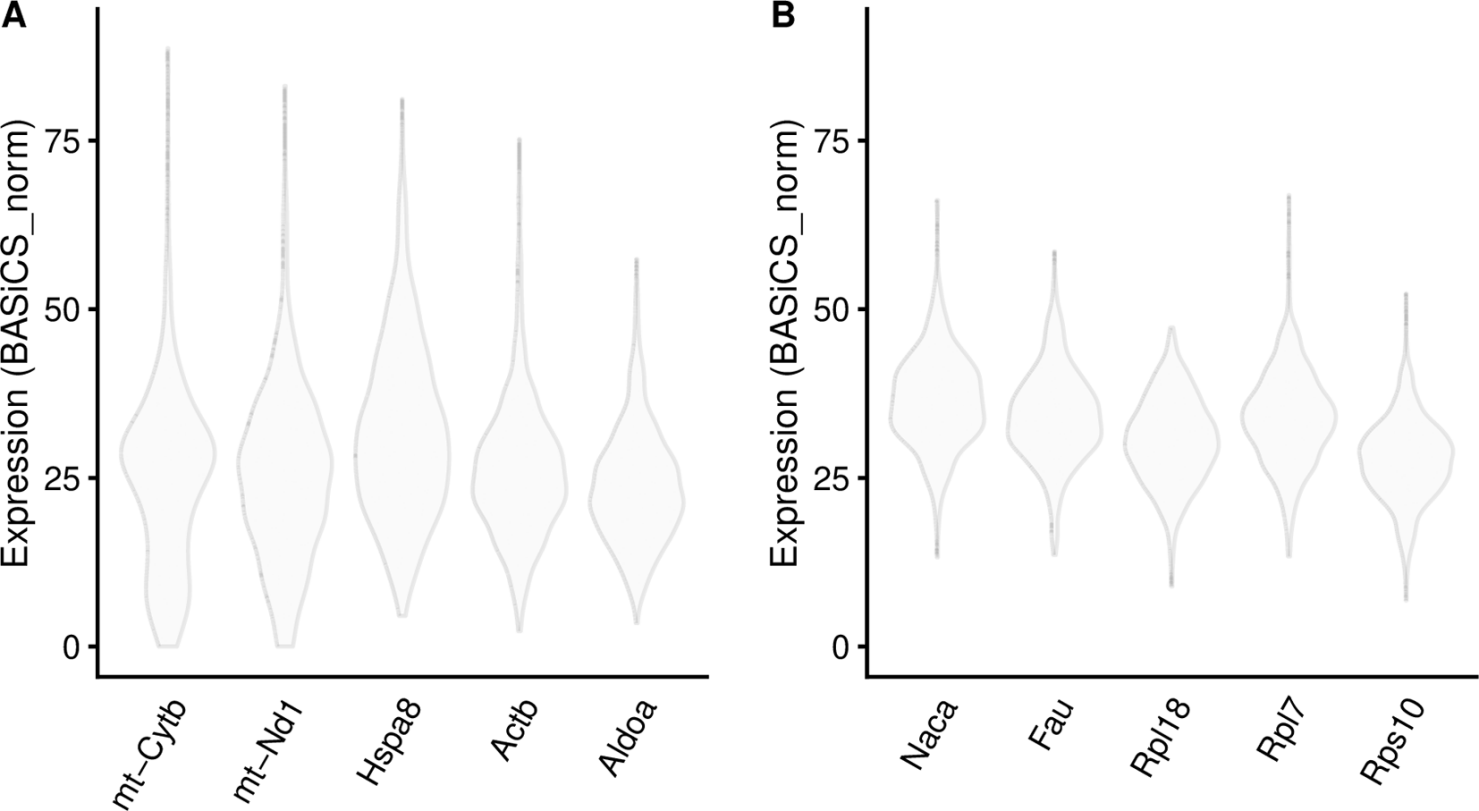
BASiCS denoised counts for example HVG (A) and LVG (B) with similar overall levels of expression.

For the somitic mesoderm cells data, we obtained *923* HVG and *33* LVG. As shown in Figure 7, these genes are distributed across a wide range of mean expression values. As an illustration, Figure 8 shows the distribution of normalised expression values for selected HVG and LVG, focusing on examples with similar mean expression levels. As expected, HVG tend to exhibit a wider and potentially bimodal distribution ( Figure 8A). Instead, LVG tend to have more narrow and unimodal distributions ( Figure 8B).

## Obtain normalised expression values and store them as an assay in sce_sm
assay(sce_sm, "BASiCS_norm") <- BASiCS_DenoisedCounts(
  sce_sm, chain_sm, WithSpikes = FALSE
)

## Select HVG/LVG genes with similar mean expression values
low_exp <- 20
up_exp <- 40
is_mid_exp <- rowData(sce_sm)$Mu > low_exp & rowData(sce_sm)$Mu < up_exp
is_hvg <- is_mid_exp & rowData(sce_sm)$HVG
is_lvg <- is_mid_exp & rowData(sce_sm)$LVG

## Amongst those, order by epsilon and select top 5 HVG and LVG
top_hvg <- order(rowData(sce_sm)$Epsilon[is_hvg], decreasing = TRUE)[1:5]
top_lvg <- order(rowData(sce_sm)$Epsilon[is_lvg], decreasing = FALSE)[1:5]

## Generate violin plots for the selected HVG and LVG
## using scater::plotExpression
plot_hvg <- plotExpression(sce_sm,
    rowData(sce_sm)$external_gene_name[is_hvg][top_hvg],
    exprs_values = "BASiCS_norm",
    swap_rownames = "external_gene_name",
    feature_colours = FALSE,
    point_fun = function(...) list() # disables drawing points
  ) +
  ylim(0, 90)

plot_lvg <- plotExpression(
    sce_sm,
    rowData(sce_sm)$external_gene_name[is_lvg][top_lvg],
    exprs_values = "BASiCS_norm",
    swap_rownames = "external_gene_name",
    feature_colours = FALSE,
    point_fun = function(...) list() # disables drawing points
  ) +
  ylim(0, 90)

plot_hvg + plot_lvg + plot_annotation(tag_levels = "A")


### Differential mean and variability testing using BASiCS

This section highlights the use of *BASiCS* to perform differential expression tests for mean and variability between different pre-specified populations of cells and experimental conditions. Here, we compare the somitic mesoderm cells, analysed in the previous section, to pre-somitic mesoderm cells analysed in the same study. Differential expression testing is performed via the BASiCS_TestDE function. The main input parameters are

• Chain1 and Chain2: two BASiCS_Chain objects created via the BASiCS_MCMC function. Each object corresponds to a different pre-specified group of cells.
• EpsilonM and EpsilonR: introduce a minimum effect size (in a log2 fold change scale) for the detection of changes in mean or residual over-dispersion, respectively. This enables us to discard small expression changes that are less biologically meaningful. By default, we set these thresholds to be equivalent to a 50% change between the groups. However, different thresholds may be required depending on the context. For example, if most genes show strong differences in mean expression, it can be beneficial to increase the value of EpsilonM to focus on strong changes in mean expression.
• EFDR_M and EFDR_R: define the target EFDR to calibrate the decision rule associated to changes in mean or residual over-dispersion, respectively. Default: 10%.
• MinESS: genes with ESS values below this threshold will be excluded from the differential expression tests. This is used to increase the robustness of the results, excludes genes for which the sampler explored the parameter space less efficiently (see *MCMC diagnostics* Section). Default: MinESS = 100.


## Perform differential testing
test_de <- BASiCS_TestDE(
  Chain1 = chain_sm,
  Chain2 = chain_psm,
  GroupLabel1 = "SM",
  GroupLabel2 = "PSM",
  EFDR_M = 0.1,
  EFDR_R = 0.1,
  MinESS = 100,
  Plot = FALSE,
  PlotOffset = FALSE
)
table_de_mean <- as.data.frame(
  test_de,
  Parameter = "Mean",
  Filter = FALSE
)
table_de_resdisp <- as.data.frame(
  test_de,
  Parameter = "ResDisp",
  Filter = FALSE
)
## combine results of differential mean and residual over-dispersion tests
table_de <- merge(table_de_mean, table_de_resdisp)
## Merge with gene-level metadata to obtain gene names used in figures
table_de <- merge(
  table_de,
  rowData(sce_sm)[, c("ensembl_gene_id", "external_gene_name")],
  by.x = "GeneName", by.y = "ensembl_gene_id"
)
# convert to standard data.frame, not S4 DFrame, for ggplot2
table_de <- as.data.frame(table_de)


After running the test, it is important to visualise the results to facilitate interpretation and to identify systematic patterns among differentially expressed genes. It may also be useful to perform functional enrichment analysis to identify biologically meaningful patterns among these genes. For example, this could be performed using the Bioconductor package *goseq* . ^60^ We do not perform this here, but a relevant workflow is described by Maksimovic *et al*. ^61^

**Figure 9.**
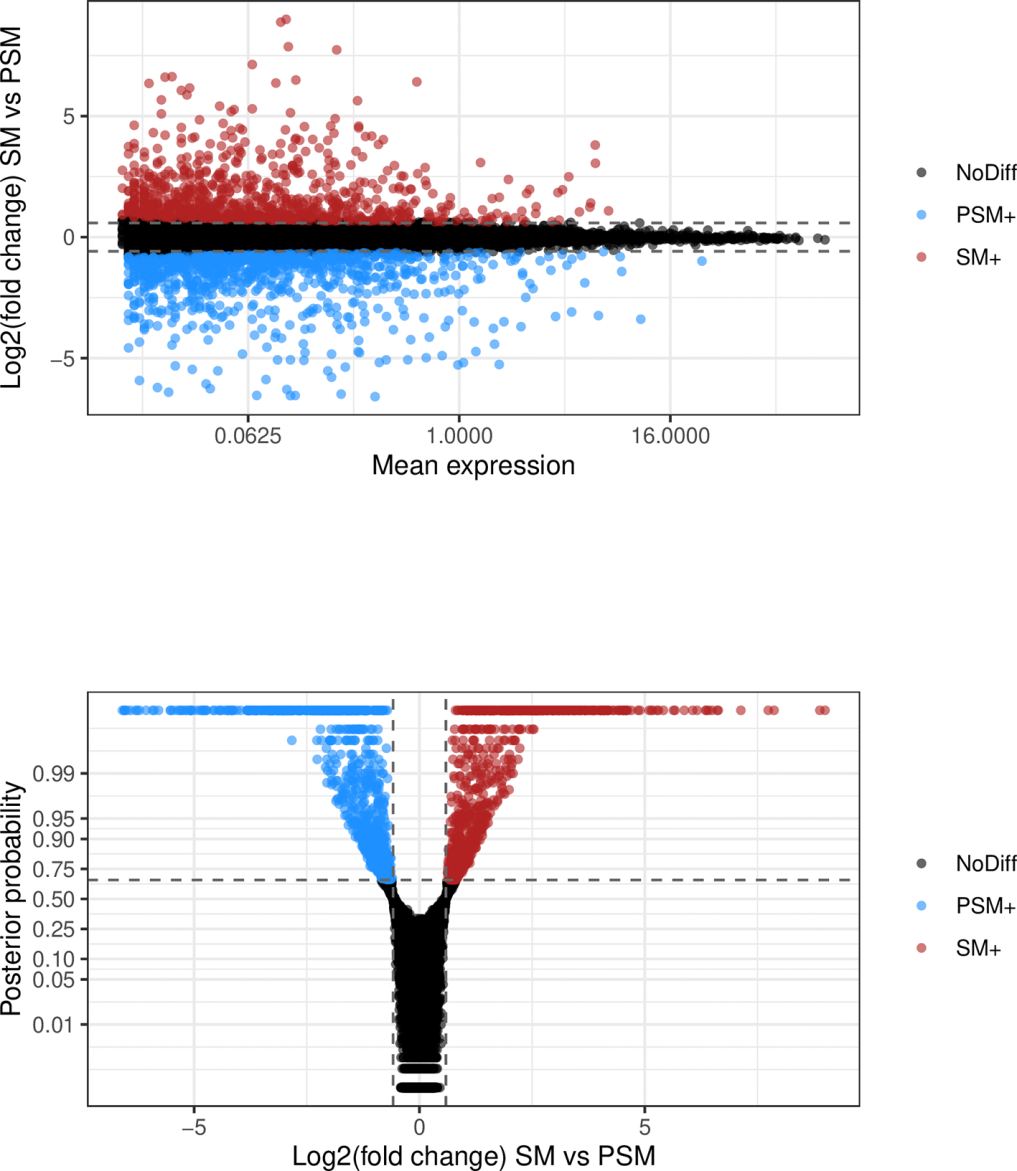
Upper panel presents the mean-difference plot associated to the differential mean expression test between somitic and pre-somitic cells. Log-fold changes of average expression in somitic cells relative to pre-somitic cells are plotted against average expression estimates combined across both groups of cells. Bottom panel presents the volcano plot associated to the same test. Log-fold changes of average expression in somitic cells relative to pre-somitic mesoderm cells are plotted against their associated tail posterior probabilities. Colour indicates the differential expression status for each gene, including a label to identify genes that were excluded from differential expression test due to low ESS.

We first focus on the differential mean expression test. MA-plots (log fold change M versus mean average A) and volcano plots (posterior probability versus log fold change) are popular graphical summaries in this context, and are presented in Figure 9. These can be useful in ensuring that suitable magnitude and confidence thresholds have been chosen. In this instance, it is clear that a large number of genes are differentially expressed between the two conditions, and the selected probability threshold is suitable.

p1 <- BASiCS_PlotDE(test_de, Parameters = "Mean", Plots = "MA")
p2 <- BASiCS_PlotDE(test_de, Parameters = "Mean", Plots = "Volcano",
  TransLogit = TRUE) ## logit-transforms the Y-axis, which can be clearer.
p1/p2


When interpreting the results of differential expression tests, it is useful to visualise expression patterns for differentially expressed genes in order to appraise the significance of the results and guide interpretation. For this purpose, we obtain normalised expression values for each group of cells after correcting for global changes in overall expression between the groups, i.e. a global offset that leads to uniformly higher expression across genes within one group of cells (such step is also internally done by BASiCS_TestDE). Among other causes, the latter can be due to overall differences in mRNA content between the groups.

## Calculate a global offset between groups, using the SM population as reference
offset <- BASiCS_CorrectOffset(chain_psm, chain_sm)
offset$Offset
## [1] 0.9389425
## Get offset corrected chain
chain_psm <- offset$Chain
## Obtain normalised counts within for the PSM population
## Normalised counts were for the SM population were already obtained
## in the HVG/LVG analysis section
## Obtain normalised expression values and store them as an assay in sce_sm
assay(sce_psm, "BASiCS_norm") <- BASiCS_DenoisedCounts(
  sce_psm, chain_psm,
  WithSpikes = FALSE
)
denoised_counts <- SingleCellExperiment(
  assays = list(
    BASiCS_norm = cbind(
      assay(sce_psm, "BASiCS_norm"),
      assay(sce_sm, "BASiCS_norm")
    )
  ),
  colData = data.frame(
    Celltype = c(
      rep("presomitic", ncol(sce_psm)),
      rep("somitic", ncol(sce_sm))
    )
  )
)


**Figure 10.**
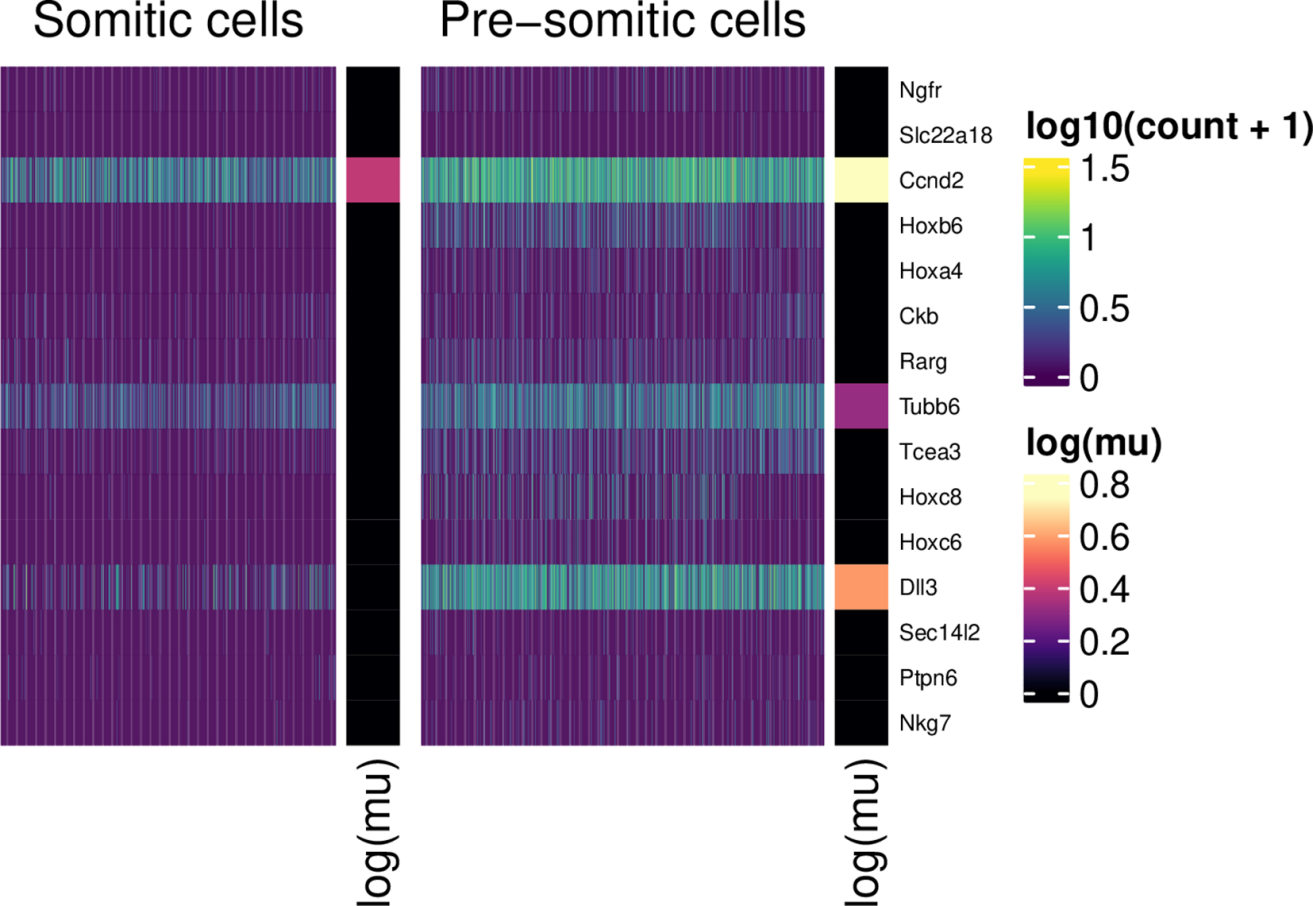
Normalised expression values (scale) for somitic and pre-somitic cells, showing genes up-regulated in PSM cells. For each group, 10 example genes are shown. These were selected according to the ranking of their associated tail posterior probabilities associated to the differential mean expression test. Colour indicates expression level; colour bars on the right of heatmap segments indicate the inferred mean expression level (in log scale) for each gene in each population.

**Figure 11.**
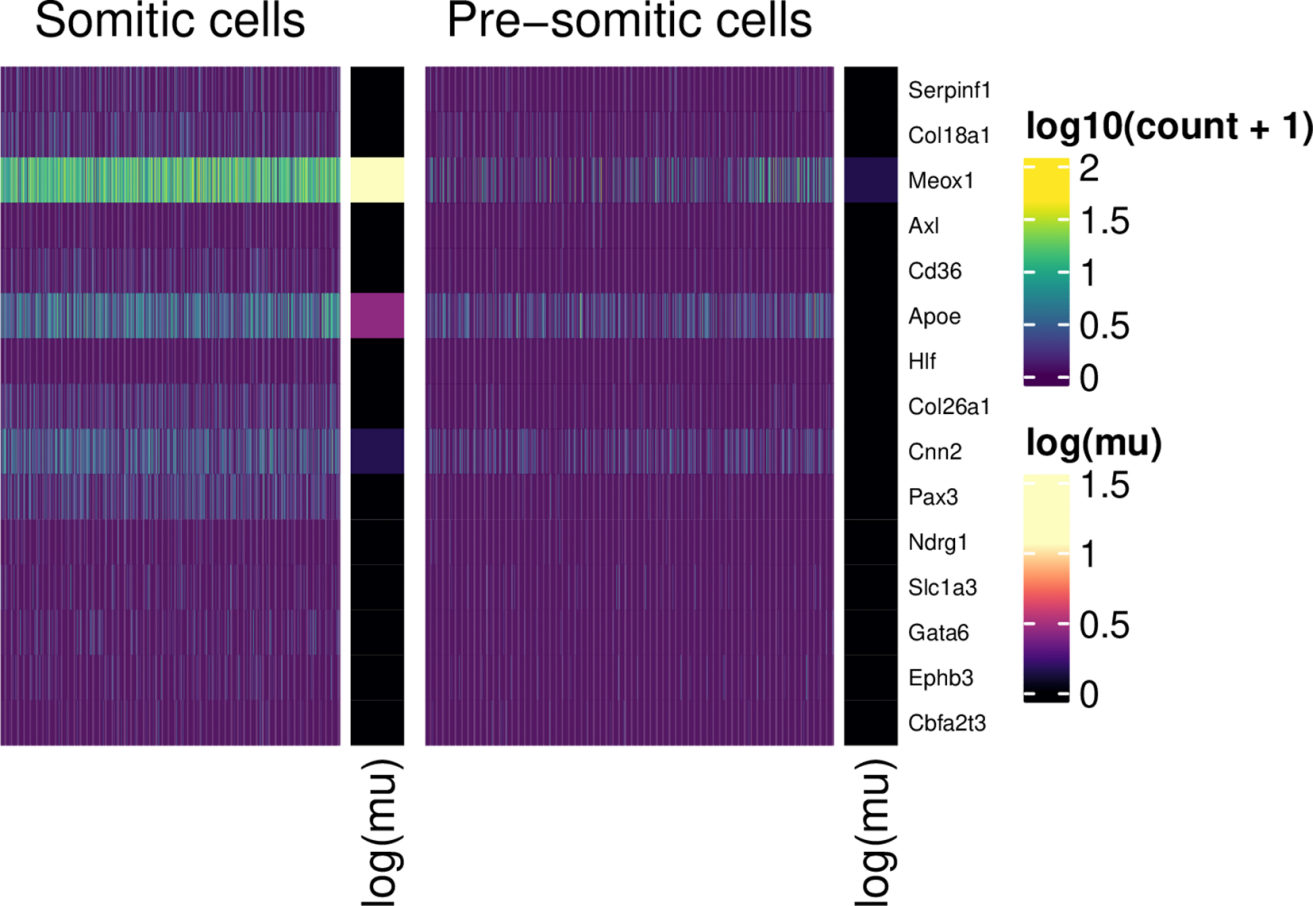
Normalised expression values (scale) for somitic and pre-somitic cells, showing genes up-regulated in SM cells. For each group, 10 example genes are shown. These were selected according to the ranking of their associated tail posterior probabilities associated to the differential mean expression test. Colour indicates expression level; colour bars on the right of heatmap segments indicate the inferred mean expression level (in log scale) for each gene in each population.

**Figure 12.**
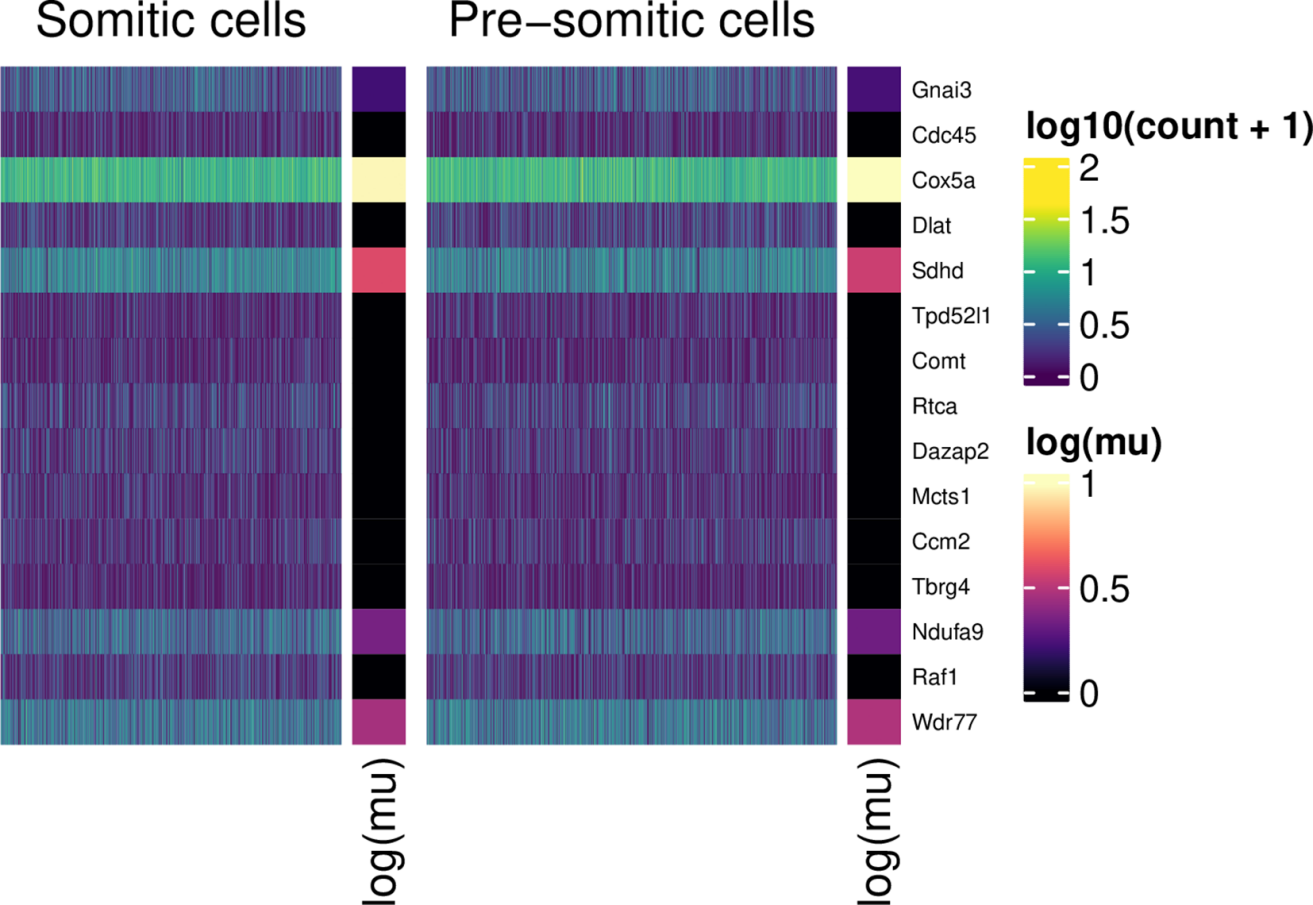
Normalised expression values (scale) for somitic and pre-somitic cells, showing non-differentially expressed genes. For each group, 15 example genes are shown. These were selected according to the ranking of their associated tail posterior probabilities associated to the differential mean expression test. Colour indicates expression level; colour bars on the right of heatmap segments indicate the inferred mean expression level (in log scale) for each gene in each population.

To visualise expression patterns for multiple genes at once, we use the Bioconductor package *ComplexHeatmap* ^62^ package, grouping genes according to the result of the differential mean expression test (i.e. up-regulated in somitic/pre-somitic cells or non differentially expressed; see Figures 10, 11 and 12). For example, among the non DE group, we observe *Cox5a*, a gene essential to mitochondrial function. Such visualisations may aid in the interpretation of such stable or “housekeeping” genes, as well as genes which are up- or down-regulated in each population.

To do this in each dataset, we create a few small utility functions, starting with a function to select the genes with highest probability of being differentially expressed, or the genes with smallest probability of being differentially expressed:

select_top_n <- function(table, condition, n=15, decreasing=TRUE) {
  ind_condition <- table$ResultDiffMean == condition
  table <- table[ind_condition,]
  ind_diff <- order(table$ProbDiffMean, decreasing = decreasing)[1:n]
  return(table$GeneName[ind_diff])
}


Next, we define a function that uses this gene selection to subset the normalised counts for each population:

use_select <- function(sce, psm, sce_sm, select) {
  counts_psm <- assay(sce_psm, "BASiCS_norm")[select,]
  counts_sm <- assay(sce_sm, "BASiCS_norm")[select,]
  rownames(counts_psm) <- rownames(counts_sm) <-
    rowData(sce_sm)[select, "external_gene_name"]
  list (psm = counts_psm, sm = counts_sm)
}


Next, we define a function that uses the circlize package to create a color scale for the heatmaps:

make_colorscale_counts <- function(counts_sm, counts_psm) {
  colorRamp2(
    breaks = seq(0,
      log10(max(c(as.numeric(counts_sm), as.numeric(counts_psm)) + 1)),
      length.out = 20
    ),
    colors = viridis(20)
  )
}


Next, we define a function that uses the circlize package to create a color scale for the side annotations displaying the mean expression values in each group:

make_colorscale_mu <- function(log_mu_sm, log_mu_psm) {
  colorRamp2(
    breaks = seq(0, max(c(log_mu_sm, log_mu_psm)), length.out = 20),
    colors = viridis(20, option = "A", direction = 1)
  )
}


Then, we define a function that uses the ComplexHeatmap package to create a single heatmap, with the normalised counts for a single population, featuring the mean expression values for each gene as a side annotation:

make_heatmap <- function(counts, col, log_mu, mu_col, title) {
  Heatmap(
    log10(as.matrix(counts) + 1),
    col = col,
    right_annotation = rowAnnotation(
      'log(mu)' = log_mu,
      col = list('log(mu)' = mu_col)
    ),
    name = "log10(count + 1)",
    column_title = title,
    show_column_names = FALSE,
    cluster_columns = FALSE,
    cluster_rows = FALSE,
    row_names_gp = gpar(fontsize = 6)
  )
}


Finally, we define a function that uses the functions we defined so far to create a combined heatmap showing the normalised counts for both populations, with mean expression values as side annotations:

plot_heatmap <- function(table, condition, decreasing = TRUE) {
  select <- select_top_n(table_de, condition, decreasing = decreasing)
  counts_sub <- use_select(sce, sce_psm, sce_sm, select)
  counts_sm <- counts_sub$sm
  counts_psm <- counts_sub$psm

  ## Subset table of DE results to extract mean estimates
  match_order <- match(rownames (counts_sm), table_de$external_gene_name)
  table_de_selected <- table_de[match_order,]

  col <- make_colorscale_counts(counts_sm, counts_psm)
  log_mu_sm <- log10(table_de_selected$Mean1)
  log_mu_psm <- log10(table_de_selected$Mean2)

  mu_col <- make_colorscale_mu(log_mu_sm, log_mu_psm)

  h <- make_heatmap(counts_sm, col, log_mu_sm, mu_col, "Somitic cells") +
      make_heatmap(counts_psm, col, log_mu_psm, mu_col, "Pre-somitic cells")
  draw(h)
}


First, we show genes up-regulated in somitic cells:

plot_heatmap(table_de, "PSM+")


We can create a similar plot for genes up-regulated in SM cells:

plot_heatmap(table_de, "SM+")


Finally, we show non-differentially expressed genes:

plot_heatmap(table_de, "NoDiff", decreasing = FALSE)


While several computational tools exist to perform differential mean expression analysis using scRNAseq data, ^39^ the key focus of *BASiCS* is to perform differential variability testing: identifying changes in transcriptional variability between the groups of cells. To avoid the confounding between mean and over-dispersion, we recommend to use residual over-dispersion parameters *ε*_*i*_ as input to this analysis.

**Figure 13.**
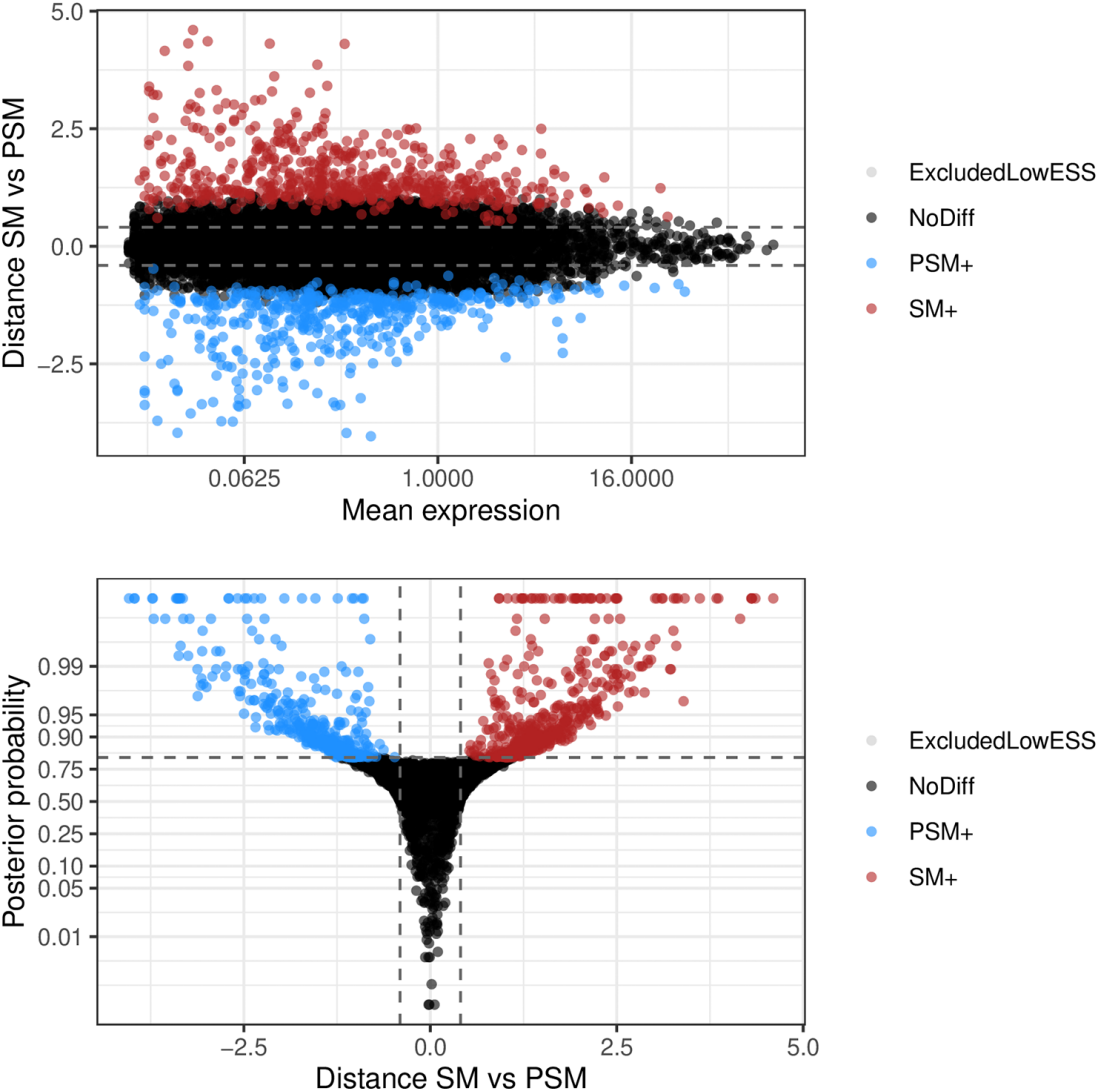
Upper panel presents the mean-difference plot associated to the differential residual over-dispersion test between somitic and pre-somitic cells. Differences of residual over-dispersion in somitic cells relative to pre-somitic mesoderm cells are plotted against average expression estimates combined across both groups of cells. Bottom panel presents the volcano plot associated to the same test. Differences of residual over-dispersion in somitic cells relative to pre-somitic cells are plotted against their associated tail posterior probabilities. Colour indicates the differential expression status for each gene, including a label to identify genes that were excluded from differential expression test due to low ESS.

We can now visualise the changes in residual over-dispersion between somitic and pre-somitic mesoderm cells in the form of a MA-plot ( Figure 13). In this visualisation, the difference between the posterior medians of the residual over-dispersion parameters *ε*_*i*_ are shown on the y-axis. Epsilon values for genes that are not expressed in at least two cells per condition are marked as NA and are therefore not displayed.

p1 <- BASiCS_PlotDE(test_de, Parameters = "ResDisp", Plots = "MA")
p2 <- BASiCS_PlotDE(test_de, Parameters = "ResDisp", Plots = "Volcano",
  TransLogit = TRUE)
p1/p2


**Figure 14.**
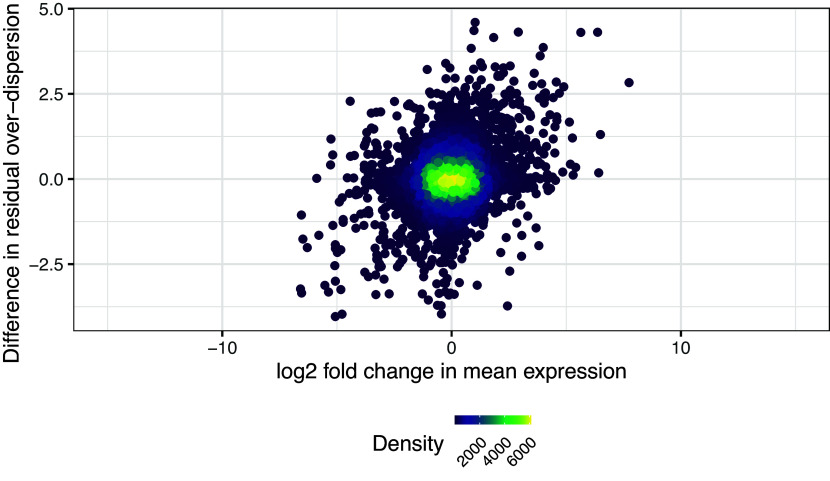
Difference in residual over-dispersion against log2 fold change in mean expression.

While one could focus on the sets of gene that show significant changes in residual over-dispersion, here we want to highlight how to analyse changes in mean expression in parallel to changes in variability. For this, we first combine the results of the differential mean expression and the differential residual over-dispersion test. These are independent analyses, given that changes in residual over-dispersion are not confounded with changes in mean expression, as shown in Figure 14.

## create list of generic plot parameters to use across a few plots
plot_params <- list(
  geom_pointdensity(na.rm = TRUE),
  scale_colour_viridis(name = "Density"),
  theme(
    # text = element_text(size = rel(3)),
    legend.position = "bottom",
    legend.text = element_text(angle = 45, size = 8, hjust = 1, vjust = 1),
    legend.key.size = unit(0.018, "npc")
  )
)
## plot log2FC against difference of residual over-dispersion
ggplot(table_de) +
  aes(MeanLog2FC, ResDispDistance) +
  plot_params +
  xlim(-15, 15) +
  labs(
    x = "log2 fold change in mean expression",
    y = "Difference in residual over-dispersion"
  )


**Figure 15.**
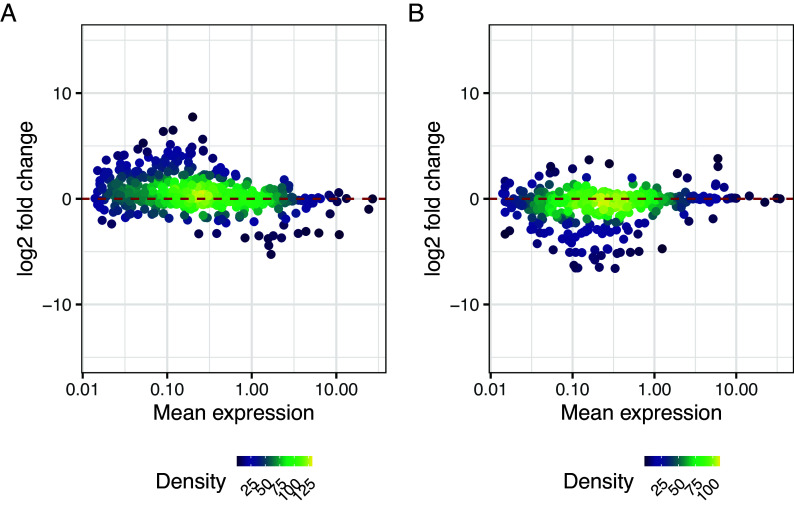
log _2_ change in expression against log mean expression for genes with higher residual over-dispersion in somitic (A) cells and pre-somitic mesoderm (B) cells. Dashed red lines represent a log fold change of zero, meaning no change in average expression.

Genes with significant changes in residual over-dispersion often have similar levels of mean expression, as seen in Figure 15. However, they may have a different proportion of zero counts in the two cell populations, indicating a more bursting expression pattern, or similar proportion of zero counts but more varying expression levels. We will now explore ways to identify genes in each of these categories.

## mean expression vs log2FC for genes with higher residual over-dispersion in
## somitic cells
g1 <- ggplot(
    table_de[table_de$ResultDiffResDisp == "SM+",]
  ) +
  aes(MeanOverall, MeanLog2FC) +
  plot_params +
  ylim(-15, 15) +
  scale_x_log10() +
  labs(x = "Mean expression", y = "log2 fold change") +
  geom_hline(
    yintercept = 0, colour = "firebrick", linetype = "dashed"
  )

## mean expression vs log2FC for genes with higher residual over-dispersion in
## pre-somitic cells
g2 <- ggplot(
    table_de[table_de$ResultDiffResDisp == "PSM+",]
  ) +
  aes(MeanOverall, MeanLog2FC) +
  plot_params +
  ylim(-15, 15) +
  scale_x_log10() +
  labs(x = "Mean expression", y = "log2 fold change") +
  geom_hline(
    yintercept = 0, colour = "firebrick", linetype = "dashed"
  )
g1 + g2 + plot_annotation(tag_levels = "A")


Similarly to analysis of differential expression, it is useful to visualise the results of differential variability tests in order to appraise the quality of the results, and to identify systematic patterns among the genes identified. One useful way to do this is by examining the normalised counts on a gene-by-gene basis. To do this, we first add some gene-level summary statistic about the normalised counts to the table_de object, and then define a function that selects the normalised counts for a given gene, and then reshapes them into a form suitable for plotting.

## merge with some summary statistics about genes
gene_tests <- data.frame(
  "GeneName" = rownames(sce_sm),
  "ExpPropSM" = rowMeans(assay(sce_sm, "BASiCS_norm") > 0),
  "ExpPropPSM" = rowMeans(assay(sce_psm, "BASiCS_norm") > 0)
)

table_de_genetests <- merge(table_de, gene_tests)
make_vg_df <- function(ind_vg) {
  table_de_vg <- table_de[ind_vg,]
  ## If more than 4 genes,
  ## pick top 4 ranked by differences in residual over-dispersion
  if (nrow(table_de_vg) > 4) {
    table_de_vg <- table_de_vg[
      order(abs(table_de_vg$ResDispDistance), decreasing = TRUE),]
    table_de_vg <- table_de_vg[1:4,]
  }
  var_genes <- table_de_vg$GeneName
  var_sm <- as.matrix(assay(sce_sm, "BASiCS_norm")[var_genes, , drop = FALSE])
  var_psm <- as.matrix(assay(sce_psm, "BASiCS_norm")[var_genes, , drop = FALSE])
  var_df <- rbind(
    data.frame(t(var_sm), Group = "SM"),
    data.frame(t(var_psm), Group = "PSM")
  )
  var_gene_names <- rowData(sce_sm)[var_genes, "external_gene_name"]
  colnames(var_df)[-ncol(var_df)] <- var_gene_names
  pivot_longer(var_df, cols = var_gene_names)
}


We can use this function to wrangle the data for a selection of genes we want to visualise. For example, all genes that are identified as differentially variable in somitic cells:

df_vg_sm <- make_vg_df(table_de_genetests$ResultDiffResDisp %in% c("SM+"))
head(df_vg_sm)
## # A tibble: 6 × 3
##   Group  name   value
##   <chr>  <chr>  <dbl>
## 1 SM     Krt19  0
## 2 SM     Hesx1  0
## 3 SM     Kdr    0
## 4 SM     Uncx   0
## 5 SM     Krt19  0
## 6 SM     Hesx1  0


We can then use this function to plot the normalised counts for genes that fill a number of criteria with respect to mean expression, expression variability, and detection levels, by defining a function that uses the make_vg_df function we just defined. This function takes a logical vector and uses it to subset the table_de, sce_sm and sce_psm objects, merges them into a form suitable for plotting, and then plots them.

## Utility function that plots logcounts of a set of genes defined by "ind_vg"
plot_vg <- function(ind_vg) {
  df <- make_vg_df(ind_vg)
  ggplot (df, aes(x = name, y = value + 1, colour = Group)) +
    geom_violin(width = 0.8, position = position_dodge(width = 0.8)) +
    geom_point(
      position = position_jitterdodge(jitter.width = 0.2, dodge.width = 0.8),
      shape = 16,
      alpha = 0.2
    ) +
    scale_x_discrete(guide = guide_axis(angle = 45)) +
    scale_y_log10() +
    labs(x = "Gene", y = "Denoised count + 1") +
    scale_colour_brewer(palette = "Set1")
}


First, we select genes that are more variable in somitic cells than in pre-somitic mesoderm cells, but which are not differentially expressed. Furthermore, we narrow our selection to genes with similar levels of non-zero expression in both populations, and which are expressed in at least 50% of cells within each population. These criteria are designed to select genes that likely have a largely unimodal expression pattern, simply having higher cell-to-cell variability in somitic cells. We then plot the normalised counts for these genes using the function we defined above, and save it to a variable called g1 for display later.

ind_vg_unimodal_sm <- table_de_genetests$ResultDiffResDisp %in% c("SM+") &
  table_de_genetests$ResultDiffMean %in% c("NoDiff") &
  abs(
    table_de_genetests$ExpPropSM - table_de_genetests$ExpPropPSM
  ) < 0.05 &
  pmin(
    table_de_genetests$ExpPropSM, table_de_genetests$ExpPropPSM
  ) > 0.99
g1 <- plot_vg(ind_vg_unimodal_sm) +
    theme (legend.position = "none")


Next, we select genes that are more variable in somitic cells than in pre-somitic mesoderm cells, but which are not differentially expressed. Furthermore, we narrow our selection to genes with different levels of non-zero expression in both populations, and which are expressed in at least 50% of cells within one or more of the populations. These are genes that could be described as having a more bursting pattern of expression in somitic cells. We then plot the normalised counts for these genes, and again save the plot object for display later.

ind_vg_bursting_sm <- table_de_genetests$ResultDiffResDisp %in% c("SM+") &
  table_de_genetests$ResultDiffMean %in% c("NoDiff") &
  abs(
    table_de_genetests$ExpPropSM - table_de_genetests$ExpPropPSM
  ) > 0.1 &
  pmax(
    table_de_genetests$ExpPropSM, table_de_genetests$ExpPropPSM
  ) > 0.75
g2 <- plot_vg(ind_vg_bursting_sm)


Now, we use the same selection criteria, plotting first for genes that are largely unimodal, with a high cell-to-cell variability in pre-somitic cells, and then for genes that have a more bursting expression pattern in pre-somitic cells:

ind_vg_unimodal_psm <- table_de_genetests$ResultDiffResDisp %in% c("PSM+") &
  table_de_genetests$ResultDiffMean %in% c("NoDiff") &
  abs(
    table_de_genetests$ExpPropSM - table_de_genetests$ExpPropPSM
  ) < 0.1 &
  pmin(
    table_de_genetests$ExpPropSM, table_de_genetests$ExpPropPSM
  ) > 0.75
g3 <- plot_vg(ind_vg_unimodal_psm) +
    theme(legend.position = "none")

ind_vg_bursting_psm <- table_de_genetests$ResultDiffResDisp %in% c("PSM+") &
  table_de_genetests$ResultDiffMean %in% c("NoDiff") &
  abs(
    table_de_genetests$ExpPropSM - table_de_genetests$ExpPropPSM
  ) > 0.10 &
  pmax(
    table_de_genetests$ExpPropSM, table_de_genetests$ExpPropPSM
  ) > 0.50
g4 <- plot_vg(ind_vg_bursting_psm)


Now that we have created four violin plots of the various types of differentially variable gene that we have defined, we can combine the plots together to visualise them simultaneously:

(g1 + g2) / (g3 + g4) + plot_annotation(tag_levels = "A")


**Figure 16.**
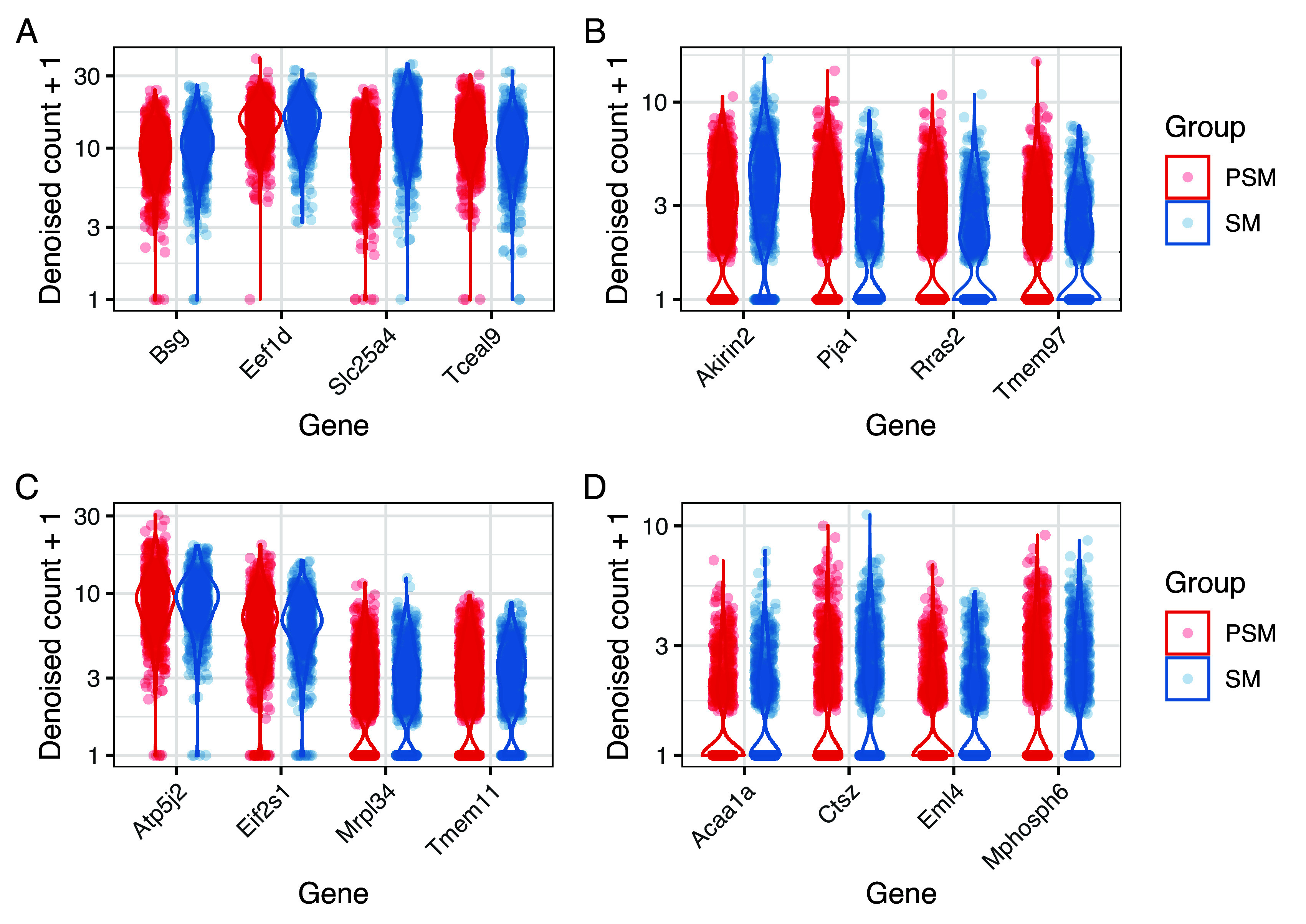
Violin plots of denoised counts. A: Four genes with higher residual over-dispersion in somitic cells, and similar levels of detection in pre-somitic and somitic populations. B: Four genes with higher residual over-dispersion in somitic cells and different levels of detection in somitic and pre-somitic cells. C: Four genes with higher residual over-dispersion in pre-somitic cells and similar levels of detection in somitic and pre-somitic cells. D: Four genes with higher residual over-dispersion in pre-somitic cells and different levels of detection in somitic and pre-somitic cells.

Figure 16 shows denoised counts for genes with significant differences in residual over-dispersion, with each panel showing a different type of expression patterns. Exploration of such patterns is important component of any analysis of differential variability, and should be undertaken with care. Figure 16B and 16D show genes with differing levels of detection in both populations, as well as higher levels of residual over-dispersion in somitic or pre-somitic mesoderm cells (B and D, respectively). Thus, these genes may represent those with a more bursty expression pattern in one of the cell population. They may also represent genes that are markers of extrinsic variability, for example cell sub-populations that differ in abundance between the cell populations in question. In contrast, Figure 16A and 16C show genes with similar levels of detection in both populations, as well as higher levels of residual over-dispersion in somitic or pre-somitic mesoderm cells (A and C, respectively). These cases are likely driven by more tight regulation, rather than transcriptional burst or sub-population structure.

## Discussion

In this article, we have explored the research questions that *BASiCS* seeks to resolve — chiefly, robustly quantifying average and variability in expression in cell populations. We have outlined the appropriate quality control and data visualisation steps to apply when undertaking an analysis using *BASiCS* in order to ensure high quality input data. We have also outlined the steps needed to use *BASiCS* to quantify biological variability, identify highly variable genes, and normalise scRNAseq data from a single population. We have also provided a limited comparison of the results of these analyses using *BASiCS* and the result of similar analyses using *scran* . Furthermore, we have demonstrated functions within *BASiCS* that allow users to ensure the MCMC used in *BASiCS* has converged and produced adequate sample sizes. Finally, we have demonstrated the use of *BASiCS* to robustly identify differentially expressed genes, in terms of mean expression and in terms of biological variability.

Further challenges exist in analysing scRNAseq data. ^10^^,^^27^ One area for future work is to study how post-selection inference may affect the performance of *BASiCS* . For example, Ref. 63 have developed a framework for addressing inflated type I errors when studying differences in means between groups of cells identified via hierarchical clustering, by splitting counts into “train” and “test” sets. Another important challenge for *BASiCS* is computational efficiency. The number of cells profiled in scRNAseq experiments has scaled exponentially since the development of the technology. ^64^ Given that *BASiCS* requires computationally intensive MCMC sampling to estimate the posterior distribution, it becomes computationally intractable to analyse data from very large numbers of cells. Alternative inference schemes such as variational inference, maximum a posteriori estimation, or Hamiltonian Monte Carlo may be useful in this context. Advanced users may wish to use the BASiCStan package to test these alternative inference schemes. This also provides access to the model diagnostics, facilities for running multiple chains, and posterior summaries provided by Stan, ^65^ while also being fully compatible with the workflow described in this manuscript via the function Stan2BASiCS, that converts the output of the Stan inference procedure to the type of output generated by BASiCS_MCMC. However, we note that the Hamiltonian Monte Carlo inference method provided by Stan is more computationally intensive than our default implementation. Furthermore, the facster approximations provided by Stan, namely scalable variational inference and maximum a posteriori estimation, are often unstable and less accurate.

Finally, we also anticipate potential extensions of *BASiCS* to account for the more complex experimental designs. For example, in cohort studies where cells are extracted from multiple individuals, the hierarchical model could be expanded to quantify both for intra- and inter-individual transcriptional variability. As spatial transcriptomic technologies mature, ^66^ Bayesian hierarchical models such as the one implemented in *BASiCS* could also be designed to incorporate spatial (and, potentially, temporal) structure (e.g. Ref. 67). We intend to update this workflow as the field evolves, and as we address the issues and challenges outlined here.

## Data availability

The data used in this article are available for download on EBI ArrayExpress under accession number E-MTAB-4888. The MCMC chains used to generate this article can be found in Zenodo under the DOI 10.5281/zenodo.10251224.

## Software availability

All software used in this workflow is available as part of Bioconductor 3.18 at: https://bioconductor.org/packages/3.18.

The source code for BASiCS, along with facilities contributing and reporting bugs is available at: https://github.com/catavallejos/BASiCS/.

The source code used for this manuscript is available at: https://github.com/VallejosGroup/BASiCSWorkflow/, and as archived source code at time of publication: https://zenodo.org/doi/10.5281/zenodo.5224614. ^26^

License: GPL-2.0

### Reproducibility

The following software versions were used throughout this workflow:

• **R version**: R version 4.3.2 (2023-10-3)
• **Bioconductor version**: 3.18
• **R packages**:
  - BASiCS 2.14.0
  - scran 1.30.10
  - scater 1.30.1

Version numbers for all remaining packages are available in the Session Info section.

A Docker image containing all software requirements is available at Docker hub. This image can be downloaded using the command docker pull alanocallaghan/basicsworkflow2020-docker:0.5.3.

## Session Info



sessionInfo()
## R version 4.3.2 (2023-10-31)
## Platform: x86_64-pc-linux-gnu (64-bit)
## Running under: Ubuntu 22.04.3 LTS
##
## Matrix products: default
## BLAS: /usr/lib/x86_64-linux-gnu/openblas-pthread/libblas.so.3
## LAPACK: /usr/lib/x86_64-linux-gnu/openblas-pthread/libopenblasp-r0.3.20.so; LAPACK version 3.10.0
##
## locale:
##  [1] LC_CTYPE=en_US.UTF-8       LC_NUMERIC=C
##  [3] LC_TIME=en_US.UTF-8        LC_COLLATE=en_US.UTF-8
##  [5] LC_MONETARY=en_US.UTF-8    LC_MESSAGES=en_US.UTF-8
##  [7] LC_PAPER=en_US.UTF-8       LC_NAME=C
##  [9] LC_ADDRESS=C               LC_TELEPHONE=C
## [11] LC_MEASUREMENT=en_US.UTF-8 LC_IDENTIFICATION=C
##
## time zone: Etc/UTC
## tzcode source: system (glibc)
##
## attached base packages:
## [1] grid     stats4    stats    graphics    grDevices utils    datasets
## [8] methods  base
##
## other attached packages:
##  [1] tidyr_1.3.0                        RColorBrewer_1.1-3
##  [3] circlize_0.4.15                    viridis_0.6.4
##  [5] viridisLite_0.4.2                  ComplexHeatmap_2.18.0
##  [7] patchwork_1.1.3                    ggpointdensity_0.1.0
##  [9] BASiCS_2.14.0                      scran_1.30.0
## [11] scater_1.30.1                      scuttle_1.12.0
## [13] SingleCellExperiment_1.24.0        SummarizedExperiment_1.32.0
## [15] Biobase_2.62.0                     GenomicRanges_1.54.1
## [17] GenomeInfoDb_1.38.1                IRanges_2.36.0
## [19] S4Vectors_0.40.2                   BiocGenerics_0.48.1
## [21] MatrixGenerics_1.14.0              matrixStats_1.1.0
##  23] ggplot2_3.4.4                      knitr_1.45
## [25] BiocStyle_2.30.0
##
## loaded via a namespace (and not attached):
##   [1] rstudioapi_0.15.0                  tensorA_0.36.2
##   [3] shape_1.4.6                        magrittr_2.0.3
##   [5] magick_2.8.1                       ggbeeswarm_0.7.2
##   [7] farver_2.1.1                       rmarkdown_2.25
##   [9] fs_1.6.3                           GlobalOptions_0.1.2
##  [11] zlibbioc_1.48.0                    vctrs_0.6.4
##  [13] Cairo_1.6-2                        DelayedMatrixStats_1.24.0
##  [15] RCurl_1.98-1.13                    tinytex_0.48
##  [17] usethis_2.2.2                      htmltools_0.5.6.1
##  [19] S4Arrays_1.2.0                     distributional_0.3.2
##  [21] BiocNeighbors_1.20.0               SparseArray_1.2.2
##  [23] plyr_1.8.9                         igraph_1.5.1
##  [25] mime_0.12                          lifecycle_1.0.3
##  [27] iterators_1.0.14                   pkgconfig_2.0.3
##  [29] rsvd_1.0.5                         Matrix_1.6-1.1
##  [31] R6_2.5.1                           fastmap_1.1.1
##  [33] GenomeInfoDbData_1.2.11            shiny_1.7.5.1
##  [35] clue_0.3-65                        digest_0.6.33
##  [37] colorspace_2.1-0                   dqrng_0.3.2
##  [39] irlba_2.3.5.1                      beachmat_2.18.0
##  [41] labeling_0.4.3                     fansi_1.0.5
##  [43] httr_1.4.7                         abind_1.4-5
##  [45] compiler_4.3.2                     withr_2.5.1
##  [47] doParallel_1.0.17                  backports_1.4.1
##  [49] BiocParallel_1.36.0                hexbin_1.28.3
##  [51] highr_0.10                         MASS_7.3-60
##  [53] DelayedArray_0.28.0                rjson_0.2.21
##  [55] bluster_1.12.0                     tools_4.3.2
##  [57] vipor_0.4.5                        beeswarm_0.4.0
##  [59] httpuv_1.6.12                      glue_1.6.2
##  [61] promises_1.2.1                     checkmate_2.3.0
##  [63] cluster_2.1.4                      reshape2_1.4.4
##  [65] generics_0.1.3                     gtable_0.3.4
##  [67] BiocSingular_1.18.0                ScaledMatrix_1.10.0
##  [69] metapod_1.10.0                     utf8_1.2.4
##  [71] XVector_0.42.0                     stringr_1.5.0
##  [73] ggrepel_0.9.4                      foreach_1.5.2
##  [75] pillar_1.9.0                       ggExtra_0.10.1
##  [77] limma_3.58.1                       posterior_1.5.0
##  [79] later_1.3.1                        BiocWorkflowTools_1.28.0
##  [81] dplyr_1.1.4                        lattice_0.22-5
##  [83] tidyselect_1.2.0                   locfit_1.5-9.8
##  [85] miniUI_0.1.1.1                     git2r_0.33.0
##  [87] gridExtra_2.3                      bookdown_0.36
##  [89] edgeR_4.0.2                        xfun_0.40
##  [91] statmod_1.5.0                      stringi_1.7.12
##  [93] yaml_2.3.7                         evaluate_0.22
##  [95] codetools_0.2-19                   tibble_3.2.1
##  [97] BiocManager_1.30.22                cli_3.6.1
##  [99] xtable_1.8-4                       munsell_0.5.0
## [101] Rcpp_1.0.11                        coda_0.19-4
## [103] png_0.1-8                          parallel_4.3.2
## [105] ellipsis_0.3.2                     assertthat_0.2.1
## [107] sparseMatrixStats_1.14.0           bitops_1.0-7
## [109] scales_1.3.0                       purrr_1.0.2
## [111] crayon_1.5.2                       GetoptLong_1.0.5
## [113] rlang_1.1.1                        cowplot_1.1.1

