# Supplementary material for: BASiCS workflow: a step-by-step analysis of expression variability using single cell RNA sequencing data
Source: F1000Res. 2024 May 7;11:59. Originally published 2022 Jan 18. [Version 2] doi: 10.12688/f1000research.74416.2 (PMC11109695; doi:10.12688/f1000research.74416.2)
Supplement: Peer review history

# Reviewer report: Reviewer response for version 2

**DOI:** 10.5256/f1000research.162017.r274782

**Published:** 2024-05-09

**Authors:** Oliver M. Crook

**Affiliations:** 1 Department of Statistics, University of Oxford, Oxford, UK

I greatly appreciate the updated article and, from my perspective, is more focused towards its target audience. The work here represent a significant effort on the author's part to ensure their work is reproducible and, importantly, useable to their intended community. 

 I have a few (extremely minor) comments.

 1) In the description of `Thin`, I would suggest something like the following definition: A value r such that the sequence of draws indexed by 1 + rn, for integer values of n, are stored (e.g. reduce the size by roughly 1/r). I think the current definition is incorrect e.g. for a Thin of 4, you wish to store values 1, 5, 9,... ? Not just the 4th? Perhaps I am mis-undertanding 

 2) I would be a bit more pedantic (sorry!) about \mu_i and \delta_i. You want carefully distinguish different way this could be (mis)-interpreted. The main point is to be really careful about independence between \mu and \delta vs independence between \mu_j and \mu_i. For example, the following options are all possible given some of the descriptions but I think only two (A and C) are what you intend?

 A) The *pair *(\mu_i, \delta_i) has a joint prior p(\mu_i, \delta_i) (e.g. there is a single global prior for all i)

 B) The *pair **s*** (\mu_i, \delta_i), indexed by i, have joint priors p_i (e.g. each pair has a seperate prior from the same family)

 C) independent univariate log-normal priors are assigned to *each of \mu_i *and to *\delta_i*

 D) a joint prior is applied over all \mu_i and a joint prior over all \delta_i

 3) Increasing the value of `EpsilonM`. I think I bit more of a hint as to what sort of range of values one might consider would be useful. 

 4) Bayesian inference is quite a nice solution to post-selection inference because you can condition on everything but that requires you to model any clustering process heirachically. I think cut a model would also be appropriate - again more modelling for a another time!

Are the conclusions about the tool and its performance adequately supported by the findings presented in the article?

Yes

Is the rationale for developing the new software tool clearly explained?

Yes

Is the description of the software tool technically sound?

Yes

Are sufficient details of the code, methods and analysis (if applicable) provided to allow replication of the software development and its use by others?

Yes

Is sufficient information provided to allow interpretation of the expected output datasets and any results generated using the tool?

Yes

Reviewer Expertise:

Biostatistics, Bayesian methodology, R, data-intensive biology

I confirm that I have read this submission and believe that I have an appropriate level of expertise to confirm that it is of an acceptable scientific standard.

# Reviewer report: Reviewer response for version 1

**DOI:** 10.5256/f1000research.78169.r128930

**Published:** 2022-04-19

**Authors:** Michel S Naslavsky

**Affiliations:** 1 Human Genome and Stem Cell Research Center, University of São Paulo, São Paulo, Brazil

O'Callaghan and colleagues present an approach to analyze single-cell RNA sequencing data in a stepwise manner. This is very useful both to readers and to students engaged in learning applied bioinformatics. As a user (non-developer, non-expert in RNA analysis tool evaluation), my contribution will be from a different perspective from the other reviewers who made excellent and detailed comments on this manuscript.

 1) Introduction, 2nd paragraph: “Moreover, these variability estimates can also be inflated by the technical noise that is typically observed in scRNA-seq data”

**Comment: Please illustrate with examples of technical noise as presented above for extrinsic and intrinsic noise, e.g. RNA differential degradation, ligation bias, etc.**

 2) Introduction, 3rd paragraph: “However, despite the benefits associated to the use of spike-ins and UMIs, these are not available for all scRNA-seq protocols.”

**Comment: Please provide a reason - something like “due to the very nature of the assay, which isolates library prep from external spike-ins and uses UMIs to map single-cell libraries…” or that spike-ins added to the library after pooling limit the full advantage of using them across single-cell populations.**

 3) Methods, 2nd paragraph: “Mean parameters *μi* quantify the overall expression for each gene *i* across the cell population under study.” 

**Comment:** **Please define cell population as sample (all cells within a scRNA-seq assay) or post-processing UMAP, t-SNE, or similar clustering grouping. I understood the latter, but readers should be certain, and we need to understand which step these parameters refer to.**

 4) After Figure 5: “These thresholds can vary across datasets and should be informed by gene-specific QC metrics such as those shown in Figure 5 as well as prior knowledge about the cell types and conditions being studied, where available”.

**Comment:** **I understand this is not the intent of this study, but if possible include some rationale behind thresholds for QCing gene exclusion based on types and conditions. Maybe bringing the choice for the example explored here.**

 5) After spike-in calculation step: “To update the sce_naive and sce_active objects, the user must create a data.frame whose first column contains the spike-in labels (e.g. ERCC-00130) and whose second column contains the number of molecules calculated above. We add this as row metadata for altExp (sce_naive) and altExp (sce_active).”

**Comment:** **By ‘update’, do the authors mean ‘update after normalisation with spike-in’? If so, please specify.**

 7) MCMC diagnostics

**Comment: I am by far not an expert on the analysis, but the authors were very successful in explaining the protocol in a stepwise manner. I would kindly ask if they can add the reason why this is needed: “to ensure that comparisons of gene expression are not random” or something in that direction. Readers will appreciate it.**

 8) Figures 15 and 16

**Comment: These explanations are important to establish a convincing argument for using this workflow. Authors can explore further the ‘tight regulation’ versus ‘transcriptional burst’ or ‘sub-population structure’ scenarios with other examples (either from the literature or running an orthogonal analysis on larger scRNA-seq datasets of CD4+ T cells.**

Are the conclusions about the tool and its performance adequately supported by the findings presented in the article?

Yes

Is the rationale for developing the new software tool clearly explained?

Yes

Is the description of the software tool technically sound?

Yes

Are sufficient details of the code, methods and analysis (if applicable) provided to allow replication of the software development and its use by others?

Yes

Is sufficient information provided to allow interpretation of the expected output datasets and any results generated using the tool?

Yes

Reviewer Expertise:

Population and medical genomics

I confirm that I have read this submission and believe that I have an appropriate level of expertise to confirm that it is of an acceptable scientific standard, however I have significant reservations, as outlined above.

# Response

**Published:** 2024-01-29

**Authors:** Alan O'Callaghan

**Affiliations:** MRC Human Genetics Unit, UK

O'Callaghan and colleagues present an approach to analyse single-cell RNA sequencing data in a stepwise manner. This is very useful both to readers and to students engaged in learning applied bioinformatics. As a user (non-developer, non-expert in RNA analysis tool evaluation), my contribution will be from a different perspective from the other reviewers who made excellent and detailed comments on this manuscript.

**Authors’ response:**

 We are grateful for the positive comment provided by Dr Naslavsky about the usefulness of our workflow. Many thanks for highlighting the issues listed below, we believe that the clarity of our manuscript has substantially improved after addressing these comments. Edits to the main text are highlighted in *italics*. 

**Reviewer comment:**

 1) Introduction, 2nd paragraph: “Moreover, these variability estimates can also be inflated by the technical noise that is typically observed in scRNA-seq data”

 Comment: Please illustrate with examples of technical noise as presented above for extrinsic and intrinsic noise, e.g. RNA differential degradation, ligation bias, etc.

**Authors’ response:**

 We have edited the Introduction to provide additional information about potential sources for technical noise. The following text has been added at the end of the second paragraph:

*This technical noise relates to systematic differences between cells that may be introduced by the data generating process (e.g., due to differences in dilution or sequencing depth) [@Vallejos2017].*

**Reviewer comment:**

 2) Introduction, 3rd paragraph: “However, despite the benefits associated to the use of spike-ins and UMIs, these are not available for all scRNA-seq protocols.”

 Comment: Please provide a reason - something like “due to the very nature of the assay, which isolates library prep from external spike-ins and uses UMIs to map single-cell libraries…” or that spike-ins added to the library after pooling limit the full advantage of using them across single-cell populations.

**Authors’ response:**

 We have added the following text to note the difficulties in using UMIs and spike-ins with some assays. For more detail, see https://www.ncbi.nlm.nih.gov/pmc/articles/PMC4823857/.

*In particular, spike-ins are of limited use in droplet-based protocols, as spike-ins can only be added to the reagent mixture in a known concentration, and the exact quantity in each droplet necessarily remains unknown [@Bacher2016].*

**Reviewer comment:**

 3) Methods, 2nd paragraph: “Mean parameters μi quantify the overall expression for each gene i across the cell population under study.” 

 Comment: Please define cell population as sample (all cells within a scRNA-seq assay) or post-processing UMAP, t-SNE, or similar clustering grouping. I understood the latter, but readers should be certain, and we need to understand which step these parameters refer to.

**Authors’ response:**

 In order to clarify this, we have edited the Introduction to describe the experimental designs in which the BASiCS model can be applied: 

The model was initially motivated by supervised experimental designs in which experimental conditions correspond to groups of cells defined *a priori* (e.g. selected cell types obtained through FACS sorting). However, the approach can also be used in cases where the groups of cells of interest are computationally identified through clustering. In such cases, the model does not currently address issues associated to *post-selection inference*, where the same data is analysed twice: first to perform clustering and then to compare expression profiles between the clusters [@Lahnemann2020]; this is an inherent limitation of most differential expression tools.

 Furthermore, we have now included the following text in the Methods, “BASiCS - Bayesian Analysis of Single Cell Sequencing data” subsection: 

*… within a cell population or populations under study. In this context, cell populations could correspond to groups set a priori by the experimental design (e.g. naive or stimulated CD4+ T cells in [@Martinez-jimenez2017]), or to groups of cells that were computationally identified through clustering.*

**Reviewer comment:**

 4) After Figure 5: “These thresholds can vary across datasets and should be informed by gene-specific QC metrics such as those shown in Figure 5 as well as prior knowledge about the cell types and conditions being studied, where available”.

 Comment: I understand this is not the intent of this study, but if possible include some rationale behind thresholds for QCing gene exclusion based on types and conditions. Maybe bringing the choice for the example explored here.

**Authors’ response:**

 We have removed the comments about choosing thresholds based on types and conditions, as we feel it does not reflect the true use of these thresholds for our use case. We thank the reviewer for the note, and have replaced this an explanation of our motivation:

*This is to ensure a reliable estimate of variability, and is roughly in line with the sample size requirements for the negative binomial distribution outlined in @Lloyd-Smith2007.*

**Reviewer comment:**

 5) After spike-in calculation step: “To update the sce_naive and sce_active objects, the user must create a data.frame whose first column contains the spike-in labels (e.g. ERCC-00130) and whose second column contains the number of molecules calculated above. We add this as row metadata for altExp (sce_naive) and altExp (sce_active).”

 Comment: By ‘update’, do the authors mean ‘update after normalisation with spike-in’? If so, please specify.

**Authors’ response:**

 Apologies for the confusion. By “update” in this context, we refer to adding the relevant information about spike-ins to the sce_naive and sce_active objects. We note that the spike-in analysis workflow has been moved to the Zenodo repository.

**Reviewer comment:**

 7) MCMC diagnostics

 Comment: I am by far not an expert on the analysis, but the authors were very successful in explaining the protocol in a stepwise manner. I would kindly ask if they can add the reason why this is needed: “to ensure that comparisons of gene expression are not random” or something in that direction. Readers will appreciate it.

**Authors’ response:**

 We agree that it is important to explain the rationale behind these diagnostics; we have update the text to reflect this as follows:

*Before interpreting the estimates generated by `r Biocpkg("BASiCS")`, it is important to check the performance of the MCMC algorithm used. This algorithm used to characterise the posterior distribution is stochastic by nature, and as such its performance may vary even when used on the same dataset using the same settings. We perform quality control of the MCMC algorithm in order to ensure that the results we observe are the result of properties of the underlying data, rather than being an artefact of the stochastic algorithm used to characterise the data.*

**Reviewer comment:**

 8) Figures 15 and 16

 Comment: These explanations are important to establish a convincing argument for using this workflow. Authors can explore further the ‘tight regulation’ versus ‘transcriptional burst’ or ‘sub-population structure’ scenarios with other examples (either from the literature or running an orthogonal analysis on larger scRNA-seq datasets of CD4+ T cells.

**Authors’ response:**

 The various sources of transcriptional variability are indeed essential to justifying the use of this workflow. We note that previous work has highlighted these different aspects in detail. For example, Eling et al 2018 explored the role of population structure by generating artificially contaminated datasets. Similarly, Martinez-Jimenez 2017 showed the change in transcriptional bursting patterns relating to ageing, where older mice were found to have more stochastic gene expression patterns relative to younger mice. To clarify this point, we have briefly discussed this in the introduction:

*For example, @Eling2018 computationally explored the impact of unobserved structural heterogeneity by generating artificially contaminated datasets. In contrast, @Martinez-Jiminenez2017 showed that changes in transcriptional heterogeneity can related to aging, where older mice were shown to exhibit a more "bursting" or stochastic transcriptional pattern relative to younger mice.*

# Reviewer report: Reviewer response for version 1

**DOI:** 10.5256/f1000research.78169.r126523

**Published:** 2022-04-01

**Authors:** Andrew McDavid

**Affiliations:** 1 Department of Biostatistics and Computational Biology, University of Rochester, Rochester, NY, USA

This is an extensive workflow on applying the authors' BASiCS method to a plate-based single-cell RNAseq data. The authors discuss preparing data downloaded from an external repository, evaluation of QC metrics, interrogation of convergence of a Bayesian model, interpretation of model parameters, and comparative tests in the two sample settings. Code to generate an expansive array of figures is provided.

 All said, it's a comprehensive workflow demoing well-documented software. I do have a few concerns about how other users would apply the workflow to their data:

1. The authors demo their method on some naive CD4 cells from inbred mice - really the ideal data set to illustrate a method looking at intrinsic sources of gene heterogeneity or homogeneity. How would prospective users of BASiCS adapt it to more typical datasets from droplet-based scRNAseq, either from samples with more heterogeneity (human) or of less purified cells, where spike-ins are absent and the "horizontal integration" model must be invoked? I am left wondering:
  - a. What sort of QC would be needed?
  - b. How should the data be stratified (into fairly fine-grained cell types on the basis of unsupervised clustering of the expression vector? Though this sort of double-dipping will change the inferential properties of the model).
  - c. How can violations of the Bayesian model be detected, or of unresolved sub-population heterogeneity, indicating more clustering is needed?
  - d. Given that with K populations, we'd have K-times the output from a single run of BASiCS to interpret. How to prioritize or understand it? (Perhaps an approach like muscat uses here would be helpful? ^1^).
  - e. What techniques can aid in fitting this computationally-intensive model on larger datasets (e.g. thousands of cells)? Should we downsample cells, or apply more aggressive pre-filtering of genes? I see there's some provision to apply a "divide and conquer strategy" to fit the model on tranches of the data in parallel. How should this be used in practice?
2. The workflow is quite lengthy, enough that I found it hard to follow at times, and that I might be missing the forest for the trees. It would be nice if it could be condensed to emphasize model interpretation. A couple of suggestions on this end:
  - a. The initial vignette about data download and QC could be moved to an appendix, since it's not very specific to the BASiCS model, and there is a lot of boilerplate code, e.g. converting gene symbols. Then we could start from a binary SingleCellExperiment object.
  - b. The plotting code is quite verbose. It would be nice from a usability standpoint to have commonly-used plots included as part of the functionality of the package.
  - c. Is there some way to provide better navigation among sections of the workflow (this may not be possible via F1000?)? This combined with a paragraph in the introduction ("This case study is organized as follows...section 3.1 describes downloading data from ArrayExpression, 3.2 annotates gene symbols, 3.3 examines QC,...") would make it easier for a reader to find the relevant section of the workflow.
  - d. Or the version of this workflow that's at https://github.com/VallejosGroup/BASiCSWorkflow/ could be rendered and made available on Github, perhaps with some nice markdown features like code-folding and a floating TOC to make it easier to read.
3. The comparison between the model estimates and the scran estimates in Figure 8 does help orient someone who is not completely familiar with the BASiCS model, but it would be nice to have a brief recap of the model in the Methods section (one or two equations), with interpretations, to explain the key parameters being estimated.  

Minor comments:

1. I encountered some issues regarding the formatting of the code, which the other reviewer also remarked upon. This may be limited to the html version of the article.
  - a. newlines in `website` strings resulting in mal-formed URL.
  - b. ^ rendered in some odd font that wouldn't execute when I copied a chunk into Rstudio.
  - c. concentration.in..Mix.1.attomoles.ul was missing an `.`
2. The built-in MCMC diagnostics are nice, but I do feel like Rhat (testing within vs between-chain variance, e.g. Vehtari *et al.,* 2021 ^2^) is maybe what I want most. Is there any provision for running parallel chains?
3. It seemed that when I ran the `BASiCS_DetectHVG` function as specified, I got a couple of warning messages: "The posterior probability threshold chosen via EFDR calibration is too low. Probability threshold automatically set equal to 'ProbThresholdM'."
4. Regarding differential expression testing, could the authors comment on the modeling assumptions about the two "populations" of cells? If I had biological replicates in some conditions (e.g. distinct mice, humans, or plots of stimulated cells), how would they be used here, or does the framework require assuming null inter-replicate variability?
5. Figure 15: Caption says "log2 change in expression against against log mean expression for genes with higher residual over-dispersion in naive (A) cells and active (D) cells", but seems to refer to panels A and C. More broadly, it's not really clear what sort of conclusions we are supposed to draw from this sort of figure.

Are the conclusions about the tool and its performance adequately supported by the findings presented in the article?

Yes

Is the rationale for developing the new software tool clearly explained?

Yes

Is the description of the software tool technically sound?

Yes

Are sufficient details of the code, methods and analysis (if applicable) provided to allow replication of the software development and its use by others?

Yes

Is sufficient information provided to allow interpretation of the expected output datasets and any results generated using the tool?

Partly

Reviewer Expertise:

Bioinformatics, biostatistics, single-cell transcriptomics

I confirm that I have read this submission and believe that I have an appropriate level of expertise to confirm that it is of an acceptable scientific standard, however I have significant reservations, as outlined above.

## References

[1] : muscat detects subpopulation-specific state transitions from multi-sample multi-condition single-cell transcriptomics data. *Nature Communications* .2020;11(1) : 10.1038/s41467-020-19894-4 10.1038/s41467-020-19894-4 PMC770576033257685

[2] : Rank-Normalization, Folding, and Localization: An Improved Rˆ for Assessing Convergence of MCMC (with Discussion). *Bayesian Analysis* .2021;16(2) : 10.1214/20-BA1221 10.1214/20-BA1221

# Response

**Published:** 2024-01-29

**Authors:** Alan O'Callaghan

**Affiliations:** MRC Human Genetics Unit, UK

We thank the reviewer for their positive assessment of our manuscript, and the detailed comments, which we have used to revise and improve the workflow. Edits to the main text are highlighted in *italics*. 

**Reviewer comment:**

 The authors demo their method on some naive CD4 cells from inbred mice - really the ideal data set to illustrate a method looking at intrinsic sources of gene heterogeneity or homogeneity. How would prospective users of BASiCS adapt it to more typical datasets from droplet-based scRNAseq, either from samples with more heterogeneity (human) or of less purified cells, where spike-ins are absent and the "horizontal integration" model must be invoked? I am left wondering:

         a. What sort of QC would be needed?

         b. How should the data be stratified (into fairly fine-grained cell types on the basis of unsupervised clustering of the expression vector? Though this sort of double-dipping will change the inferential properties of the model).

         c. How can violations of the Bayesian model be detected, or of unresolved sub-population heterogeneity, indicating more clustering is needed?

         d. Given that with K populations, we'd have K-times the output from a single run of BASiCS to interpret. How to prioritize or understand it? (Perhaps an approach like muscat uses here would be helpful?1).

         e. What techniques can aid in fitting this computationally-intensive model on larger datasets (e.g. thousands of cells)? Should we downsample cells, or apply more aggressive pre-filtering of genes? I see there's some provision to apply a "divide and conquer strategy" to fit the model on tranches of the data in parallel. How should this be used in practice?

**Authors’ response:**

1. We thank the reviewer for this note. Due to its popularity, we have therefore modified the workflow to examine droplet-based data instead. The original analysis of CD4 T cells is now provided in an additional repository hosted on Zenodo. Furthermore, to improve the flow of the manuscript, we have removed data pre-processing and quality control to the same Zenodo repository, as we feel that this topic has been covered in detail by previous work (Lun *et al.* 2016, https://f1000research.com/articles/5-2122 , Amezquita *et al.* 2019 https://doi.org/10.1038/s41592-019-0654-x ).

 

1. Typical droplet-based datasets exhibit substantial cell-to-cell heterogeneity linked to the presence of distinct cell types or states. This needs to be accounted for before running the BASiCS model. In practice, this means that users should identify similar groups of cells (e.g. cell types) through clustering. As mentioned by Dr McDavid, this introduces a double-dipping effect where the same data is used twice: first to perform clustering and then to compare expression profiles between clusters. This effect is not yet addressed in BASiCS, but we believe it is an important area for future work. 

We have now edited the Introduction in order to clarify this:

The model was initially motivated by supervised experimental designs in which experimental conditions correspond to groups of cells defined *a priori* (e.g. selected cell types obtained through FACS sorting). However, the approach can also be used in cases where the groups of cells of interest are computationally identified through clustering. In such cases, the model does not currently address issues associated to *post-selection inference*, where the same data is analysed twice: first to perform clustering and then to compare expression profiles between the clusters [@Lahnemann2020]; this is an inherent limitation of most differential expression tools.

 Furthermore, the Discussion now highlights the importance of assessing how post-selection inference may affect the performance of BASiCS (which is beyond the scope of this manuscript):

*One area for future work is to study how post-selection inference may affect the performance of `r Biocpkg("BASiCS")`. For example, @Neufeld2022 have developed a framework for addressing inflated type I errors when studying differences in means between groups of cells identified via hierarchical clustering, by splitting counts into "train" and "test" sets.*

1. Ideally, structural heterogeneity associated with the presence of distinct cell types will be addressed before applying the BASiCS model either experimentally (via cell sorting) or computationally (via clustering). Eling et al, https://doi.org/10.1016/j.cels.2018.06.011 , explored a case in which “contamination” was present, with a subpopulation of cells that had a distinct expression profile. We have observed in previous studies that highly variable genes were enriched by markers of such subpopulations. We have added a brief note explaining this in the introduction:

*These residual over-dispersion values can be used to quantify transcriptional heterogeneity without any confounding with mean expression, though it should be noted that high residual over-dispersion may also arise due to structural heterogeneity (e.g. distinct cell sub-types or states).*

1. If the data consists of $K$ pre-specified cell groups (e.g. types or states), BASiCS can be run separately within each group (as such, omputation can be trivially parallelised if multiple cores are available). Once the algorithm has been run, the MCMC chains can be post-processed to perform e.g. differential testing. 

Currently, our decision rules for differential expression are performed using two groups. Therefore, it is more suitable to focused situations in which a handful of pairwise comparisons (hypothesis) are prioritised a priori. However, in principle the posterior distributions for gene-level parameters could be combined across multiple chains to test other hypotheses, such as an omnibus-type hypothesis (similar to the ANOVA F-test). However, this is outside the scope of this workflow.

  

1. We recently obtained very promising results about the use of more scalable algorithms for Bayesian inference (e.g. divide-and-conquer MCMC, see Bardenet et al. 2015 https://arxiv.org/abs/1505.02827 ). We plan to demonstrate the properties and practical applications of computationally scalable inference in a future manuscript (in preparation). We plan to update this workflow once our benchmarks for this new, more scalable, implementation are completed and the updated BASiCS package is available in Bioconductor. However, for the time being, we have added a brief note regarding this.

         

**Reviewer comment:**

     The workflow is quite lengthy, enough that I found it hard to follow at times, and that I might be missing the forest for the trees. It would be nice if it could be condensed to emphasise model interpretation. A couple of suggestions on this end:

         a. The initial vignette about data download and QC could be moved to an appendix, since it's not very specific to the BASiCS model, and there is a lot of boilerplate code, e.g. converting gene symbols. Then we could start from a binary SingleCellExperiment object.

         b. The plotting code is quite verbose. It would be nice from a usability standpoint to have commonly-used plots included as part of the functionality of the package.

         c. Is there some way to provide better navigation among sections of the workflow (this may not be possible via F1000?)? This combined with a paragraph in the introduction ("This case study is organised as follows...section 3.1 describes downloading data from ArrayExpression, 3.2 annotates gene symbols, 3.3 examines QC,...") would make it easier for a reader to find the relevant section of the workflow.

         d. Or the version of this workflow that's at https://github.com/VallejosGroup/BASiCSWorkflow/ could be rendered and made available on Github, perhaps with some nice markdown features like code-folding and a floating TOC to make it easier to read.

**Authors’ response:**

1. Many thanks for this suggestion. We have now moved the data preparation  steps to a separate repository for clarity, as single cell data pre-processing and quality control have been covered in detail by previous work (Lun *et al.* 2016, https://f1000research.com/articles/5-2122 , Amezquita *et al.* 2019 https://doi.org/10.1038/s41592-019-0654-x ).
2. We agree that the plotting code is quite verbose, and detracts from the flow of the manuscript. We have moved the pre-processing code to a separate repository for clarity, and where possible have simplified the code to improve the reading experience.
3. We have included a short summary of the workflow sections in the introduction, to hopefully aid in navigation.
4. Following peer review, we intend to submit a version of this workflow to Bioconductor as a Bioconductor workflow ( https://www.bioconductor.org/packages/release/BiocViews.html#___Workflow ) so that it can be regularly built as part of the Bioconductor build system.

         

**Reviewer comment: **The comparison between the model estimates and the scran estimates in Figure 8 does help orient someone who is not completely familiar with the BASiCS model, but it would be nice to have a brief recap of the model in the Methods section (one or two equations), with interpretations, to explain the key parameters being estimated.  

**Authors’ response:**

 Many thanks for this suggestion. We decided to provide a high level description of the model suitable for less technical audiences. However, we agree that further information would help to better interpret the use and output of BASiCS. Therefore, we have now updated the manuscript to include a graphical overview for the BASiCS models and the relevant parameters. This includes a graphical representation of the overall mean/overdispersion trend and how this is used to derive  residual overdispersion estimates (as deviations with respect to the overall trend). We hope this helps to clarify the links with the DM estimates provided by scran. 

**Minor comments**:

**Reviewer comment:**

 I encountered some issues regarding the formatting of the code, which the other reviewer also remarked upon. This may be limited to the html version of the article.

         a. newlines in `website` strings resulting in mal-formed URL.

         b. ^ rendered in some odd font that wouldn't execute when I copied a chunk into Rstudio.

         c. concentration.in..Mix.1.attomoles.ul was missing an `.`

**Authors’ response:**

 This is a formatting issue which should be resolved now.

          

**Reviewer comment:**

 The built-in MCMC diagnostics are nice, but I do feel like Rhat (testing within vs between-chain variance, e.g. Vehtari et al., 20212) is maybe what I want most. Is there any provision for running parallel chains?

**Authors’ response:**

 Due to the running times of the current MCMC sampler, we have not suggested users to routinely run multiple MCMC chains as part of their analyses. However, throughout the years, we have run BASiCS multiple times in a variety of settings and we have generally observed that different runs of the algorithm converge to similar values. Whilst this does not guarantee that the same would hold for a new dataset, our experience strongly suggests that the proposed diagnostic criteria are sufficient when evaluating MCMC convergence for BASiCS. Nevertheless, we have added support for the Rhat diagnostic criterion, and acknowledge that running multiple chains may be useful and we will aim to implement this in future versions of the BASiCS library. In particular, we recently obtained very promising results about the use of more scalable algorithms for Bayesian inference (e.g. divide-and-conquer MCMC, see Bardenet et al. 2015 https://arxiv.org/abs/1505.0282 ; manuscript in preparation). This will facilitate running the algorithm multiple times in a timely manner. We plan to update this workflow once our benchmarks for this new, more scalable, implementation are completed and the updated BASiCS package is available in Bioconductor.

      

**Reviewer comment:**

 It seemed that when I ran the `BASiCS_DetectHVG` function as specified, I got a couple of warning messages: "The posterior probability threshold chosen via EFDR calibration is too low. Probability threshold automatically set equal to 'ProbThresholdM'."   

**Authors’ response:**

 This message relates to the choice of a probability threshold that controls the EFDR at the chosen level. This is normal behaviour that can be controlled using the ProbThreshold argument, and we have explained it using the following note in the text:

*Performing HVG and LVG detection involves identifying a posterior probability threshold that matches a target EFDR. We perform this choice using a grid search in which EFDR is calculated for a range of different probability thresholds between 0.5 and 1. As seen in Figure \@ref(fig:efdr-plot-vg-SM), for the somitic mesoderm cells, this leads to a threshold of `r hvg@ProbThreshold` and `r lvg@ProbThreshold` for HVG and LVG detection, respectively (the value of these thresholds can be extracted from the `ProbThreshold` slot in the `hvg` and `lvg` objects, e.g. using `hvg@ProbThreshold`). For some datasets, the grid search may fail to identify a probability threshold that matches the target EFDR. In such cases, the default minimum probability threshold of $\frac{2}{3}$ is used. This default threshold value can be changed using the `ProbThreshold` to `BASiCS_DetectHVG` and `BASiCS_DetectLVG`.*

**Reviewer comment:**

 Regarding differential expression testing, could the authors comment on the modelling assumptions about the two "populations" of cells? If I had biological replicates in some conditions (e.g. distinct mice, humans, or plots of stimulated cells), how would they be used here, or does the framework require assuming null inter-replicate variability?

**Authors’ response:**

 In principle, the differential expression testing approach could be used to compare expression profiles between two arbitrary groups of cells. The typical use-case is two cell types or experimental conditions for a given cell type (naive vs stimulated, grown in two different media). Users could potentially apply the model to different biological replicates. In such cases, our over-dispersion parameters will quantify intra-replicate cell-to-cell variability and our differential expression test will quantify systematic differences between biological replicates. However, this approach does not share information across biological replicates when estimating model parameters (this can be useful in situations where a small number of cells is available per biological replicate, or when the data is sparse). Moreover, this would not scale well to cohort studies (e.g. van der Wijst et al. 2018, https://doi.org/10.1038/s41588-018-0089-9 ) where multiple biological replicates are present. We have added a brief note about this extension in the Discussion section. 

*Finally, we also anticipate potential extensions of `r Biocpkg("BASiCS")` to account for the more complex experimental designs. For example, in cohort studies where cells are extracted from multiple individuals, the hierarchical model could be expanded to quantify both for intra- and inter-individual transcriptional variability.*

**Reviewer comment:**

 Figure 15: Caption says "log2 change in expression against against log mean expression for genes with higher residual over-dispersion in naive (A) cells and active (D) cells", but seems to refer to panels A and C. More broadly, it's not really clear what sort of conclusions we are supposed to draw from this sort of figure.

**Authors’ response:**

 We agree that this figure was somewhat unclear, and we apologise for the mis-labelled panels. We have removed this figure from the revised manuscript as we feel it was not necessary.

# Reviewer report: Reviewer response for version 1

**DOI:** 10.5256/f1000research.78169.r124656

**Published:** 2022-03-08

**Authors:** Oliver M. Crook

**Affiliations:** 1 Department of Statistics, University of Oxford, Oxford, UK

**Summary:**

****

 O’Callaghan and co-authors provide a workflow for the popular method BASiCS and its extensions. This workflow is intended to display the bioconductor infrastructure of BASiCS and provide a template for analysis for others to streamline their data analysis. I find the workflow quite comprehensive and takes me through the analysis points that I would expect to see. First of all, I must confess I am not an expert in processing scRNA data and so I cannot assess whether the choices of filtering, thresholding etc are up-to-date. I will leave this assessment to other reviewers. Here, I wish to focus my review on the communication of sophisticated methods presented, the Bayesian analysis, visualizations and the code. I believe this is an extremely valuable contribution to the scRNA community, the workflow community and applied Bayesians. I would also like to thank the authors’ attention to detail, I found the article well-written and logically structured. 

 I would encourage you to update your version of R to 4.2 as the next version of Bioconductor will use this.

**General comments:**

 Whilst the method itself is presented technically in other papers, I don’t quite get a good picture of the model and its variants. Referring to the other papers I find myself confronted with lots of equations, whilst I can make my way through these details I think the intended users might struggle. The workflow might benefit from a narrative description or recipe that highlights its generative nature. A cartoon or graphic can be particularly insightful.

 As good statisticians, we should incorporate experimental design into our models. I think before the data are introduced it would be useful to have a short description of the type of experiments that could be analysed with BASiCS, when BASiCS would be useful over other approaches, when not to apply BASiCS. What sort of data would it be optimal to collect if I wish to analysed it using BASiCS? How valuable are spike-ins for example?

 I had to spend quite some time on the code chunks, working out what was going on. I think these could be decorated with useful comments so your intended user is allowed to be as lazy as possible (which is what you want so they will use your package!). This was particularly the case for the pre-processing and parameter estimation.

 There are some quite long code chunks that I felt represented fairly repetitive tasks, mostly related to plotting. If you're using ggplot2 then you can write a function which returns a ggplot object with nice defaults and you can explain that it can be customized using the ggplot principles. I think the current style is written to allow this customization but I think it's currently unexplained and perhaps working against you. 

 In the section of parameter estimation, the discussion on priors is quite brief and quite unclear currently. I’m not quite sure what the "joint prior" is here, I think your intended audience would appreciate a narrative description of these priors. The other choices of independent log Normal is also unclear. The logMean and logSd appear to be set automatically using the data - is that what you mean by Empirical Bayes here? When I dive deeper into changing the priors BASiCS_PriorParam is quite daunting. I think there are some helpful things you can do for your readers. For example, you explain whether the analysis is sensitive to the prior choice and generally how they affect the analysis. You could guide your users through a prior predictive check, in which you simulate parameters from the prior and then fake data from the likelihood. You could then show users how well calibrated their prior choices are. General advice of setting the prior or whether they should just leave these alone would be useful.

 I assume that multiple chains are run, are there any MCMC diagnostics based on running parallel chains?

 In a Bayesian analysis I expected to be able to manipulate posterior distributions or credible intervals. Maybe I missed it but can we visualize the full posterior distribution for \mu_i vs \delta_i and \mu_i vs \epsilon_i or some other parameters of interest?  

 I think I haven’t quite grasped the "variance decomposition" the authors mention into biological and technical components. This could be \delta_i and \epsilon_i, but I feel I am missing something? I do not yet think the methods section is quite clear on this point.

 For the volcano plots the posterior probability is quite bunched up, would this be better on the logit scale? Also It appears the probabilities concentrate around 0.5? Is there an intuitive explanation for this? Indeed, the distribution generated by hist(p2$data$ProbDiffMean) was quite unexpected, are these probabilities calibrated in any way?

 You mention computational challenges in the discussion, what is lost by performing maximum likelihood estimation or maximum a posteriori estimation? Perhaps the weighted likelihood bootstrap or variant could be a useful approach, if you have lots of cores.

 What are the challenges of applying this to scRNA data with spatial autocorrelation? It is common to now obtain expression data in such a context, does this additional confounding obstruct the model?

**Specific comments:**

 In the first code chunks, in `website <- " https …” I think there is an initial space which stops this from running correctly.

 Remember to include spaces after commas e.g. [a, ] rather than [a,], it is much clearer to read.

 I think it would be better to load all the packages at the beginning, I had to restart my session several times to install the packages that I realized I needed x% of the way through the workflow.

 In figure 4, the axis dimensions of the PCA misrepresents the variance explained. The ratio of axis dimensions should closely match the ratios of the variance explained so that the visualization is faithful to the dimensionality reduction. 

 Constants used in the package could be saved as global variables, it’s very easy to make a typo with these conversions.

 In the chunk starting ## Moles per microliter, I had to edit the first line of the code chunk to get it to work. Perhaps it has been malformatted?

 \log is a symbol and should always be lower-case

 In the abstract, you write "strong technical noise". I don’t quite understand what "strong" is adding here. 

 A number of concepts are left unexplained such as "joint prior", "negative binomial framework", "borrows information", "expected false discovery rate". These are all familiar concepts to someone with advanced statistical knowledge but the casual scRNA reader might find this off putting.

 Figure 12 there is a typo in the legend "\log_{1}0 -> \log_{10}".

 In Figure 12 the dendrogram is unexplained and I’m not sure what it means in this context.

Are the conclusions about the tool and its performance adequately supported by the findings presented in the article?

Yes

Is the rationale for developing the new software tool clearly explained?

Yes

Is the description of the software tool technically sound?

Yes

Are sufficient details of the code, methods and analysis (if applicable) provided to allow replication of the software development and its use by others?

Yes

Is sufficient information provided to allow interpretation of the expected output datasets and any results generated using the tool?

Yes

Reviewer Expertise:

Biostatistics, Bayesian methodology, R, data-intensive biology

I confirm that I have read this submission and believe that I have an appropriate level of expertise to confirm that it is of an acceptable scientific standard, however I have significant reservations, as outlined above.

# Response

**Published:** 2024-01-29

**Authors:** Alan O'Callaghan

**Affiliations:** MRC Human Genetics Unit, UK

We would like to thank Dr Crook for such a positive assessment of our manuscript. A detailed point-by-point response for the remaining comments is provided below. Edits to the main text are highlighted in *italics*. 

**Reviewer comment**: I would encourage you to update your version of R to 4.2 as the next version of Bioconductor will use this.

**Author’s response: **We have now updated the workflow to use R version 4.3 which is the current version suggested by Bioconductor. 

**Reviewer comment**: Whilst the method itself is presented technically in other papers, I don’t quite get a good picture of the model and its variants. Referring to the other papers I find myself confronted with lots of equations, whilst I can make my way through these details I think the intended users might struggle. The workflow might benefit from a narrative description or recipe that highlights its generative nature. A cartoon or graphic can be particularly insightful.

**Author’s response: **Many thanks for this suggestion. We have now updated the manuscript to include a graphical overview for the BASiCS model (see Figure 2 in the revised manuscript). This enables us to better introduce the role of different model parameters. In terms of the different variations of the BASiCS model, we provide a high level description for the latest version (as per Eling et al 2018) as this is what we recommend to use as default. We hope this provides a better overview of the methodology whilst ensuring that  the workflow is accessible to a wide range of users. 

**Reviewer comment**: As good statisticians, we should incorporate experimental design into our models. I think before the data are introduced it would be useful to have a short description of the type of experiments that could be analysed with BASiCS, when BASiCS would be useful over other approaches, when not to apply BASiCS. What sort of data would it be optimal to collect if I wish to analysed it using BASiCS? How valuable are spike-ins for example?

**Author’s response: **We agree a more explicit description of the experimental design assumed by BASiCS would be useful to potential issues. To address this, we included the following text in the Introduction:

The model was initially motivated by supervised experimental designs in which experimental conditions correspond to groups of cells defined *a priori* (e.g. selected cell types obtained through FACS sorting). However, the approach can also be used in cases where the groups of cells of interest are computationally identified through clustering. In such cases, the model does not currently address issues associated to *post-selection inference*, where the same data is analysed twice: first to perform clustering and then to compare expression profiles between the clusters [@Lahnemann2020]; this is an inherent limitation of most differential expression tools.

 Furthermore, the Discussion now highlights the importance of assessing how post-selection inference may affect the performance of BASiCS (which is beyond the scope of this manuscript):

*One area for future work is to study how post-selection inference may affect the performance of `r Biocpkg("BASiCS")`. For example, @Neufeld2022 have developed a framework for addressing inflated type I errors when studying differences in means between groups of cells identified via hierarchical clustering, by splitting counts into "train" and "test" sets.*

**Reviewer comment**: I had to spend quite some time on the code chunks, working out what was going on. I think these could be decorated with useful comments so your intended user is allowed to be as lazy as possible (which is what you want so they will use your package!). This was particularly the case for the pre-processing and parameter estimation.

**Author’s response: **Indeed, clarity is an important element for this type of analysis workflows. We have now added further comments in the code chunks. We hope this helps to clarify specific steps. Furthermore, we have moved the code dealing with data QC to a separate repository, as the topic has been covered in previous F1000Research workflow articles (e.g. Lun et al 2016, https://f1000research.com/articles/5-2122 ). We hope these changes have reduced the complexity of the code and improve the flow of our article.

**Reviewer comment: **There are some quite long code chunks that I felt represented fairly repetitive tasks, mostly related to plotting. If you're using ggplot2 then you can write a function which returns a ggplot object with nice defaults and you can explain that it can be customised using the ggplot principles. I think the current style is written to allow this customization but I think it's currently unexplained and perhaps working against you. 

**Author’s response: **As mentioned above, we have moved the code dealing with data QC to a separate repository, which should reduce the complexity of the code that readers need to process. We have also attempted to reduce the complexity of later plots by splitting them into smaller chunks with better explanation, which we hope aids the readability of the manuscript.

**Reviewer comment: **In the section of parameter estimation, the discussion on priors is quite brief and quite unclear currently. I’m not quite sure what the "joint prior" is here, I think your intended audience would appreciate a narrative description of these priors. The other choices of independent log Normal is also unclear. The logMean and logSd appear to be set automatically using the data - is that what you mean by Empirical Bayes here? When I dive deeper into changing the priors BASiCS_PriorParam is quite daunting. I think there are some helpful things you can do for your readers. For example, you explain whether the analysis is sensitive to the prior choice and generally how they affect the analysis. You could guide your users through a prior predictive check, in which you simulate parameters from the prior and then fake data from the likelihood. You could then show users how well calibrated their prior choices are. General advice of setting the prior or whether they should just leave these alone would be useful.

**Authors’ response: **We agree that further information/intuition about the priors will improve our manuscript. To address this, we have now extended the model description. As noted above, we have primarily focused on the latest version of the model (Eling et al 2018) as that is the default recommended setting. In particular, we have now provided additional information to clarify the definition of our "joint prior" and the use of an empirical Bayes approach. This includes the bottom right panel in Figure 2 and edits to the “BASiCS - Bayesian Analysis of Single Cell Sequencing data” sub-section within Methods:

To account for the strong relationship that is typically observed between gene-specific mean expression and over-dispersion estimates, Eling *et al.*  [@Eling2018] introduced a *joint prior* specification for these parameters. This joint prior assumes that genes with similar mean expression ($\mu_i$) have similar over-dispersion parameters $\delta_i$. Effectively, this shrinks over-dispersion estimates towards a global trend that captures the relationship between mean and over-dispersion (Figure 2). This improves posterior inference for over-dispersion parameters when the data is less informative (e.g. small sample size, lowly expressed genes) [@Eling2018]. This information-sharing approach is conceptually similar to that performed by @Love2014 and others, where sparse data is pooled to obtain more reliable estimates. The global trend is then used to derive gene-specific *residual over-dispersion* parameters $\epsilon_i$ that are not confounded by mean expression. Similar to the DM values implemented in `r Biocpkg("scran")`, these are defined as deviations with respect to the overall trend.

 In terms of the prior hyper-parameter choices, we recommend users to apply the default settings as we have tested these in a variety of contexts. Furthermore, as shown in Vallejos et al (2015), most posterior estimates are robust to different hyper-parameter choices (the exception is for gene-specific parameters for lowly expressed genes, which benefit from the use of the joint prior introduced by Eling et al 2018 https://doi.org/10.1016/j.cels.2018.06.011 and the empirical Bayes framework). Finally, as shown by Zappia et al (2017) https://doi.org/10.1186/s13059-017-1305-0 , the BASiCS model is able to successfully recapitulate the main properties of typical scRNAseq data when used as a generative model (posterior predictive checks). To emphasise this, we have added the following text to the manuscript: 

*A previous study has shown that BASiCS, when used as a generative model, is well-tuned to capture and recapitulate the main properties of typical scRNAseq data using posterior predictive checks [@Zappia2017].*

**Reviewer comment: **I assume that multiple chains are run, are there any MCMC diagnostics based on running parallel chains?

**Authors’ response**: Due to the running times of the current MCMC sampler, we have not suggested users to routinely run multiple MCMC chains as part of their analyses. However, throughout the years, we have run BASiCS multiple times in a variety of settings and we have generally observed that different runs of the algorithm converge to similar values. Whilst this does not guarantee that the same would hold for a new dataset, our experience strongly suggests that the proposed diagnostic criteria are sufficient when evaluating MCMC convergence for BASiCS. Nevertheless, we acknowledge that running multiple chains may be useful and we will aim to implement this in future versions of the BASiCS library. In particular, we recently obtained very promising results about the use of more scalable algorithms for Bayesian inference (e.g. divide-and-conquer MCMC, see Bardenet et al. 2015 https://arxiv.org/abs/1505.02827 ; manuscript in preparation). This will facilitate running the algorithm multiple times in a timely manner. We plan to update this workflow once our benchmarks for this new, more scalable, implementation are completed and the updated BASiCS package is available in Bioconductor. We also provide the facility for users to run multiple chains and to manipulate posteriors manually using rstan ( https://bioconductor.org/packages/release/bioc/html/BASiCStan.html ), although we note that for data of any appreciable size the Hamiltonian Monte Carlo algorithm used by Stan becomes computationally intractable. We have added a brief note to the discussion regarding this, as follows:

Given that `r Biocpkg("BASiCS")` requires computationally intensive MCMC sampling to estimate the posterior distribution, it becomes computationally intractable to analyse data from very large numbers of cells. Alternative inference schemes such as variational inference for maximum *a posteriori* estimation, or Hamiltonian Monte Carlo may be useful in this context. Advanced users may wish to use the `r Biocpkg("BASiCStan")` package to test these alternative inference schemes. This also provides access to the model diagnostics, facilities for running multiple chains, and posterior summaries provided by Stan [@Carpenter2017], while also being fully compatible with the workflow described in this manuscript via the function `Stan2BASiCS`, that converts the output of the Stan inference procedure to the type of output generated by `BASiCS_MCMC`. However, we note that the Hamiltonian Monte Carlo inference method provided by Stan is more computationally intensive than our default implementation. Furthermore, the faster approximations provided by Stan, namely scalable variational inference and maximum *a posteriori* estimation, are often unstable and less accurate.

**Reviewer comment: **In a Bayesian analysis I expected to be able to manipulate posterior distributions or credible intervals. Maybe I missed it but can we visualize the full posterior distribution for \mu_i vs \delta_i and \mu_i vs \epsilon_i or some other parameters of interest?  

**Authors’ response: **As the parameter space for the BASiCS model is high-dimensional, it is not practical to visualise the joint posterior distribution posterior of all parameters. However, displaySummaryBASiCS() can be used to obtain point estimates (posterior medians) and the 95% Highest Posterior Density (HPD) intervals for individual parameters. Furthermore, the displayChainBASiCS() function can be used to extract the MCMC chains which can be then manipulated separately by the user. To clarify this, we have added the following sentence to the “Parameter estimation using BASiCS section”.  

*The matrices of MCMC draws can be accessed using the `displayChainBASiCS`*

*function --- this may be useful for estimating and visualising credible intervals using*

*packages like `r CRANpkg("bayesplot")` or `r CRANpkg("tidybayes")`.*

**Reviewer comment: **I think I haven’t quite grasped the "variance decomposition" the authors mention into biological and technical components. This could be \delta_i and \epsilon_i, but I feel I am missing something? I do not yet think the methods section is quite clear on this point.

**Authors’ response: **We used the term “variance decomposition” to denote BASiCS’ ability to separate the observed variability into a technical and a biological component, which then enables downstream analysis of transcriptional variability: detection of highly and lowly variable genes, and differential variability testing. We have thus removed the explicit use of the term “variance decomposition” from the paper, and we apologise for any confusion this caused.

**Reviewer comment: **For the volcano plots the posterior probability is quite bunched up, would this be better on the logit scale? Also It appears the probabilities concentrate around 0.5? Is there an intuitive explanation for this? Indeed, the distribution generated by hist(p2$data$ProbDiffMean) was quite unexpected, are these probabilities calibrated in any way?

**Authors’ response: **Thanks for the suggestion. We have changed these plots to use an (adjusted) logit scale, and this does appear to be more informative in most cases. The logit scale is adjusted to avoid undefined values, i.e., logit(0) and logit(1) by adding or subtracting a small value from probabilities with undefined logit values.

 In terms of calibration, this is difficult to assess in the absence of ground truth. However, as shown in Vallejos et al (2016), synthetic data examples showed good error rate performance for our differential expression test, provided that a non-zero log-fold change threshold is used (default in the BASiCS_TestDE function is shown by the vertical lines in the volcano plots). This is similar to the approach by McCarthy and Smith (2009) https://www.ncbi.nlm.nih.gov/pmc/articles/PMC2654802/ , avoiding the identification of genes with statistically significant differences but whose associated log-fold changes are not biologically significant. 

**Reviewer comment: **You mention computational challenges in the discussion, what is lost by performing maximum likelihood estimation or maximum a posteriori estimation? Perhaps the weighted likelihood bootstrap or variant could be a useful approach, if you have lots of cores.

**Authors’ response:**

 We have explored the variational inference algorithm implemented in Stan as a fast method to obtain maximum *a posteriori* point estimates for BASiCS. However, we have generally observed that inference is less reliable. Moreover, beyond point estimates, it is critical for BASiCS to obtain reliable estimates of posterior uncertainty as these are required for our differential test which relies on tail posterior probabilities. This is particularly problematic when using a mean-field approximation for the posterior, which is what is currently  used in our Stan implementation. 

 As an alternative approach to address these computational challenges, we recently explored more scalable algorithms (e.g. divide-and-conquer MCMC, see Bardenet et al. 2015 https://arxiv.org/abs/1505.02827 ) to perform inference using the BASiCS model. Our results are promising and a manuscript is in preparation. 

**Reviewer comment: **What are the challenges of applying this to scRNA data with spatial autocorrelation? It is common to now obtain expression data in such a context, does this additional confounding obstruct the model?

**Authors’ response:**

 The current BASiCS model assumes that cells are conditionally independent (given gene-specific and other global parameters). As a result our model does not account for the more complex structure that arises in spatial assays. Whilst we have not explored this yet, we hypothesise that ignoring this temporal correlation structure will likely lead to biassed over-dispersion estimates. We have now updated the Discussion to mention that the BASiCS model could be adapted to address more complex correlation structures across cells, such as those introduced by spatial assays and cohort studies in which cells are extracted from multiple donors (e.g. patients):

*Finally, we also anticipate potential extensions of `r Biocpkg("BASiCS")` to account for the more complex experimental designs. For example, in cohort studies where cells are extracted from multiple individuals, the hierarchical model could be expanded to quantify both for intra- and inter-individual transcriptional variability. As spatial transcriptomic technologies mature [@Marx2021], Bayesian hierarchical models such as the one implemented in `r Biocpkg("BASiCS")` could also be designed to incorporate spatial (and, potentially, temporal) structure (e.g. [@Aijo2019]). We intend to update this workflow as the field evolves, and as we address the issues and challenges outlined here.*

   

**Specific comments**:

**Reviewer comment:**

 In the first code chunks, in `website <- " https …” I think there is an initial space which stops this from running correctly.

**Authors’ response:**

 This is a formatting issue which should be resolved now.

**Reviewer comment:**

 Remember to include spaces after commas e.g. [a, ] rather than [a,], it is much clearer to read.

**Authors’ response:**

 Agreed, thank you for pointing this out; we have addressed this in our code formatting.

**Reviewer comment:**

 I think it would be better to load all the packages at the beginning, I had to restart my session several times to install the packages that I realized I needed x% of the way through the workflow.

**Authors’ response:**

 We initially loaded the packages when they were first used in order to emphasise their role within the analysis pipeline. However, we do acknowledge that some users may prefer to load all the packages from the start, to prevent installation issues during their analysis (note that installation issues are resolved when using the provided Docker image that contains all software requirements). To address this, all libraries are now loaded at the start of the analysis. Explanations for the use of each library are provided in relevant sections. 

**Reviewer comment:**

 In figure 4, the axis dimensions of the PCA misrepresents the variance explained. The ratio of axis dimensions should closely match the ratios of the variance explained so that the visualisation is faithful to the dimensionality reduction. 

**Authors’ response:**

 Thank you for highlighting this. We have now swapped the x- and y-axes such that the larger axis represents the larger component of variation in the data, although the x-axis is still disproportionate given the small amount of variation represented by that PC. However given the difference in scale here (20% vs 1%, we feel that directly proportional scaling would be excessive.

**Reviewer comment:**

 Constants used in the package could be saved as global variables, it’s very easy to make a typo with these conversions.

**Authors’ response:**

 We have implemented a helper function, BASiCS_CalculateERCC, to handle this conversion process. We note that the spike-in analysis workflow has been moved to the Zenodo repository.

**Reviewer comment:**

 In the chunk starting ## Moles per microliter, I had to edit the first line of the code chunk to get it to work. Perhaps it has been malformatted?

**Authors’ response:**

 This is another formatting error and should now be resolved. We note that the spike-in analysis workflow has been moved to the Zenodo repository.

**Reviewer comment:**

 \log is a symbol and should always be lower-case

**Authors’ response:**

 This has been resolved.

**Reviewer comment:**

 In the abstract, you write "strong technical noise". I don’t quite understand what "strong" is adding here. 

**Authors’ response:**

 The usage of “strong” aimed to emphasise that the level of technical noise was substantially higher than what was observed in bulk-level assays (see [3]). To clarify this, we have modified the abstract as follows:

*… but it is prone to technical noise, beyond what is observed in bulk-level assays.*

**Reviewer comment:**

 A number of concepts are left unexplained such as "joint prior", "negative binomial framework", "borrows information", "expected false discovery rate". These are all familiar concepts to someone with advanced statistical knowledge but the casual scRNA reader might find this off putting.

**Authors’ response:**

 This is an excellent suggestion. We have now edited the text to explain these concepts. 

 To explain the “negative binomial framework” we have added the following text to the Introduction:

*The negative binomial distribution is commonly used to model count data when the observed variability exceeds what can be captured by a simpler Poisson model --- this is typically referred to as over-dispersion.*

 To explain the concept behind the "borrows information" statement, the first paragraph in the “BASiCS - Bayesian Analysis of Single Cell Sequencing data” sub-section within Methods has been edited as follows:

*[…] instead of modelling expression patterns separately for each gene, `r Biocpkg("BASiCS")` shares information between all genes to robustly quantify transcriptional variability. For example, as described by [@Eling2018], pooling information across genes with similar mean expression levels helps to obtain more reliable transcriptional variability estimates. The latter is particularly helpful for lowly expressed genes and sparse datasets, where less information is available.*

 To clarify the definition of our "joint prior" statement, the third paragraph in the “BASiCS - Bayesian Analysis of Single Cell Sequencing data” sub-section within Methods has been edited to include further information:

To account for the strong relationship that is typically observed between gene-specific mean expression and over-dispersion estimates, Eling *et al.* [@Eling2018] introduced a *joint prior* specification for these parameters. This joint prior assumes that genes with similar mean expression ($\mu_i$) have similar over-dispersion parameters $\delta_i$. Effectively, this shrinks over-dispersion estimates towards a global trend that captures the relationship between mean and over-dispersion (Figure \@ref(fig:basics-schematic)). This improves posterior inference for over-dispersion parameters when the data is less informative (e.g. small sample size, lowly expressed genes) [@Eling2018]. This information-sharing approach is conceptually similar to that performed by @Love2014 and others, where sparse data is pooled to obtain more reliable estimates. The global trend is then used to derive gene-specific *residual over-dispersion* parameters $\epsilon_i$ that are not confounded by mean expression. Similar to the DM values implemented in `r Biocpkg("scran")`, these are defined as deviations with respect to the overall trend.

 In the “HVG/LVG detection using BASiCS” sub-section within the case study, we have added the following text to explain the expected false discovery rate:

*The expected false discovery rate we use is conceptually similar to false discovery rate control procedures in frequentist statistics, such as the approach of @Benjamini1995, aiming to control the proportion of false discoveries produced by the procedure.*

**Reviewer comment:**

 Figure 12 there is a typo in the legend "\log_{1}0 -> \log_{10}".

**Authors’ response:**

 This has been resolved.

**Reviewer comment:**

 In Figure 12 the dendrogram is unexplained and I’m not sure what it means in this context.

**Authors’ response:**

 This dendrogram shows the similarity between cells, but it is not required here and therefore has been removed.
